# Analysis of clinical and genomic profiles of therapy-related myeloid neoplasm in Korea

**DOI:** 10.1186/s40246-023-00458-8

**Published:** 2023-02-23

**Authors:** Jiwon Yun, Hyojin Song, Sung-Min Kim, Soonok Kim, Seok Ryun Kwon, Young Eun Lee, Dajeong Jeong, Jae Hyeon Park, Sunghoon Kwon, Hongseok Yun, Dong Soon Lee

**Affiliations:** 1 Department of Laboratory Medicine, Seoul National University College of Medicine, 101 Daehak-ro, Jongno-gu, Seoul, 03080 Republic of Korea; 2 Department of Laboratory Medicine, Chung‐Ang University Hospital, Seoul, Republic of Korea; 3 Department of Genomic Medicine, Seoul National University Hospital, 101 Daehak-ro, Jongno-gu, Seoul, 03080 Republic of Korea; 4 Cancer Research Institute, Seoul National University College of Medicine, Seoul, Republic of Korea; 5 Department of Laboratory Medicine, Seoul National University Hospital, Seoul, Republic of Korea; 6 Department of Electrical and Computer Engineering, Seoul National University, Seoul, Republic of Korea; 7 Bio-MAX Institute, Seoul National University, Seoul, Republic of Korea; 8 Genomic Medicine Institute, Seoul National University Medical Research Center, Seoul, Republic of Korea

**Keywords:** Therapy-related myeloid neoplasm, Next-generation sequencing, Germline predisposition, Somatic mutation

## Abstract

**Background:**

Therapy-related myeloid neoplasm (T-MN) rarely occurs among cancer survivors, and was characterized by poor prognosis. T-MN has germline predisposition in a considerable proportion. Here, clinical characteristics and germline/somatic variant profiles in T-MN patients were investigated, and the findings were compared with those of previous studies.

**Methods:**

A review of medical records, cytogenetic study, targeted sequencing by next-generation sequencing, and survival analysis were performed on 53 patients with T-MN at a single institution in Korea.

**Results:**

The patients were relatively younger compared to T-MN patients in other studies. Our T-MN patients showed a high frequency of complex karyotypes, −5/del(5q), and −7/del(7q), which was similar to the Japanese study group but higher than the Australian study group. The most common primary disease was non-Hodgkin lymphoma, followed by breast cancer. The detailed distributions of primary diseases were different across study groups. Seven patients (13.2%) harbored deleterious presumed/potential germline variants in cancer predisposition genes (CPG) such as *BRIP1*, *CEBPA*, *DDX41*, *FANCM*, *NBN*, *NF1*, and *RUNX1*. In the somatic variant profile, *TP53* was the most frequently mutated gene, which was consistent with the previous studies about T-MN. However, the somatic variant frequency in our study group was lower than in other studies. Adverse factors for overall survival were male sex, older age, history of previous radiotherapy, previous longer cytotoxic therapy, and −5/del(5q).

**Conclusion:**

The findings of our study corroborate important information about T-MN patients. As well as a considerable predisposition to CPG, the clinical characteristics and somatic variant profile showed distinctive patterns. Germline variant testing should be recommended for T-MN patients. If the T-MN patients harbor pathogenic germline variants, the family members for stem cell donation should be screened for carrier status through germline variant testing to avoid donor-derived myeloid neoplasm. For the prediction of the prognosis in T-MN patients, sex, age, past treatment history, and cytogenetic findings can be considered.

**Supplementary Information:**

The online version contains supplementary material available at 10.1186/s40246-023-00458-8.

## Background

Development of therapy-related myeloid neoplasm (T-MN) occurs as a late complication of cytotoxic therapy including chemotherapy and radiotherapy [1]. According to WHO classification, T-MN includes therapy-related acute myeloid leukemia (T-AML), therapy-related myelodysplastic syndrome (T-MDS), and therapy-related myelodysplastic/myeloproliferative neoplasm (T-MDS/MPN) [1]. Technological advances such as next-generation sequencing (NGS) have recently widened the understanding of the mutational profile of T-MN.

T-MN is a rare disease, accounting for up to 10–20% of newly diagnosed cases of AML or MDS [2, 3]. According to the surveillance epidemiology end results (SEER) database of the USA, the overall incidence of T-MN in the US was 0.13 cases for a population of 100,000 between 2001 and 2014 [4]. The incidence of T-MN is expected to rise as the number of cancer survivors increases due to improved outcomes for the primary malignancy [5].

Inherited predisposition, exposures to genotoxic agents, clonal selection with clonal hematopoiesis, and abnormal bone marrow (BM) microenvironment are known risk factors for T-MN [5].

As only a small percentage of patients treated with identical protocols develop T-MN, some people may have a predisposition to variants in DNA damage-sensing or repair genes (e.g., *BRCA1/2* or *TP53*) or polymorphisms in genes involved in drug metabolism, drug transport, or DNA-repair mechanisms [5]. In several studies, germline variants in inherited cancer susceptibility genes were detected in 16–21% of patients with T-MN; *BARD1, BRCA1, BRCA2, CHEK2, TP53*, and Fanconi anemia (FA) genes (*FANCA, FANCD2, FANCJ, PALB2*) [6–8].

Genotoxic agents induce DNA damage that leads to genomic transformation. The cytotoxic agents commonly implicated in T-MN include alkylating agents, ionizing radiation therapy, topoisomerase II inhibitors, antimetabolites, antitubulin agents, etc. [1, 5]. For example, alkylating agents, ionizing radiation therapy, and antimetabolites link to deletion of chromosome 5 or 7. Topoisomerase II inhibitors can induce translocations involving *KMT2A*, *RUNX1*, and *PML-RARA*. Since patients are treated with multiple chemotherapeutics, boundaries between the two major classes of T-MN (alkylating agent class and topoisomerase II inhibitor class) are unclear.

Clonal hematopoiesis of indeterminate potential (CHIP) is an emerging risk factor for hematologic malignancy. Cytotoxic therapy gives pre-existing clones with somatic variants, that is, CHIP, a competitive advantage over normal hematopoietic stem cells [5]. The clones subsequently acquire additional variants to become malignancy [5]. The malignant clones have an inherent resistance to therapy since they have undergone selection for fitness to cytotoxic pressure [5]. Patients who underwent cytotoxic therapy for the treatment of lymphoma, breast and/or ovarian cancer showed a higher frequency of clonal hematopoiesis [9–12]. Higher prevalence of CHIP in patients who developed T-MN compared to CHIP in patients who did not develop T-MN, was reported: 71% versus 31% [13]. Furthermore, the presence of CHIP increased the risk of developing T-MN by more than 13-fold [13].

Regarding the BM microenvironment, cytotoxic therapy can influence the BM microenvironment through modulation of a pro-inflammatory response, and release of inflammatory cytokines [5]. As recently reported, alterations of the BM niche play a role in the pathogenesis of myeloid neoplasm [14–17].

The somatic alteration of T-MN has mainly been investigated in Western countries, and has rarely been reported in Asia so far. The burden of somatic variants in T-MN was similar to that of de novo AML and primary MDS, however, T-MN showed a distinct mutational profile [18, 19]. A significant number of somatic variants in T-MN were observed in *TP53*, ABC family genes, and *DNMT3A* while de novo AML harbored frequent variants in *NPM1*, *FLT3*, and *TET2* [5]. In Asia, 56-gene targeted sequencing was performed on 13 T-MN patients by a Japanese research team [20]. Their T-MN cells were commonly mutated in *TP53* and *TET2*, instead of *FLT3*, *NPM1*, and spliceosome-related genes which were frequently mutated in de novo AML and MDS [20]. However, the conduct of a comprehensive variant analysis including a substantial number of patients in Asia has been rare.

Due to the scarcity of the disease, a study of T-MN requires long-term observation. Therefore, in the current study, a total of 53 patients with T-MN have been collected for eight years in a single center. We aimed to characterize the clinical and genomic profiles of the patients, and compare them with those from previous studies conducted in other countries.

## Methods

### Patient population

**Fig. 1:**
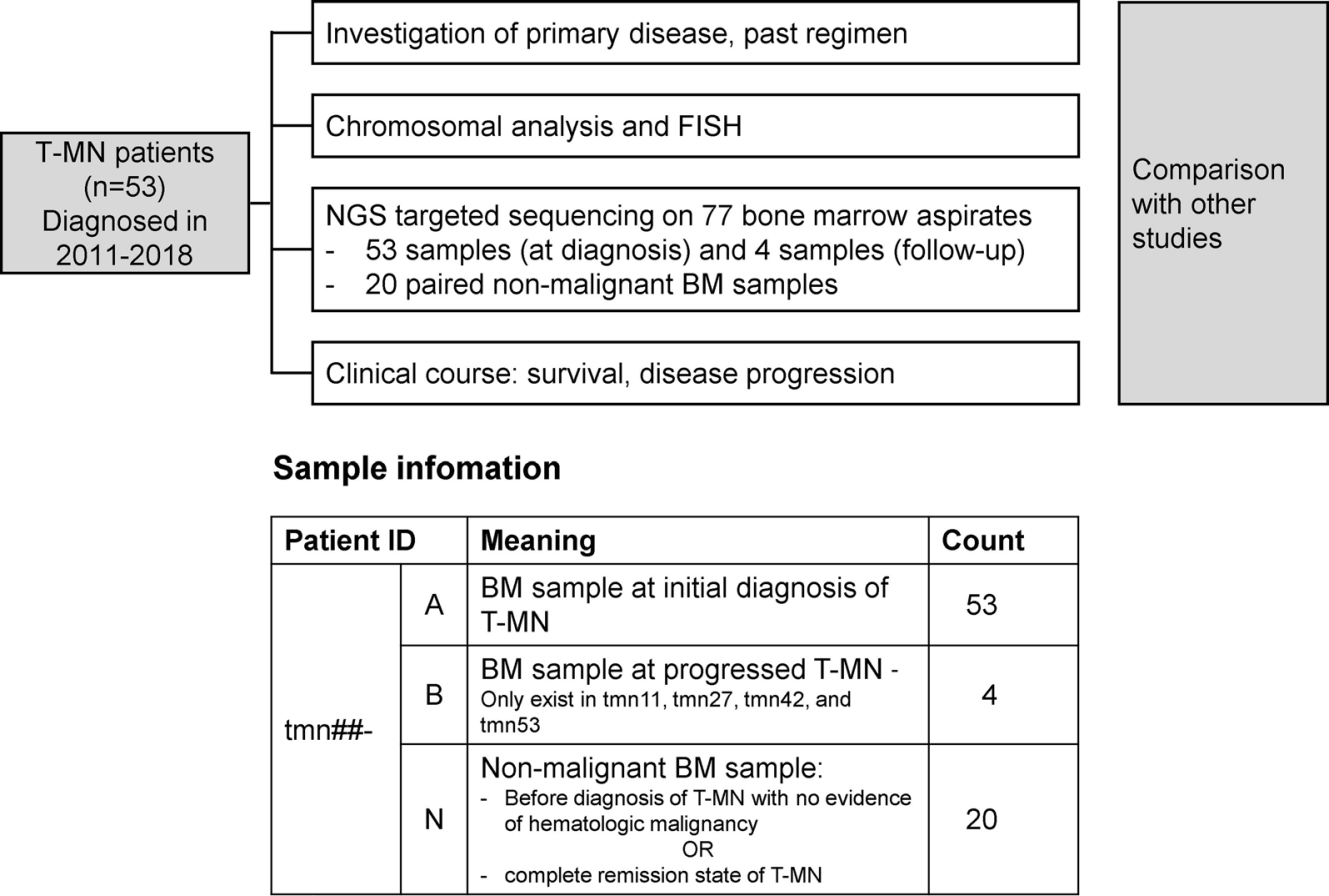
Study population and design. *T-MN* Therapy-related myeloid neoplasm; *FISH* Fluorescence in situ hybridization; *NGS* Next-generation sequencing; *BM* Bone marrow

A total of 53 patients diagnosed with T-MN between 2011 and 2018 at Seoul National University Hospital (SNUH) in Seoul, Korea, were included (Fig. 1). Analysis of 77 BM aspirates was performed for a genomic profile. Each patient had one BM sample at initial diagnosis of T-MN; designated as “patient ID-A”. Four patients had an additional BM sample at T-MN progression: designated as “patient ID-B”. Twenty patients had non-malignant BM samples for validation of presumed germline and somatic variants: designated as “patient ID-N”. The non-malignant BM samples were acquired when the patients were in complete remission from T-MN or when the patients had presented no evidence of hematologic malignancy on the BM exam before the development of T-MN.

### BM histological examination and cytogenetic studies

A review of Wright-Giemsa-stained BM smears and hematoxylin and eosin-stained sections of the BM trephine biopsies was performed by hematopathologists. Immunohistochemical staining including CD34, CD117, CD61, etc. was performed.

The conventional G-banding technique was used in the performance of chromosome analysis. The karyotype designation was based on the principles of the International System for Human Cytogenetic Nomenclature (ISCN) 2020.

Interphase fluorescence in situ hybridization (FISH) analysis was performed on mononuclear cells of BM aspirates for detection of common cytogenetic abnormalities related to AML and MDS including *RUNX1*/*RUNX1T1* rearrangement, *CBFB* rearrangement, *PML*-*RARA* rearrangement, *KMT2A* rearrangement, chromosome 5/5q deletion, chromosome 7/7q deletion, trisomy 8, 20q deletion, chromosome 1/1q gain, and *MECOM* rearrangement FISH. The results of FISH were recorded according to ISCN 2020.

### Targeted sequencing

Analysis of 77 BM aspirate samples (57 T-MN samples and 20 non-malignant samples) from 53 patients was performed. The gene panel for targeted sequencing contained 647 hematologic malignancy-related genes. Among 647 genes, 167 genes and 93 genes were selected for further analysis of germline and somatic variants, respectively (See Additional file 1: Tables S1 and S2). The 167 genes for the determination of germline variants included cancer predisposition genes (CPG) and myeloid neoplasm predisposition genes [1, 21–25]. The 93 genes for the selection of somatic variants included genes commonly observed in MDS, AML, and T-MN [1, 13, 19, 26, 27].

Genomic DNA was extracted from frozen BM mononuclear cells of all patients. The MagNA Pure LC DNA Isolation Kit (Roche Applied Science, Indianapolis, IN, USA) was used according to the manufacturer’s instructions for the extraction of DNA. Assessment of the 260/280 absorbance ratio was performed using an ND-1000 Spectrophotometer (NanoDrop Technologies, Wilmington, DE, USA) for analysis of the DNA quality. The genomic DNA was fragmented as approximately 250 bp using the Bioruptor Pico Sonication System (Diagenode, Belgium), and processed for Illumina sequencing according to the following steps; end-repair, dA-tailing, adapter ligation, and pre-PCR for indexed NGS library. Prepared gDNA library and capture probes were hybridized to capture target regions using the Celemics target enrichment kit (Celemics, Seoul, Republic of Korea). Capture probes were designed and chemically synthesized to hybridize the target region. Further amplification of captured regions was then performed using post-PCR to enrich the amount of sample. Sequencing of the target-captured library was then performed on an Illumina NextSeq550 instrument (Illumina, San Diego, CA, USA) using the read layout 2 × 150 bp.

### Variant calling

Analysis of the FASTQ files generated from the customized panel sequencing was performed using the SNUH FiRST Panel Analysis Pipeline. The quality control of the FASTQ files and further steps in the analysis was performed in the data which accomplished the criteria; average Phred quality score per base at least 20. The statistical metrics calculated in the whole samples utilized in this study are shown in Additional file 1: Fig. S1. Paired-end alignment to the hg19 reference genome was subsequently performed using BWA-mem (v0.7.17) and GATK Best Practice [28, 29]. From the alignment step, “Analysis-ready” BAM files were produced, and detection of variants such as single-nucleotide variants (SNV) and small insertions–deletions (InDel) was performed using more than two analytic tools, including the SNUH in-house pipeline and open-source software. GATK UnifiedGenotyper (v4.1.9), SNVer (v0.5.3), and LoFreq (v2.1.2) were used for detection of SNV/InDel [29, 30].

### Variant filtering

**Fig. 2:**
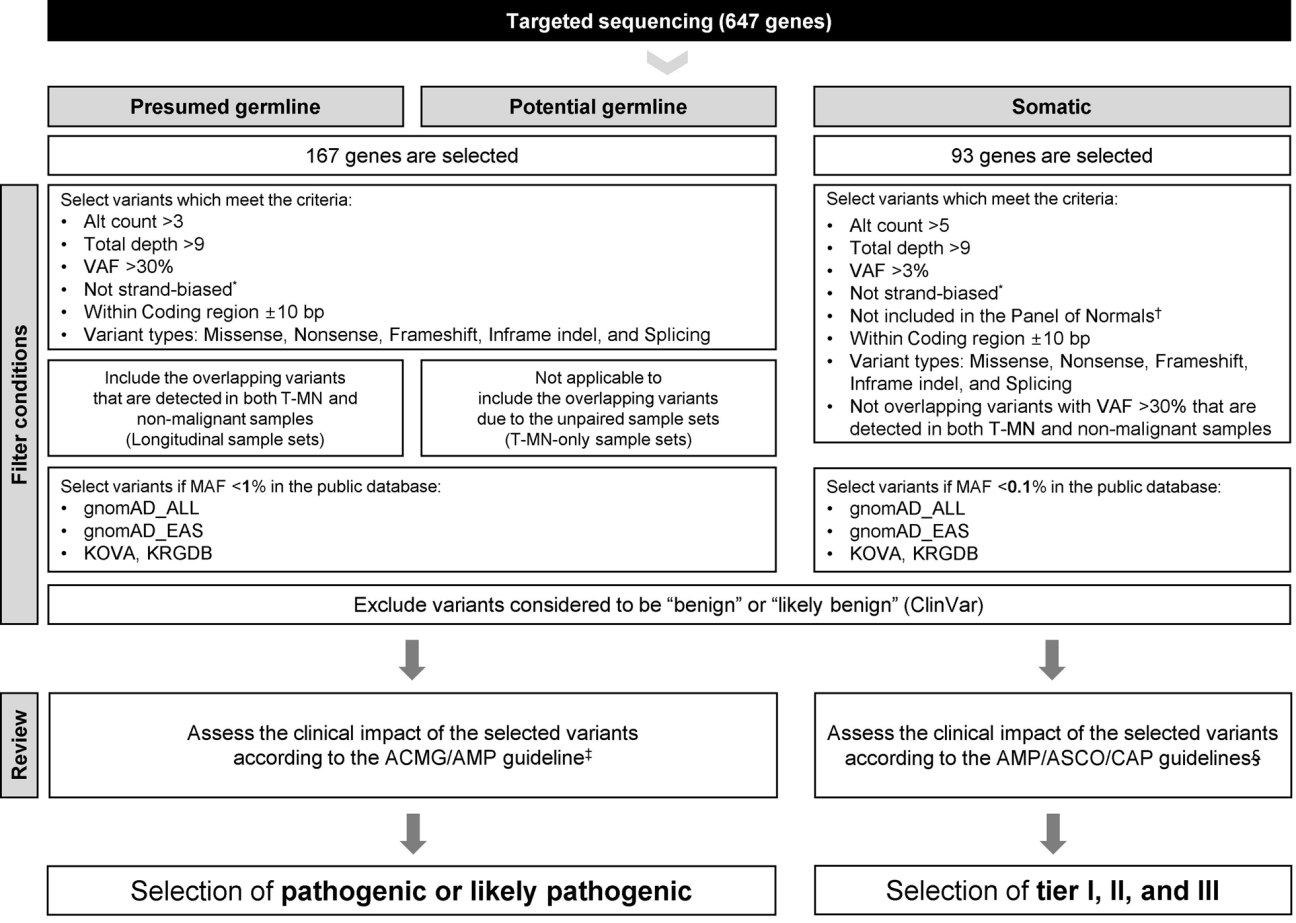
Variants filtering strategy. *The Strand-biased variants refer to the variants whose forward and reverse stranded alternative counts have a ratio of either 2:8 or 8:2. ^†^The Panel of Normals approach was used to determine the selection criteria for variants for somatic variants, with a threshold of variant frequency (refer to https://gatk.broadinstitute.org/hc/en-us/articles/360035890631-Panel-of-Normals-PON-). *VAF* Variant allele frequency; *MAF* Minor allele frequency; *gnsomAD* The genome aggregation database; *KOVA* Korean variant archive; *KRGDB* Korean reference genome database; *DM* Disease-causing variant, *HGMD* The human gene variant database; gnomAD_genome/exome_ALL; gnomAD_genome/exome_EAS, The allele frequencies from all populations and East Asian population, respectively through genome/exome sequencing

From the variants that were selected preliminarily, the variants were retained according to our strategy for filtering variants shown in Fig. 2. Annotation of the detected variants was performed using SnpEff (v5.0) and various databases including RefSeq, COSMIC (v84), dbSNP (build 150), ClinVar (last accessed June 16, 2022), gnomAD (v2.0.1), and HGMD professional (2021–04) [31–37].

Using our strategy, filtered SNV and InDel were classified as presumed germline, potential germline, and somatic variants (Fig. 2). Non-malignant BM samples were used for validation of presumed germline variants. Although rarely, the risk of confounding due to clonal hematopoiesis could not be excluded if their VAFs (variant allele frequencies) are not low [38]. Some variants could be classified as both potential germline and somatic variants. Assessment of germline variants was performed according to the American College of Medical Genetics and Genomics and the Association for Molecular Pathology (ACMG/AMP) guidelines [39]. PVS1 criterion was evaluated with recommendations of ClinGen Sequence Variant Interpretation Workgroup [40]. For satisfying PM2 criterion, gnomAD exome global cut-off < 0.00001 for dominant disease, and < 0.0001 for recessive disease was applied [38]. For applying PP3 criterion, the concordance of three prediction tools was used: SIFT, D; PolyPhen2_HVAR, D or P; and CADD score > 20. Finally, pathogenic or likely pathogenic variants were selected. Assessment of the somatic variants was performed according to the Association for Molecular Pathology, American Society of Clinical Oncology, and College of American Pathologists (AMP/ASCO/CAP) guidelines [41], and tier I, II and III variants were selected. SpliceAI [42], a deep learning-based tool, was used for validation of the splicing variants detected from the in-house pipeline.

### Clinical characteristics and comparison with other study groups

The clinical characteristics of 53 T-MN patients including age at diagnosis, sex, primary disease treated with cytotoxic therapy, prior treatment regimen, and latency were investigated. They were also compared with those of the Singhal, SEER, and Nishiyama study groups about T-MN patients conducted in South Australia, USA, and Japan, respectively [4, 19, 20].

### Somatic variant profile and comparison with other study groups

The landscapes of somatic variant profiles of this study (SNUH) and other study groups were investigated and painted. Two major groups consisting of T-MN patients were used in the comparison of the somatic variant frequency: the Singhal [19] study group and the cBioPortal study group. The cBioPortal study group, which was publicly available via the cBioPortal, originated from several main study groups [43–45] conducted at Oregon Health & Science University and Memorial Sloan Kettering Cancer Center. The races of patients in both the Singhal and cBioPortal study groups were not thoroughly specified. The dominant race of both study groups was assumed to be Caucasian considering the countries where the studies were exerted.

On the other hand, SEER and Nishiyama study groups were not used for comparison of somatic variant profile. SEER study did not present somatic variant analysis, and Nishiyama study group had a too small population to analyze.

Forty-three genes were used for the comparison of somatic variants among the three study groups (See Additional file 1: Table S3). The 43 genes were overlapped genes for analysis of somatic variants in the three groups.

### Statistical analysis

The characteristics of patients with continuous variables were expressed as medians and ranges or interquartile ranges. The characteristics of patients with categorical variables were presented as numbers and percentages. Testing for normality for continuous variables was performed using D'Agostino & Pearson, Shapiro–Wilk, and Kolmogorov–Smirnov normality tests. The Mann–Whitney test (between two groups) and Kruskal–Wallis test (among three or more groups) were used for the comparison of continuous variables. The Chi-square test or Yates' continuity corrected Chi-square test (between two groups) and the Chi-square test (among three groups) were used for comparison of categorical variables. Evaluation of pairwise correlations between gene variants, gene categories, and cytogenetic results was performed using Pearson correlation. The Kaplan–Meier survival analysis (log-rank test) was used for the comparison of survival. Assessment of the predictive value for overall survival (OS) was performed using Cox proportional hazard analysis. All statistical analyses were performed using GraphPad Prism 7 (GraphPad, San Diego, CA, USA) and R software version 4.1.2. *P* value < 0.05 was considered statistically significant.

## Results

### Clinical characteristics of the study population and comparison with other study groups

The 53 T-MN patients of the SNUH group consisted of 28 T-MDS, one T-MDS/MPN, and 24 T-AML patients (Table 1). Among 28 T-MDS patients, three patients were also diagnosed with donor-derived MDS. The pathologic diagnosis of the T-MDS/MPN was chronic myelomonocytic leukemia. The median age of patients was 56.5 and 60.3 years old when including and excluding seven pediatric patients (aged below 20 years old), respectively. No sexual dominance was observed in the SNUH group, and the major race was Korean with two non-Korean pediatric patients (tmn47, Kazakh; tmn48, Russian).

Primary diseases were defined as the previous disease treated with cytotoxic therapy. Non-Hodgkin lymphoma (30.2%) was the most common primary disease for patients in the SNUH group when including pediatric patients, followed by breast cancer (15.1%), AML (9.4%), bone/soft tissue neoplasm (9.4%), germ cell tumor (9.4%), etc. The primary diseases of the seven pediatric patients were bone/soft tissue neoplasm (*n* = 4), germ cell tumor (*n* = 2), and others (*n* = 1).

A high number of unbalanced chromosomal abnormalities including −5/del(5q) (28.3%) and −7/del(7q) (43.4%) was observed in the SNUH group. In addition, the two cytogenetic aberrations tended to coexist (Chi-square = 8.727, *P* = 0.003). Complex karyotype indicating more than two cytogenetic abnormalities accounted for almost half of the patients (47.2%). Balanced translocations including *PML*-*RARA* (7.5%), *KMT2A* rearrangement (7.5%), *RUNX1* rearrangement (1.9%), and *MECOM* rearrangement (5.7%) were also observed, though less frequently than unbalanced chromosomal abnormalities. The median latency from the initiation of cytotoxic therapy to the development of T-MN was 52.0 months. The median latency of patients harboring balanced translocation was 28.2 months, whereas the median latency of the patients whose karyotyping results were abnormal but did not contain balanced translocation was 60.5 months (*P* = 0.080).

Apart from the SNUH study, the Singhal (*n* = 129), SEER (*n* = 1,093), and Nishiyama (*n* = 13) study groups consisted of only adult patients with T-MN. Excluding the seven pediatric patients, the median age of patients in the SNUH study group was the youngest among the four studies (60.3 years old in SNUH, 71.1 years old in Singhal, and 65 years old in both SEER and Nishiyama). The Singhal group was slightly dominant with male patients (56.6%), but the SEER group with female patients (55.5%). While the detailed distributions of primary diseases were different across the four study groups, the most common primary disease was the same as non-Hodgkin lymphoma (30.2–46.2%). Except for the Nishiyama study group, the second most common primary disease was breast cancer (12.4–20.6%). In detail, the SNUH group harbored a higher proportion of AML in primary disease compared to the others (9.4% vs. 0–0.8%). The Korean and Japanese groups harbored more germ cell tumor compared to the Singhal group (9.4–15.4% vs. 0.8%). Sole autoimmune disease (10.1%) and prostate cancer (10.9%) as primary diseases were frequently observed in the Singhal study group.

Regarding past cytotoxic therapy, the SNUH group had no patient who underwent sole radiotherapy, but 21.7% of Singhal group patients were previously treated with only radiotherapy. Among chemotherapeutics that induce T-MN, an alkylating agent was the most frequently used in both SNUH and Nishiyama groups, followed by topoisomerase II inhibitor. The patients in SNUH study group (67.9%) were more treated with topoisomerase II inhibitor than the Nishiyama study group (15.4%).

Compared to Singhal and Nishiyama groups, a higher proportion of T-AML was observed in the SNUH group (45.3% vs. 15.4–26.4%). −5/del(5q), −7/del(7q), and complex karyotype were more frequently observed in Korean and Japanese study groups compared to the Singhal study group: −5/del(5q), 28.3–30.8% versus 3.9%; −7/del(7q), 30.8–43.4% versus 17.8%; and complex karyotype, 47.2–61.5% versus 27.9%.

**Table 1:** Clinical characteristics of 53 T-MN patients and comparison with other study groups

Characteristic	SNUH (*n* = 53)	Singhal (*n* = 129)	SEER (*n* = 1093)	Nishiyama (*n* = 13)
Age at diagnosis (years)	Inclusion of pediatric patients* Yes: 56.5 (1.8–82.4) No: 60.3 (20.7–82.4)	71.1 (20.7–89.9)	65 (20–97)	65 (24–76)
Age group				
< 20	7 (13.2)	0		0
20–39	5 (9.4)	NA	68 (6.2)	4 (30.8)
40–59	18 (34.0)	NA	317 (29.0)	2 (15.4)
60–79	20 (37.7)	NA	606 (55.4)	7 (53.8)
≥ 80	3 (5.7)	NA	102 (9.3)	0
Sex				
Male	28 (52.8)	73 (56.6)	486 (44.5)	NA
Female	25 (47.2)	56 (43.4)	607 (55.5)	NA
Race	Korean: 51 (96.2) Others: 2 (3.8)	NA	White: 963 (88.1) Black: 69 (6.3) Other: 61 (5.6)	NA
Primary disease				
Non-Hodgkin lymphoma^†^	16 (30.2)	49 (40.0)	341 (33.8)	6 (46.2)
Plasma cell dyscrasia (plasma cell myeloma, etc.)	4 (7.5)	11 (8.5)	52 (5.2)	2 (15.4)
Chronic lymphocytic leukemia	1 (1.9)	5 (3.9)	31 (3.0)	0
Breast cancer	8 (15.1)	16 (12.4)	208 (20.6)	0
Others	7 (13.2)	10 (7.8)	313 (30.6)	3 (23.1)
Acute myeloid leukemia	5 (9.4)	1 (0.8)	NA	0
Bone/soft tissue neoplasm	5 (9.4)	3 (2.3)	43 (4.3)	2 (15.4)
Germ cell tumor	5 (9.4)	1 (0.8)	NA	2 (15.4)
Colorectal cancer	4 (7.5)	9 (7.0)	NA	0
Multiple primary tumors	2 (3.8)	6 (4.7)	NA	0
Acute lymphoblastic leukemia	1 (1.9)	4 (3.1)	36 (3.6)	0
Sole autoimmune disease	0	13 (10.1)	NA	0
Hodgkin lymphoma	0	3 (2.3)	30 (2.9)	0
Prostate cancer	0	14 (10.9)	43 (4.2)	0
Latency (months)	52.0 (12.0–309.7)	NA	NA	12 (4–48)
Duration of past cytotoxic therapy (months)	18.6 (1.2–122.8)	NA	NA	NA
Past cytotoxic therapy			Chemotherapy Yes: 750 (68.6) No/unknown: 343 (31.4)	
Sole chemotherapy	35 (66.0)	59 (45.7)	NA	NA
Sole radiotherapy	0	28 (21.7)	NA	NA
Chemotherapy and radiotherapy	18 (34.0)	42 (32.6)	NA	NA
Alkylating agent	45 (84.9)	NA	NA	12 (92.3)
Topoisomerase II inhibitor	36 (67.9)	NA	NA	2 (15.4)
Antimetabolite	9 (17.0)	NA	NA	NA
Antitubulin agent	25 (47.2)	NA	NA	NA
Autologous PBSCT	9 (17.0)	21 (16.3)	NA	NA
Allogenic PBSCT	3 (5.7)	NA	NA	NA
T-MN subtype				
MDS	28 (52.8)	83 (64.3)	NA	9 (69.2)
MDS/MPN	1 (1.9)	11 (8.5)	NA	2 (15.4)
AML	24 (45.3)	34 (26.4)	NA	2 (15.4)
Cytogenetic abnormalities^‡^				
−5/del(5q)	15 (28.3)	5 (3.9)	NA	4 (30.8)
−7/del(7q)	23 (43.4)	23 (17.8)	NA	4 (30.8)
−17/del(17p)	5 (9.4)	NA	NA	1 (7.7)
* PML*-*RARA*	4 (7.5)	2 (1.6)	NA	0
* KMT2A* rearrangement^§^	4 (7.5)	4 (3.1)	NA	0
* RUNX1* rearrangement	1 (1.9)	NA	NA	0
* MECOM* rearrangement	3 (5.7)	NA	NA	1 (7.7)
Chromosome 3 abnormalities^§^	0	2 (1.6)	NA	0
Complex karyotype (≥ 3 abnormalities)	25 (47.2)	36 (27.9)	NA	8 (61.5)
Normal karyotype	6 (11.3)	NA	NA	2 (15.4)
Not assessed	2 (3.8)	NA	NA	0
Overall survival (months)	Inclusion of pediatric patients* Yes: 10.6 (0.2–102.4) No: 9.2 (0.2–102.4)	10.6 (9.1–13.6)	NA	NA

Data are presented as numbers (percentages) or median values (ranges)

*T-MN* Therapy-related myeloid neoplasm; *PBSCT* Peripheral blood stem cell transplantation; *AML* Acute myeloid leukemia; *MDS* Myelodysplastic syndrome; *MDS/MPN* Myelodysplastic/myeloproliferative neoplasm; *NA* Not assessed; *−5/del(5q)* Chromosome 5 or 5q deletion; *−7/del(7q)* Chromosome 7 or 7q deletion; *−17/del(17p)* Chromosome 17 or 17p deletion

*The seven pediatric patients aged below 20 years old

^†^Non-Hodgkin lymphoma includes plasma cell dyscrasia and chronic lymphocytic leukemia

^‡^Several cytogenetic abnormalities coexisted in some patients. In particular, −5/del(5q) and −7/del(7q) tended to coexist

^§^Chromosome 3 abnormalities and 11q23 translocations are presented which occur alone or in combination with other chromosomal abnormalities excluding −5/del(5q), −7/del(7q), or complex karyotype

### Identified presumed/potential germline variants

A total of seven pathogenic/likely pathogenic presumed/potential germline variants were detected in seven patients (13.2%) among 53 T-MN patients (Table 2). The *DDX41*, *FANCM*, *NBN*, and *RUNX1* variants were presumed germline variants via non-malignant BM samples.

The remaining three variants are potential germline variants. Among them, *BRIP1* gene is included in only 167 genes for the detection of germline variants. *NF1* and *CEBPA* genes are included in both the 167 genes and the 93 genes for the detection of somatic variants. Although the *NF1* L532R *and CEBPA* G268fs were not reported in Catalogue of Somatic Variants in Cancer (COSMIC), *NF1* and *CEBPA* are quite observed in somatic variant profiles in myeloid neoplasm. Germline variants of *CEBPA* are usually found in the 5’ end of the gene [1]. In tmn30 case, the *CEBPA* variant was relatively placed near 5’ end rather than 3’ end.

**Table 2:** Deleterious presumed/potential germline variants identified among 53 T-MN patients

Patient ID	Sex/age	Gene and RefSeq	Syndrome (inheritance)	Coding DNA sequence	Amino acid change	VAF/total depth	ClinVar^‡^/HGMD^§^	gnomAD exomes frequency (global/ea-st asians)	ACMG classif-ication
tmn01	M/67	*BRIP1* NM_032043.3	Fanconi anemia (AR)	c.1794+1G>A	–	0.55/164	LP (★★)/not reported	0.00000796/0.000109	P (PVS1 + PM2 + PP5)
tmn12^†^	M/53	*NF1* NM_000267.3	Neurofibromatosis (AD)	c.1595T>G	p.Leu532Arg	0.68/81	P (★)/DM (high)	0/0	LP (PM1 + PM2 + PP3 + PP5)
tmn30^†^	M/54	*CEBPA* NM_004364.4	Familia AML (AD)	c.801_802delCG	p.Gly268fs (not anticipated to occur NMD)	0.35/17	None/none	0/0	LP (PVS1_Strong + PM2)
tmn36*	F/53	*FANCM* NM_020937.4	Fanconi anemia (AR)	c.1972C>T	p.Arg658* (NMD)	0.44/296	Conflicting interpretations of pathogenicity (★)/DM (high)	0.0000757/0	P (PVS1 + PM2 + PP5)
tmn40*	F/62	*DDX41* NM_016222.4	Familial myeloproliferative/lymphoproliferative neoplasms (AD)	c.308_309delAG	p.Glu103fs (NMD)	0.50/111	None/none	0/0	LP (PVS1 + PM2)
tmn49*	M/17	*RUNX1* NM_001754.4	Familial platelet disorder with propensity to myeloid malignancy	c.39C>G	p.Tyr13* (NMD)	0.46/426	None/none	0/0	LP (PVS1 + PM2)
tmn52*	M/17	*NBN* NM_002485.4	Nijmegen breakage syndrome (AR)	c.2206G>T	p.Glu736* (not anticipated to occur NMD)	0.54/162	US (★★)/DM? (low)	0.000004/0.0000544	LP (PVS1_Strong + PM2)

*RefSeq* Reference sequence; *AD* Autosomal dominant; *AR* Autosomal recessive; *NMD* Nonsense mediated decay; *VAF* Variant allele frequency; *HGMD* The human gene variant database; *gnomAD* The genome aggregation database; *ACMG* American College of Medical Genetics and Genomics; *P* Pathogenic; *LP* Likely pathogenic; *US* Uncertain significance; *DM* Disease causing variant; *DM?* Likely disease causing variant; *VUS* Variants of uncertain significance; *PVS* Pathogenic very strong; *PM* Pathogenic moderate; *PP* Pathogenic supporting

*Presumed germline variants via non-malignant BM samples

^†^Classified as both germline and somatic variants since these variants can be both

^**‡**^ClinVar reports were described with clinical significance and review status. The number of stars (★) refers to the review status of ClinVar: criteria provided, conflicting interpretations (★); criteria provided, multiple submitters, no conflicts (★★)

^**§**^HGMD reports were described with variant class and confidence

### Somatic variant profile

**Fig. 3:**
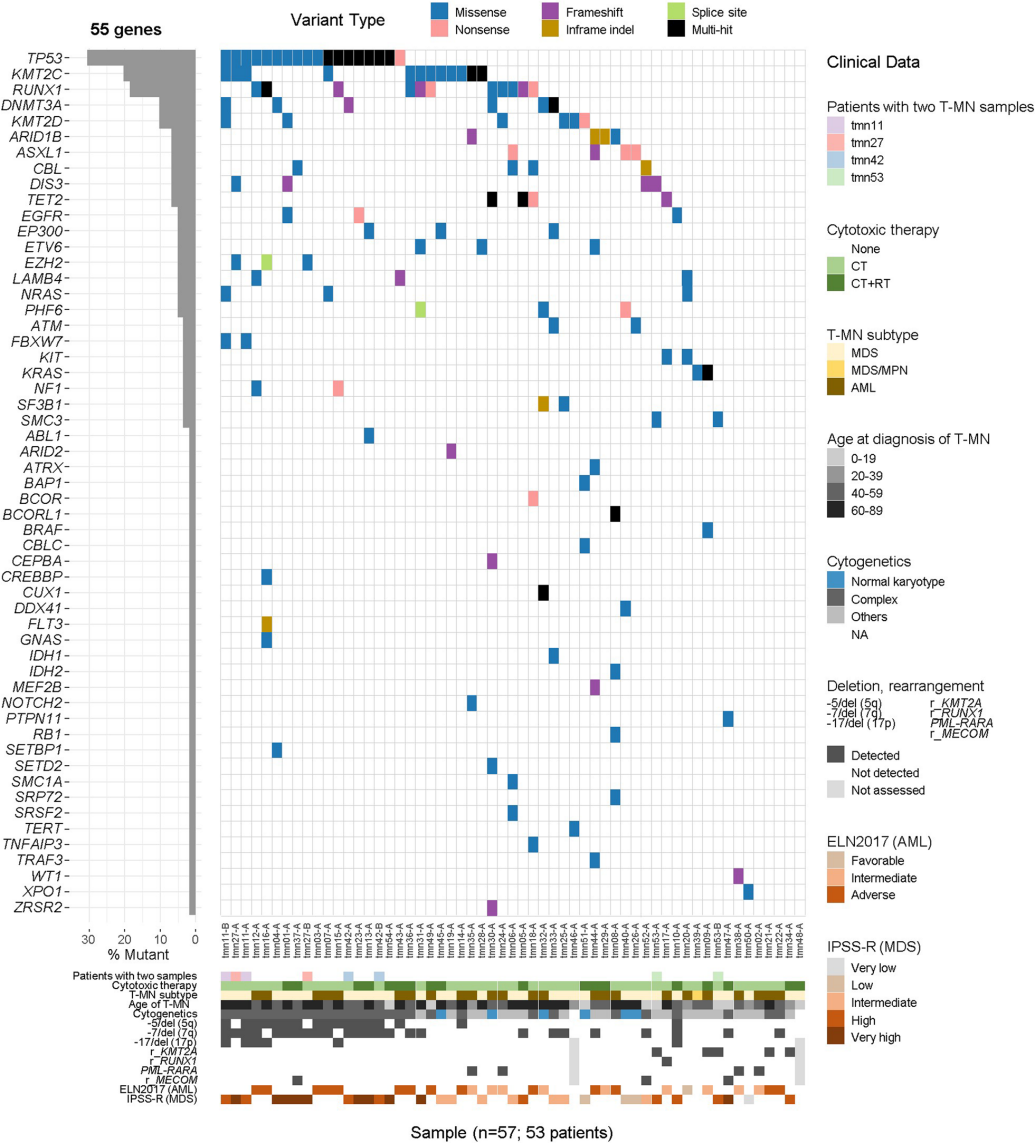
Landscape of somatic variants observed in 53 T-MN patients. This plot summarizes the landscape of somatic single nucleotide variants and small insertions–deletions variants observed in 53 T-MN patients in the SNUH study group using 93-gene filtering. The bottom of the plot illustrates the clinical characteristics of the patients and cytogenetic results. “Cytotoxic therapy” indicated previous therapy before the occurrence of T-MN. *CT* Chemotherapy; *RT* Radiotherapy; *MDS* Myelodysplastic syndrome; *MDS/MPN* Myelodysplastic/myeloproliferative neoplasm; *AML* Acute myeloid leukemia; *r* Rearranged; *NA* Not assessed; ELN2017, AML risk stratification by genetics suggested by 2017 European LeukemiaNet (ELN) recommendations from an international expert panel; IPSS-R, revised International Prognostic Scoring System (IPSS-R) for MDS assessment

The landscape of somatic SNV/InDel variants of 53 T-MN patients is shown in Fig. 3. The observed somatic variants are listed in Additional file 2. Somatic SNV/InDel variants were detected in 52 of 57 samples (91.2%) and 48 of 53 patients (90.6%) across 55 genes. The median somatic variant count per sample was 2 (range 0–7) in which multi-hit variants in a single gene were not counted as a single variant but as multiple variants. Excluding the samples from pediatric patients, the median somatic variants count per sample was 3 (range 0–7). *TP53* was the most frequently mutated gene, followed by *KMT2C*, *RUNX1*, *DNMT3A*, *KMT2D*, *ARID1B*, *ASXL1*, *CBL*, *DIS3*, *TET2*, etc. The various clinical characteristics are described at the bottom of Fig. 3. Younger patients harbored fewer variants: two variants (median value in pediatric patients, range 0–2) versus three variants (median value in adult patients, range 0–7) (*P* = 0.027).

**Fig. 4:**
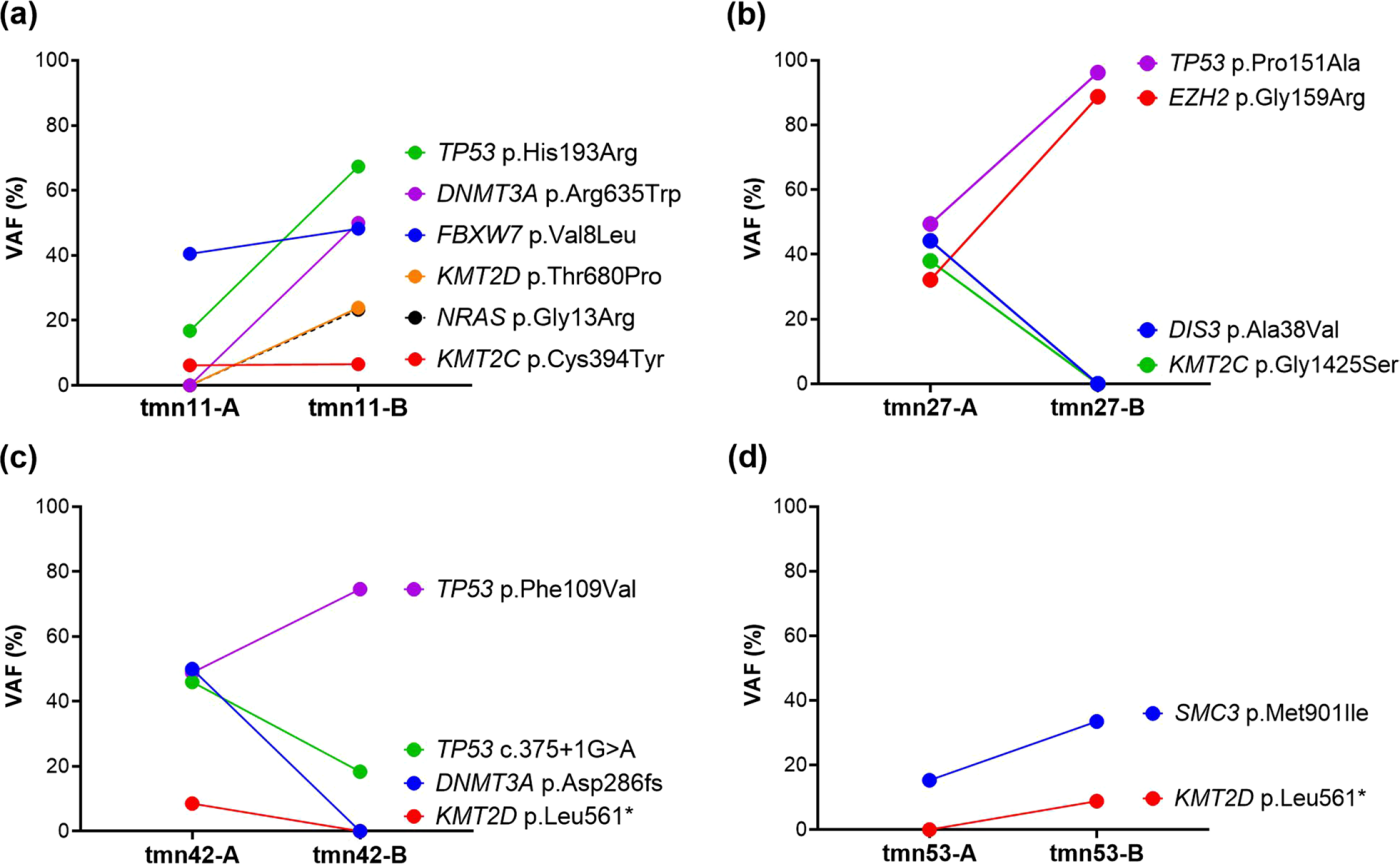
Changes in somatic variants in the progression of T-MN in four patients harboring longitudinal samples. This plot exclusively shows the changes in somatic variant profiles observed in the four T-MN patients with longitudinal samples. **a** tmn11. **b** tmn27. **c** tmn42. **d** tmn53. “Patient ID-A” samples were initial BM samples diagnosed with T-MDS. “Patient ID-B” samples were subsequent BM samples at the progression of T-MDS

Changes in variants in the progression of T-MN were observed in four patients with longitudinal T-MN samples. Somatic variants observed in the longitudinal samples are shown separately in Fig. 4. There were emerging or increased burdens of variants suggesting positive clonal selection, whereas diminished or decreased burdens of variants indicated negative clonal selection. Positively selected clones were described as follows: clones with *TP53*, *DNMT3A*, *KMT2D*, and *NRAS* variants in tmn11; clones with *TP53* and *EZH2* variants in tmn27; clones with *TP53* Phe109Val variant in tmn42; and clones with *SMC3* variant in tmn53. Negatively selected clones were depicted as follows: clones with *DIS3* and *KMT2C* variants in tmn27; clones with *TP53* c.375+1G>A and *DNMT3A* variants in tmn42; and clones with *DIS3* variant in tmn53.

The detected 55 genes were grouped according to gene function (See Additional file 3: Table S4). The genes related to chromatin modification were most frequently mutated (56.6%), followed by genes for transcription factors (54.7%), RAS pathway (24.5%), receptor/kinases (17.0%), DNA repair/cell cycle (13.2%), splicing factor (7.5%), and cohesin complex (3.8%) among 53 T-MN patients. The associations between cytogenetic abnormalities, somatic *TP53* variant, and somatically mutated gene categories are shown in Additional file 3: Fig. S2. *TP53* mutation, complex karyotype, −5/del(5q), −7/del(7q), and variants for transcription factor genes were significantly associated with each other (*r,* 0.301–0.820; *P* ≤ 0.029). The significant correlation between *TP53* mutation and mutated genes for transcription factors was attributed that *TP53* was included in genes for transcription factors. Also, −5/del(5q) showed association with variants in receptor or kinase genes (*r* = 0.332, *P* = 0.015). Loss of 17 or del(17p) was associated with *TP53* mutation (*r* = 0.492, *P* < 0.001), complex karyotype (*r* = 0.357, *P* = 0.010), −5/del(5q) (*r* = 0.470, *P* < 0.001), and mutated genes for RAS pathway (*r* = 0.287, *P* = 0.041). There was an association in somatic mutation between gene categories: splicing factor gene and cohesion complex genes (*r* = 0.281, *P* = 0.042); chromatin modification genes transcription factor genes (*r* = 0.285, *P* = 0.039); and chromatin modification genes and RAS pathway genes (*r* = − 0.294, *P* = 0.033).

### Comparison of somatic variant frequency between study groups

**Fig. 5:**
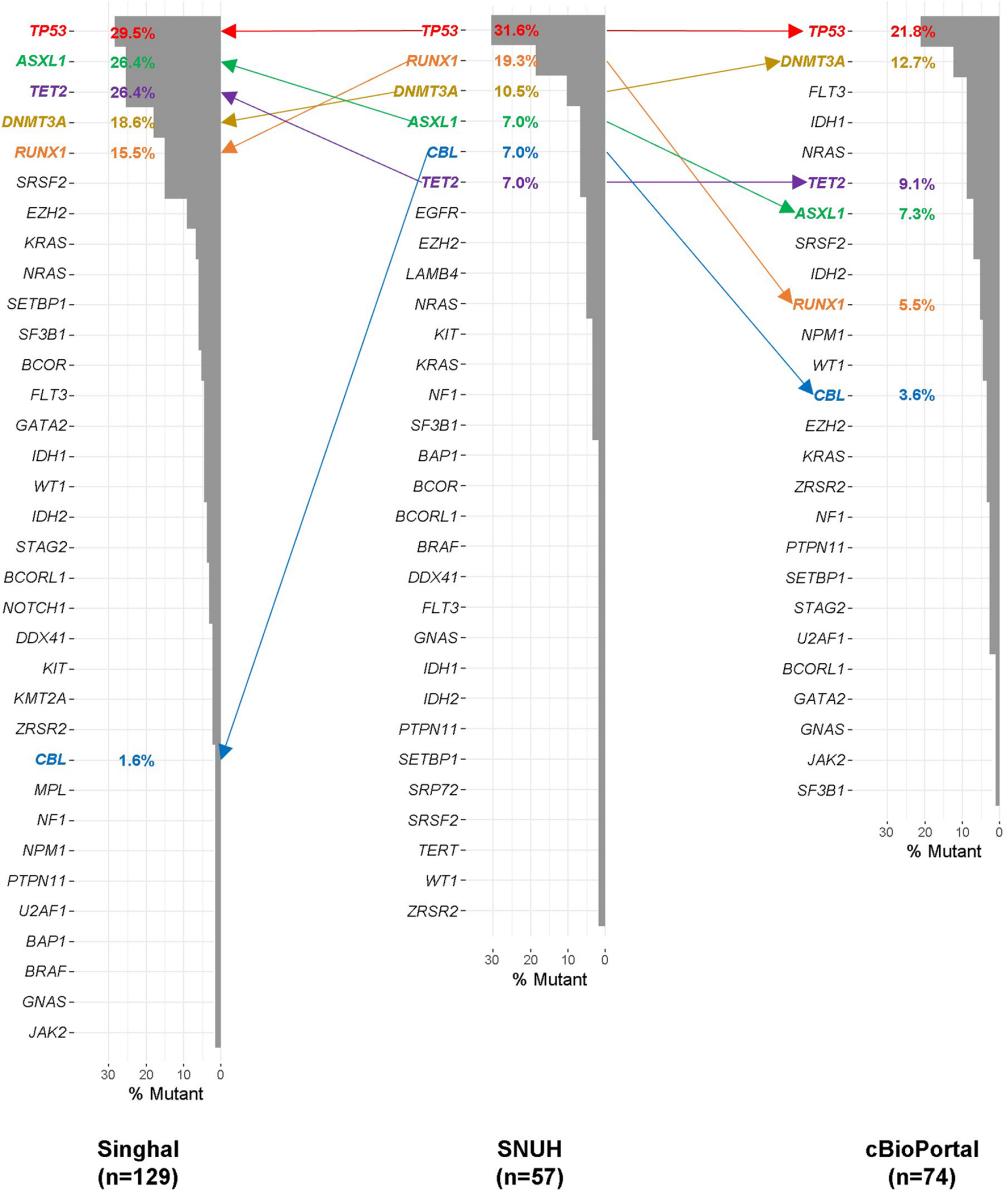
Comparison of somatic variants frequency among the SNUH, Singhal, and cBioPortal groups within 43 genes. This plot summarizes the frequency of somatic variants observed in the three study groups within 43 genes commonly analyzed in the three groups. Mutated genes observed in each study group are listed in descending order of the variant frequency. The high-ranked genes (*TP53*, *RUNX1*, *DNMT3A*, *ASXL1*, *CBL*, and *TET2*) observed in the SNUH group are indicated in colored with variant frequency

The somatic variant frequency in the 43 genes in the SNUH, Singhal, and cBioPortal study groups was investigated (See Additional file 3: Fig. S3). The somatic variant frequencies of the SNUH, Singhal, and cBioPortal groups were 75.4% (43 of 57 samples), 93.0% (120 of 129 samples), and 90.5% (67 of 74 samples) respectively. The median variant counts per sample were one (range 0–6), two (range 0–6), and two (range 0–8) in the SNUH, Singhal, and cBioPortal groups, respectively (*P* < 0.001). The Singhal group harbored more variants than the SNUH group (*P* < 0.001). Of note, *TP53* was most frequently mutated in all three study groups (Fig. 5, 21.8–31.6%), followed by *RUNX1*, *DNMT3A*, *ASXL1*, *CBL*, and *TET2* in the SNUH group. Except for *CBL*, these five genes were also ranked highly in the Singhal study group and their variant frequencies were higher than those of the SNUH group. The cBioPortal group harbored a lower variant frequency for *RUNX1* (5.5%) compared to the other study groups (15.5–19.3%). *CBL* variants were more frequently observed in the SNUH group (7.0%) compared to the other study groups (1.6–3.6%).

**Fig. 6:**
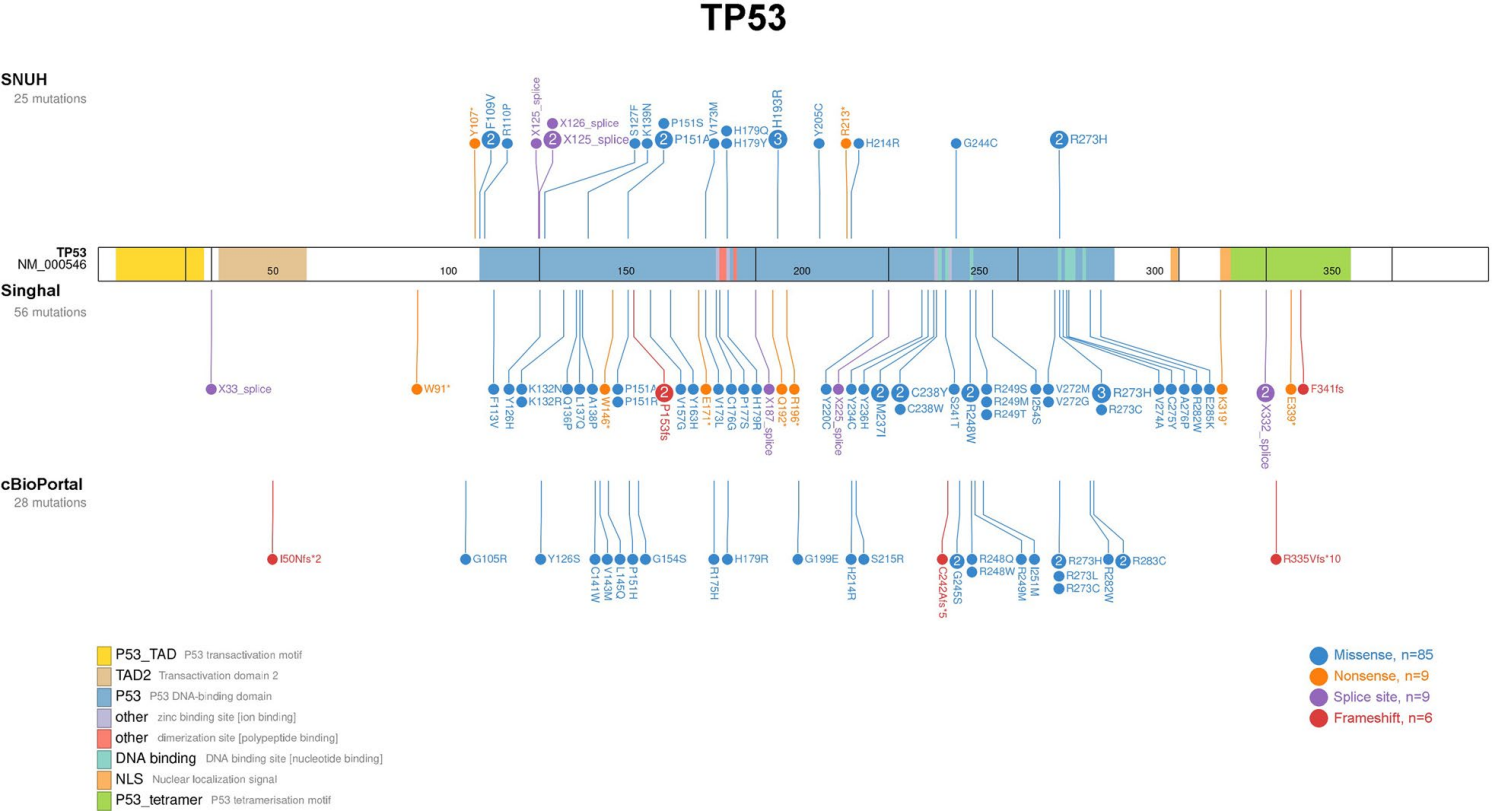
Distribution of somatic *TP53* variants in the SNUH, Singhal, and cBioPortal study groups. This lollipop plot describes the distribution of *TP53* variants according to protein domain. Lollipop plots for the other 37 genes are shown in Additional file 4

A comparison of the distributions of the somatic variants of *TP53* between the three study groups was performed (Fig. 6). Most of the *TP53* somatic variants were observed in the P53 domain which functions as the DNA-binding domain in all three groups. The distribution plots of the remaining genes are shown in Additional file 4.

### Prognostic factors

**Fig. 7:**
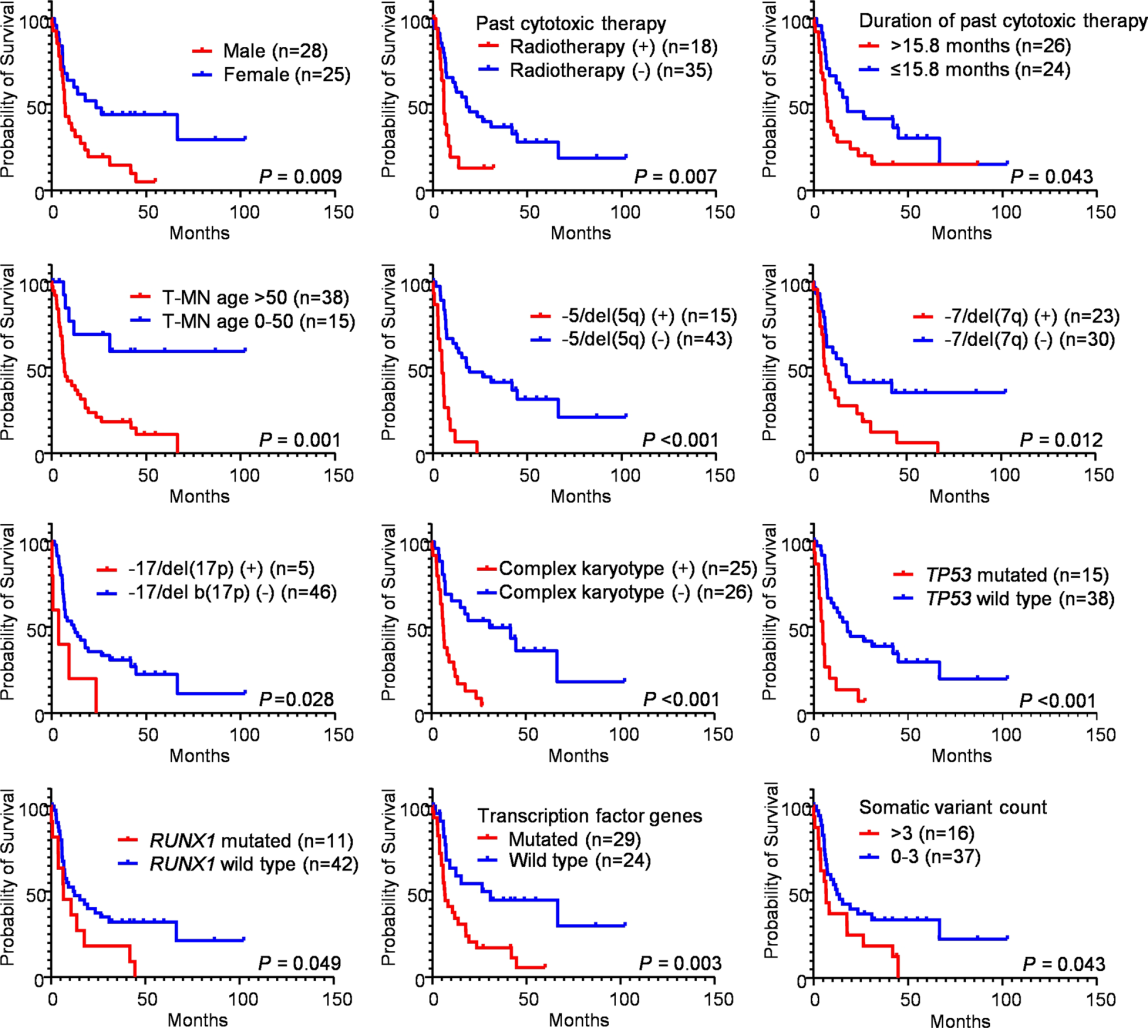
Kaplan–Meier survival curves of significant prognostic factors affecting overall survival in 53 T-MN patients

The median OS of SNUH patients was 10.6 months (range 0.2–102.4) and 9.2 months (range 0.2–102.4) when including and excluding pediatric patients, respectively (Table 1). Slightly shorter survival was observed for the patients in the SNUH group compared with those in the Singhal group. Results of univariate analysis of SNUH 53 T-MN patients showed that male sex, past radiotherapy history, past cytotoxic therapy received more than 15.8 months, T-MN diagnostic age over 50 years old, cytogenetic abnormalities such as −5/del(5q), −7/del(7q), −17/del(17p), complex karyotype, somatic *TP53* variant, somatic *RUNX1* variant, somatic variant in transcription factor genes, and somatic variant counts more than three were poor prognostic factors (Fig. 7). Kaplan–Meier plots for other variables that showed no statistical significance are shown in Additional file 5: Fig. S4.

**Fig. 8:**
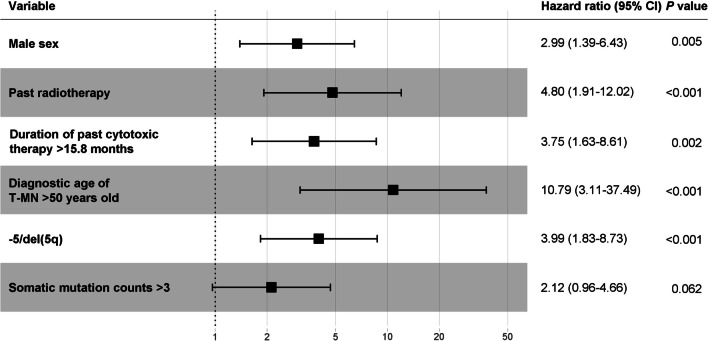
Forest plot by Multiple Cox Proportional Hazards Model of 53 T-MN patients. −7/del(7q), complex karyotype, and somatic *TP53* variant were omitted from explanatory variables due to their multicollinearity with −5/del(5q). *CI* Confidence intervals

The result of a multivariate Cox regression analysis showed that male sex (hazard ratio (HR) 2.99, 95% confidence interval (CI) 1.39–6.43, *P* = 0.005), past radiotherapy (HR 4.80, CI 1.91–12.02, *P* < 0.001), past cytotoxic therapy persisting more than 15.8 months (HR 3.75, CI 1.63–8.61, *P* = 0.002), T-MN diagnostic age over 50 years old (HR 10.79, CI 3.11–37.49, *P* < 0.001), and −5/del(5q) (HR 3.99, CI 1.83–8.73, *P* < 0.001) were independent predictors for increasing mortality (Fig. 8). −7/del(7q), complex karyotype, and somatic *TP53* variant were removed from independent variables in the multivariate Cox regression analysis because of their multicollinearity with −5/del(5q).

Except for seven pediatric patients, univariate and multivariate survival analyses were performed in 46 adult T-MN patients. The results of the univariate analysis showed that male sex, past radiotherapy history, past autologous peripheral blood stem cell transplantation (PBSCT), past cytotoxic therapy received more than 5.5 months, T-MN diagnostic age over 35 years old, cytogenetic abnormalities such as −5/del(5q), −7/del(7q), and complex karyotype, somatic *TP53* variant, and somatic variant in transcription factor genes were poor prognostic factors, whereas *PML*-*RARA* was associated with better OS (See Additional file 5: Fig. S5). Kaplan–Meier plots for other variables that showed no statistical significance are shown in Additional file 5: Fig. S6. The result of a multivariate Cox regression analysis showed that male sex (HR 4.45, CI 1.97–10.06, *P* < 0.001), past radiotherapy (HR 4.70, CI 2.06–10.72, *P* < 0.001), past cytotoxic therapy persisting more than 5.5 months (HR 3.42, CI 1.14–10.27, *P* = 0.028), and somatic *TP53* variant (HR 5.28, CI 2.26–12.31, *P* < 0.001) were independent predictors for increasing mortality (See Additional file 5: Fig. S7). −5/del(5q), −7/del(7q), complex karyotype, and somatic variant in transcription factor genes were excluded from explanatory variables in the multivariate Cox regression analysis due to their multicollinearity with somatic *TP53* variant.

## Discussion

The prognosis for T-MN is poor, and its incidence is anticipated to increase in tandem with the increase of cancer survivors. Therefore, understanding of characteristics in T-MN patients is crucial for the management of the disease. However, comprehensive research on T-MN has been discouraged due to the low incidence of T-MN. Somatic variant profiles of T-MN patients have mainly been described in Western countries. In this study, well-characterized T-MN patients in Korea were collected retrospectively for eight years, their germline predisposition and somatic variant profile were described, and the data were compared with those of other T-MN study groups.

Apart from the Singhal, SEER, and Nishiyama studies which included only adult patients, our study included seven pediatric patients with ages less than 20 years old. Even though excluding seven pediatric patients, the median age of patients in the SNUH study group was five to ten years younger than the three study groups. The fact that T-MN patients in Korea were relatively younger was in line with findings from our previous reports in which ages at diagnosis of MDS (69 years), multiple myeloma (60 years), and chronic lymphocytic leukemia (61 years) were younger than those of Caucasian [46–48].

Non-Hodgkin lymphoma, followed by breast cancer was consistently found to be the most common primary disease in the SNUH, Singhal, and US SEER studies. However, differences in the frequencies of the other primary diseases were observed between the study groups. The distribution of primary disease might be influenced by the various sample sizes and the different incidences and distributions of cancer by country.

The well-known findings of T-MN such as −5/del(5q), −7/del(7q), complex karyotype, and *TP53* somatic variant were highly observed in the SNUH group. Notably, the four variables showed an association with one another. The fact that cytogenetic abnormalities such as −5/del(5q), −7/del(7q), and complex karyotype were more highly observed in the SNUH group compared to the Singhal group might be attributed to enhanced detectability of the FISH test for cytogenetic aberrations. In the current study, in patients diagnosed with T-MN, FISH for −5/del(5q), −7/del(7q), and *KMT2A* rearrangement was routinely performed, while FISH for p53 loss was not. On the other hand, balanced cytogenetic aberrations such as rearrangement of *KMT2A*, *RUNX1*, and *MECOM* and normal karyotype were not as highly observed.

In the current study, 13.2% of patients with T-MN harbored deleterious presumed/potential germline variants in CPG. Among the seven CPG (*BRIP1*, *CEBPA*, *DDX41*, *FANCM*, *NBN*, *NF1*, and *RUNX1*), except *CEBPA* and *NF1*, the remaining five genes (71.4%) involve in DNA damage response. *BRIP1* encodes a helicase that has been associated with the maintenance of genomic stability [49]. The in vivo zebrafish model, DDX41 (dead-box helicase 41) plays a critical role in the regulation of erythropoiesis [50]. DDX41 is abundant in promoter regions, and can unwind RNA–DNA hybrids in vitro [51]. Fanconi anemia genes constitute a critical DNA damage response pathway [52], and it is suggested that FANCM prevents the lengthening of telomeres in cancer cells [53]. The RUNX family, including RUNX1, has been implicated in the regulation of DNA damage response [54], and RUNX1 stimulates the tumor suppressor p53 protein in response to DNA damage [55]. MRN complex, which is essential for DNA repair, is a sensor of double-strand breaks, and directly functions in the repair process [56]. *NBN* is crucial for early MRN recruitment [57]. Notably, germline loss-of-function mutations in *DDX41* and *RUNX1* cause myeloid neoplasm predisposition [1].

*CEBPA* gene encodes a transcription factor that plays an important role in the differentiation of granulocytes [58]. *NF1* encodes a negative regulator of RAS signaling, acts as a tumor suppressor gene [59], and the risk of T-MN is elevated in children with *NF1* germline mutations [60].

Germline predisposition was reported in 16–21% of T-MN patients in several previous studies [6–8]. In a study of 53 T-MN patients conducted in Austria by Schulz et al., 17.0% (9/53) of T-MN patients harbored germline predisposition in *BRCA1* (*n* = 2), *BRCA2* (*n* = 1), *BARD1* (*n* = 2), and *TP53* (*n* = 4) [7]. Frequent germline variants in *BRCA1*, *BRCA2*, *CHEK2*, *PALB2*, and *TP53* were observed in two out of ten T-MN patients after treatment for breast cancer [6]. Germline variants in *BRCA1*, *BRCA2*, *BARD1*, *CHEK2*, and *PALB2* genes were not detected, probably due to the low frequency of breast cancer as primary disease in the SNUH cohort. If the number of patients with breast cancer had been larger, germline variants in these genes could have been detected in the present study. Voso and colleagues reported that 13.5% (5/37) of T-MN patients harbored heterozygous variants in FA genes as germline variants [8], which were higher than those of the SNUH group (1/20, 5.0%). However, the detected FA gene variants in the Voso et al.’s study were all missense variants, which were not classified as pathogenic according to our variant strategy. The use of different strategies for filtering variants might result in the prevalence of variants.

Owing to the advance in NGS, germline predisposition in myeloid neoplasm can be easily detected, and has been of interest recently. It is estimated that 5–15% of adults and 4–13% of pediatric patients with MDS or AML harbor pathogenic germline variants in CPG [61–64]. In Korea, 7.2–11.6% of AML patients (not specified as T-AML) had germline mutations [38, 65], which is slightly less frequent than in our study group. It is speculated that germline predisposition may affect the occurrence of T-MN more strongly than de novo myeloid neoplasm.

Patients who have germline predisposition in CPG may be needed tailored therapy, and their family members can have germline predisposition [66]. To minimize graft failure and/or donor-derived leukemia, it is critical to avoid employing an affected or asymptomatic carrier member of the family as a stem cell donor. As well as T-MN being considerably predisposed to cancer susceptibility genes, germline variant testing is highly recommended for T-MN patients and their related stem cell donor candidates.

In the comparison of somatic variants between the SNUH, Singhal, and cBioPortal study groups within the 43 genes, the lowest frequency of somatic variant was observed in the SNUH group although comparing only adult patients. The younger status of T-MN patients in our group might be related to the lower frequency of somatic variants since clonal hematopoiesis increases with aging. Especially, *SRSF2* variants were observed in 15.5% and 7.3% of Singhal and cBioPortal study groups, respectively, whereas 1.8% of the SNUH group harbored *SRSF2* variants. Low depth in *SRSF2* genes in our analysis implied low quality of capture in the regions. The different prevalence and landscape of somatic variants between study groups might be affected by sample composition, sequencing method, variant filtering strategy, etc. A similar distribution of somatic variants concerning top-ranked genes (*TP53*, *RUNX1*, *DNMT3A*, *ASXL1,* and *TET2*) was observed between the SNUH and Singhal study groups. On the other hand, all three T-MN study groups harbored highly mutated *TP53* and *DNMT3A* whereas *NPM1* and *FLT3,* highly mutated in de novo AML, were less mutated [5].

Remarkably, in the SNUH group, the median somatic variant counts of the adults and pediatric patients were three and two, respectively. This result suggests that an accumulation of somatic variants is likely to occur due to clonal hematopoiesis, which is closely associated with aging, exposure to environmental stress, etc.

The genomic profiles of AML and MDS (not specified as T-MN) patients in Korea were reported in several studies. Compared to our T-MN patients, Korean AML patients harbored more somatic mutations in *DNMT3A*, *TET2*, *NPM1*, *FLT3*, *IDH2*, *KIT*, *NRAS*, *CEBPA*, and *GATA2,* but fewer somatic mutations in *TP53* and *RUNX1* [65, 67]. In Korean MDS patients, mutations in *ASXL1*, *U2AF1*, *DNMT3A*, *SRSF2*, *SF3B1*, and *TET2* were more frequently observed whereas *TP53* and *RUNX1* were less frequently mutated in comparison to our T-MN patients [46, 67].

In the current study, male sex, past radiotherapy history, longer past cytotoxic therapy (more than 15.8 months), older age (more than 50 years old), and −5/del(5q) were independent adverse prognostic factors. Our results were partly consistent with those of other studies. The US SEER study reported that sex did not affect the OS in T-MN, and a poorer prognosis was observed for T-MN patients diagnosed between 2001 and 2007 compared with those diagnosed from 2008 to 2014. However, in the SNUH study, the period when diagnosing T-MN did not affect the OS. Results of a multivariate Cox regression analysis of the Singhal study showed that T-AML, high revised International Prognostic Scoring System cytogenetic category, *TP53* variant, and *NRAS* variant were independent aggressive factors, however, treatment with disease-modifying therapy resulted in better OS. Results of our study showed that the presence of germline variants was not a prognostic factor. Consistent with our result, another study conducted in Korea also reported that the presence of germline variants showed earlier onset of AML, but did not affect OS in Korean AML patients [38].

Our study had some limitations. First, an analysis of the variant status of *PPM1D* was not performed. Mutated *PPM1D* was observed in 20% of patients with T-AML or T-MDS [68]. Second, detecting minor clones emerging at the initial stage of clonal hematopoiesis was challenging due to the low mean depth across the sequenced samples (201.57). It was more likely that variants with low VAF would be considered false positives since supporting reads were insufficient for confirmation. As a result, in the 20 patients with longitudinal samples (non-malignant and T-MN BM specimens), pre-existing CHIP with more than 10% of VAF before the occurrence of T-MN was detected in three patients, and constant or residual somatic variants at morphologic remission of T-MN was detected in two patients (See Additional file 5: Fig. S8). Finally, this study could not determine the racial differences in T-MN because of incomplete racial information or the fact that the study subjects were not of a single race.

## Conclusion

By presenting similarities and differences with the results of previous studies, our study provides valuable information about T-MN patients. The distinguishable characteristics of the 53 T-MN patients from those of other studies were younger age, higher incidence of adverse cytogenetic findings such as −5/del(5q), −7/del(7q), and complex karyotype, and different distribution of primary disease. We detected deleterious presumed/potential germline variants in CPG in 13.2% of the study population, suggesting a considerable predisposition to CPG. Stem cell transplantation is an ultimate treatment option for T-MN. Germline variant analysis in T-MN patients should be recommended, and for the patient harboring germline variant, germline variant tests should be performed in the related stem cell donor candidates. The landscape of somatic profile showed less variant frequency in our patients compared with the other study groups, while the predominance of *TP53* variant was consistent across all study groups. Sex, age, past medical history, and cytogenetic findings were useful in the prediction of prognosis in T-MN, suggesting that these markers can be considered for risk stratification.

## Supplementary Information

**Additional file 1: Table S1.** 167 genes for detection of germline variants. **Table S2.** 93 genes for detection of somatic variants. **Fig. S1.** Distribution of the statistical metrics in the analyzed samples. **Table S3.** 43 genes for comparison of somatic variants between the SNUH, Singhal and cBioPortal study groups.

**Additional file 2.** Observed somatic variants of 53 T-MN patients.

**Additional file 3: Table S4.** Somatically mutated genes in 53 T-MN patients and their gene categories. **Fig. S2.** Correlation between cytogenetic abnormalities, somatic TP53 mutation, and somatically mutated gene categories. **Fig. S3.** Landscape of somatic variants observed in the **a** SNUH, **b** Singhal and **c** cBioPortal study groups within the 43 genes.

**Additional file 4.** Distributions of somatic variants for the 37 genes observed in the SNUH, Singhal, and cBioPortal study groups within the 43 genes except for TP53.

**Additional file 5: Fig. S4.** Kaplan-Meier survival curves showing insignificant prognostic factors among 53 T-MN patients. **Fig. S5.** Kaplan-Meier survival curves of significant prognostic factors affecting overall survival in 46 adult T-MN patients. **Fig. S6.** Kaplan-Meier survival curves showing insignificant prognostic factors among 46 adult T-MN patients. **Fig. S7.** Forest plot by Multiple Cox Proportional Hazards Model of 46 adult T-MN patients. **Fig. S8.** Findings of pre-existing clonal hematopoiesis of indeterminate potential and constant or residual somatic variants in morphologic remission in T-MN.

## Data Availability

The datasets used and/or analyzed during the current study are available from the corresponding author upon reasonable request.

## Abbreviations

T-MN: Therapy-related myeloid neoplasm
T-: Therapy-related
AML: Acute myeloid leukemia
MDS: Myelodysplastic syndrome
MDS/MPN: Myelodysplastic/myeloproliferative neoplasm
CHIP: Clonal hematopoiesis of indeterminate potential
ISCN: International System for Human Cytogenetic Nomenclature
FISH: Interphase fluorescence in situ hybridization
−5/del(5q): Chromosome 5/5q deletion
−7/del(7q): Chromosome 7/7q deletion
−17/del(17p): Chromosome 17 or 17p deletion
NGS: Next-generation sequencing
VAF: Variant allele frequency
SNV: Single-nucleotide variant
InDel: Small insertions–deletions
ACMG/AMP: American College of Medical Genetics and Genomics and the Association for Molecular Pathology
AMP/ASCO/CAP: American Society of Clinical Oncology, and College of American Pathologists
PBSCT: Peripheral blood stem cell transplantation
OHSU: Oregon Health & Science University
SNUH: Seoul National University Hospital
MSKCC: Memorial Sloan Kettering Cancer Center
CPG: Cancer predisposition gene
FA: Fanconi anemia
ELN: European LeukemiaNet
IPSS-R: Revised International Prognostic Scoring System (IPSS-R)
ID: Individual identification
OS: Overall survival
