# Supplementary material for: Analysis of clinical and genomic profiles of therapy-related myeloid neoplasm in Korea
Source: Hum Genomics. 2023 Feb 23;17:13. doi: 10.1186/s40246-023-00458-8 (PMC9948421; doi:10.1186/s40246-023-00458-8)
Supplement: Supplementary file 1 — Additional file 1: Table S1. 167 genes for detection of germline variants. Table S2. 93 genes for detection of somatic variants. Fig. S1. Distribution of the statistical metrics in the analyzed samples. Table S3. 43 genes for comparison of somatic variants between the SNUH, Singhal and cBioPortal study groups. [file 40246_2023_458_MOESM1_ESM.pdf]

Supplementary Table 1. 167 genes for detection of germline variants.

ACD	*ALK*	*ANKRD26*	*AP3B1*	*APC*	*ATM*
BAP1	*BARD1*	*BLM*	*BMPR1A*	*BRAF*	*BRCA1*
BRCA2	*BRIP1*	*BTK*	*BUB1B*	*CASP10*	*CBL*
CDC73	*CDH1*	*CDK4*	*CDKN1B*	*CDKN2A*	*CDKN2B*
CEBPA	*CHEK2*	*CSF3R*	*CTC1*	*CXCR4*	*CYLD*
DDB2	*DDX41*	*DICER1*	*DIS3*	*DIS3L2*	*DKC1*
DNM2	*EGFR*	*ELANE*	*EPCAM*	*ERCC2*	*ERCC3*
ERCC4	*ERCC5*	*ETV6*	*EXT1*	*EXT2*	*FAH*
FANCA	*FANCB*	*FANCC*	*FANCD2*	*FANCE*	*FANCF*
FANCG	*FANCI*	*FANCL*	*FANCM*	*FASLG*	*FH*
FLCN	*G6PC3*	*GATA1*	*GATA2*	*GFI1*	*GPC3*
HAX1	*HNF1A*	*HRAS*	*IKZF1*	*ITK*	*KIT*
KRAS	*LAMTOR2*	*LYST*	*MAX*	*MEN1*	*MET*
MLH1	*MPL*	*MSH2*	*MSH6*	*MUTYH*	*NAF1*
NBN	*NF1*	*NF2*	*NHP2*	*NOP10*	*NRAS*
PALB2	*PAX5*	*PDGFRA*	*PHOX2B*	*PMS2*	*POLH*
POT1	*PRKAR1A*	*PRKDC*	*PRSS1*	*PTCH1*	*PTEN*
PTPN11	*RAB27A*	*RAD51*	*RAD51C*	*RAD51D*	*RAF1*
RB1	*RECQL4*	*RET*	*RMRP*	*RPL11*	*RPL27*
RPL35A	*RPL5*	*RPS10*	*RPS17*	*RPS19*	*RPS24*
RPS26	*RPS27*	*RPS7*	*RTEL1*	*RUNX1*	*SAMD9*
SAMD9L	*SBDS*	*SDHA*	*SDHAF2*	*SDHB*	*SDHC*
SDHD	*SH2B3*	*SH2D1A*	*SHOC2*	*SLC37A4*	*SLX4*
SMAD4	*SMARCA4*	*SMARCB1*	*SMARCE1*	*SOS1*	*SRP72*
STAT3	*STK11*	*STK4*	*STN1*	*SUFU*	*TAZ*
TERC	*TERT*	*TINF2*	*TMEM127*	*TP53*	*TSC1*
TSC2	*VHL*	*VPS13B*	*VPS45*	*WAS*	*WRAP53*
WRN	*WT1*	*XPA*	*XPC*	*XRCC2*	

The 33 genes in grey cells are included in both the 167 genes for detection of germline variants and the 93 genes for detection of somatic variants.

Supplementary Table 2. 93 genes for detection of somatic variants.

ABL1	*ARID1B*	*ARID2*	*ASXL1*	*ATM*	*ATRX*
B2M	*BAP1*	*BCOR*	*BCORL1*	*BIRC3*	*BRAF*
BTK	*CALR*	*CARD11*	*CBL*	*CBLB*	*CBLC*
CCND1	*CD58*	*CD79A*	*CDKN2A*	*CEBPA*	*CREBBP*
CSF3R	*CUX1*	*DDX41*	*DIS3*	*DNMT3A*	*EGFR*
EP300	*ETV6*	*EZH2*	*FBXW7*	*FLT3*	*GATA1*
GATA2	*GNA13*	*GNAS*	*HRAS*	*IDH1*	*IDH2*
IKZF1	*JAK2*	*JAK3*	*KDM6A*	*KIT*	*KMT2A*
KMT2C	*KMT2D*	*KRAS*	*LAMB4*	*LUC7L2*	*MAP2K1*
MEF2B	*MET*	*MPL*	*MYC*	*MYD88*	*NF1*
NOTCH1	*NOTCH2*	*NPM1*	*NRAS*	*PDGFRA*	*PHF6*
PTEN	*PTPN11*	*RAD21*	*RB1*	*RUNX1*	*SETBP1*
SETD2	*SF3B1*	*SH2B3*	*SMC1A*	*SMC3*	*SRP72*
SRSF2	*STAG2*	*STAT3*	*TERT*	*TET2*	*TNFAIP3*
TNFRSF14	*TP53*	*TRAF3*	*U2AF1*	*U2AF2*	*WT1*
XPO1	*ZAP70*	*ZRSR2*			

The 33 genes in grey cells are included in both the 167 genes for detection of germline variants and the 93 genes for detection of somatic variants.

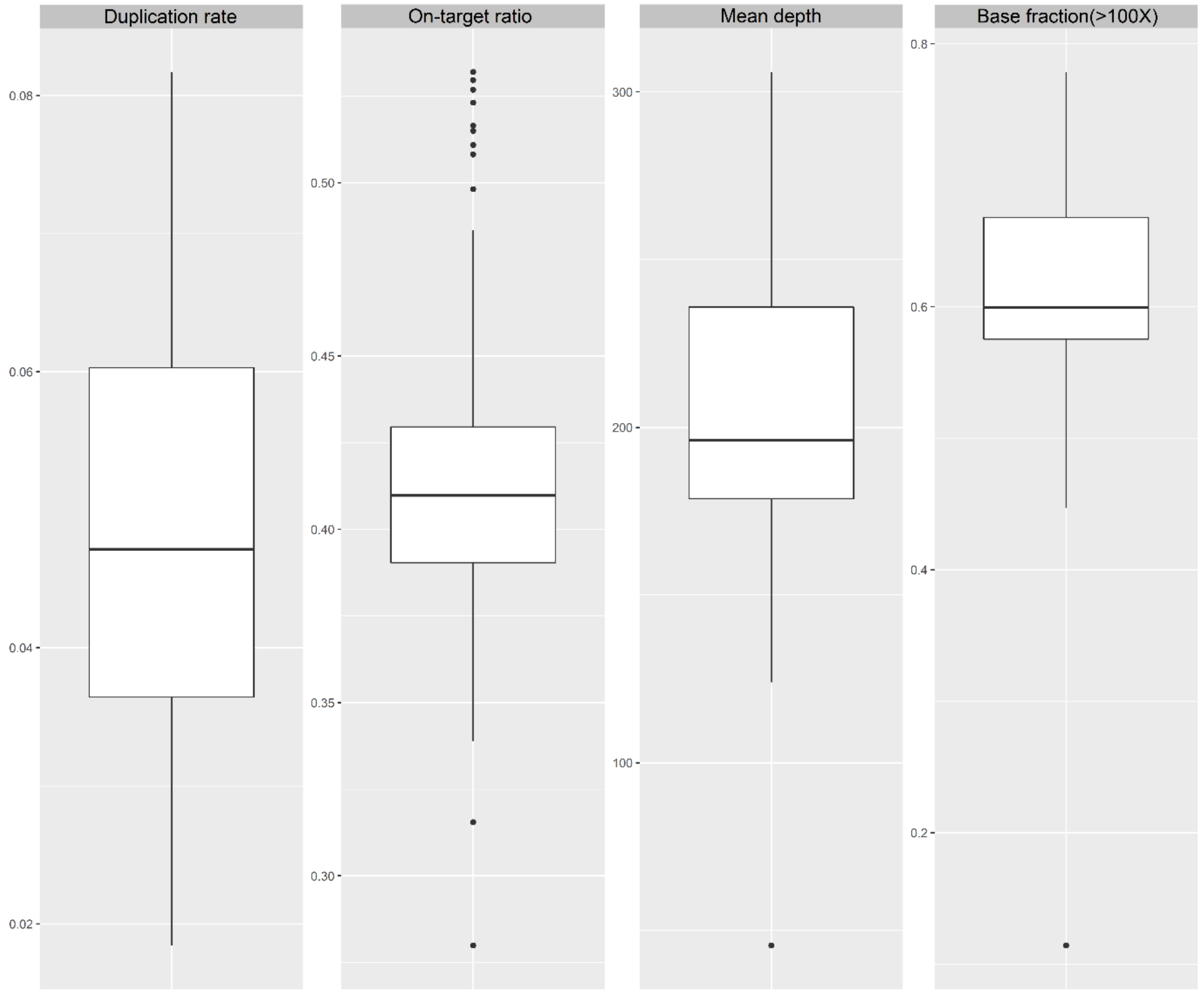

Supplementary Figure 1. Distribution of the statistical metrics in the analyzed samples.

This plot illustrates the distribution of the statistical metrics calculated using whole samples included in the study (n=77): Duplication rate, On-target ratio, Mean depth, and Base fraction (>100X). The definition of each statistical metric variable is mentioned respectively as follows: “Duplicate rate” refers to the fraction of the mapped sequence that is marked as duplicate; “On-target ratio” refers to the fraction of PF_BASES_ALIGNED (i.e., the number of unique bases that are aligned to the reference genome with mapping scores >0) located on or near a baited region; “Mean depth” refers to the average depth of a targeted region; “Base fraction (>100X)” refers to the fraction of all target bases achieving 100X or greater coverage. The mean depth of whole samples (n=77) was 201.57.

Supplementary Table 3. 43 genes for comparison of somatic variants between the SNUH, Singhal, and cBioPortal study groups.

ASXL1	*BAP1*	*BCOR*	*BCORL1*	*BRAF*	*CBL*
CDKN2A	*DDX41*	*DNMT3A*	*EGFR*	*EZH2*	*FLT3*
GATA2	*GNAS*	*IDH1*	*IDH2*	*JAK2*	*KIT*
KMT2A	*KRAS*	*LAMB4*	*MET*	*MPL*	*MYD88*
NF1	*NOTCH1*	*NPM1*	*NRAS*	*PDGFRA*	*PTEN*
PTPN11	*RUNX1*	*SETBP1*	*SF3B1*	*SRP72*	*SRSF2*
STAG2	*TERT*	*TET2*	*TP53*	*U2AF1*	*WT1*
ZRSR2					
