# Supplementary material for: Analysis of clinical and genomic profiles of therapy-related myeloid neoplasm in Korea
Source: Hum Genomics. 2023 Feb 23;17:13. doi: 10.1186/s40246-023-00458-8 (PMC9948421; doi:10.1186/s40246-023-00458-8)
Supplement: Supplementary file 3 — Additional file 3: Table S4. Somatically mutated genes in 53 T-MN patients and their gene categories. Fig. S2. Correlation between cytogenetic abnormalities, somatic TP53 mutation, and somatically mutated gene categories. Fig. S3. Landscape of somatic variants observed in the a SNUH, b Singhal and c cBioPortal study groups within the 43 genes. [file 40246_2023_458_MOESM3_ESM.pdf]

Supplementary Table 4. Somatically mutated genes in 53 T-MN patients and their gene categories.

**Gene categories**	**Included genes**
Transcription factor	*BCOR, BCOR1, CEBPA, CUX, ETV6, MEF2B, PHF6, RUNX1, TP53, WT1*
DNA repair/Cell cycle	*ATM, CREBBP, DDX41, EP300, RB1*
Chromatin modification	*ARID1B, ARID2, ASXL1, ATRX, DNMT3A, EZH2, IDH1, IDH2, KMT2C, KMT2D, SETD2, TET2*
RAS pathway	*BRAF, CBL, CBLC, KRAS, NF1, NRAS, PTPN11*
Receptor/kinase	*ABL1, EGFR, FBXW7, FLT3, KIT, NOTCH2*
Splicing factor	*SF3B1, SRSF2, ZRSR2*
Cohesin complex	*SMC1A, SMC3*
Miscellaneous	*BAP1, DIS3, GNAS, LAMB4, SETBP1, SRP72, TERT, TNFAIP3, TRAF3, XPO1*

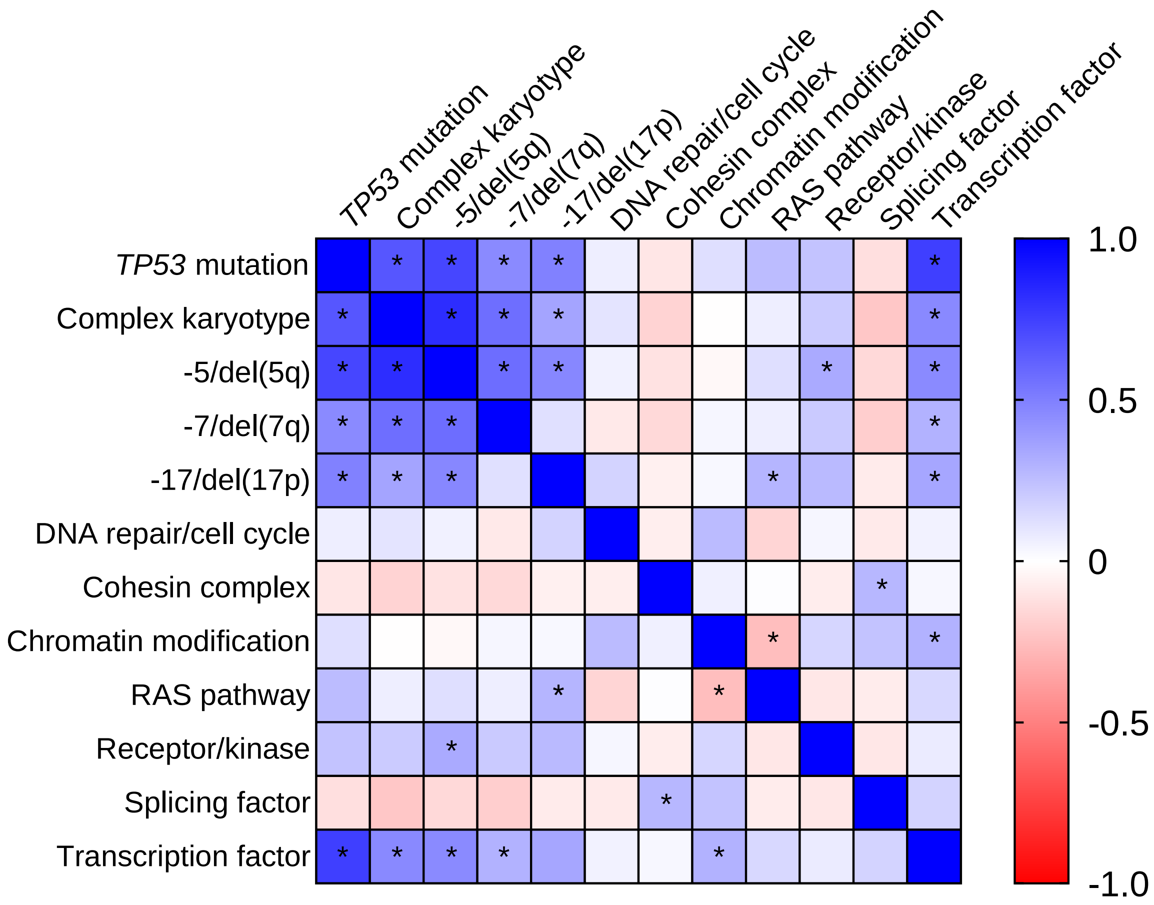

Supplementary Figure 2. Correlation between cytogenetic abnormalities, somatic *TP53* mutation, and somatically mutated gene categories.

*P value <0.05.

(a)

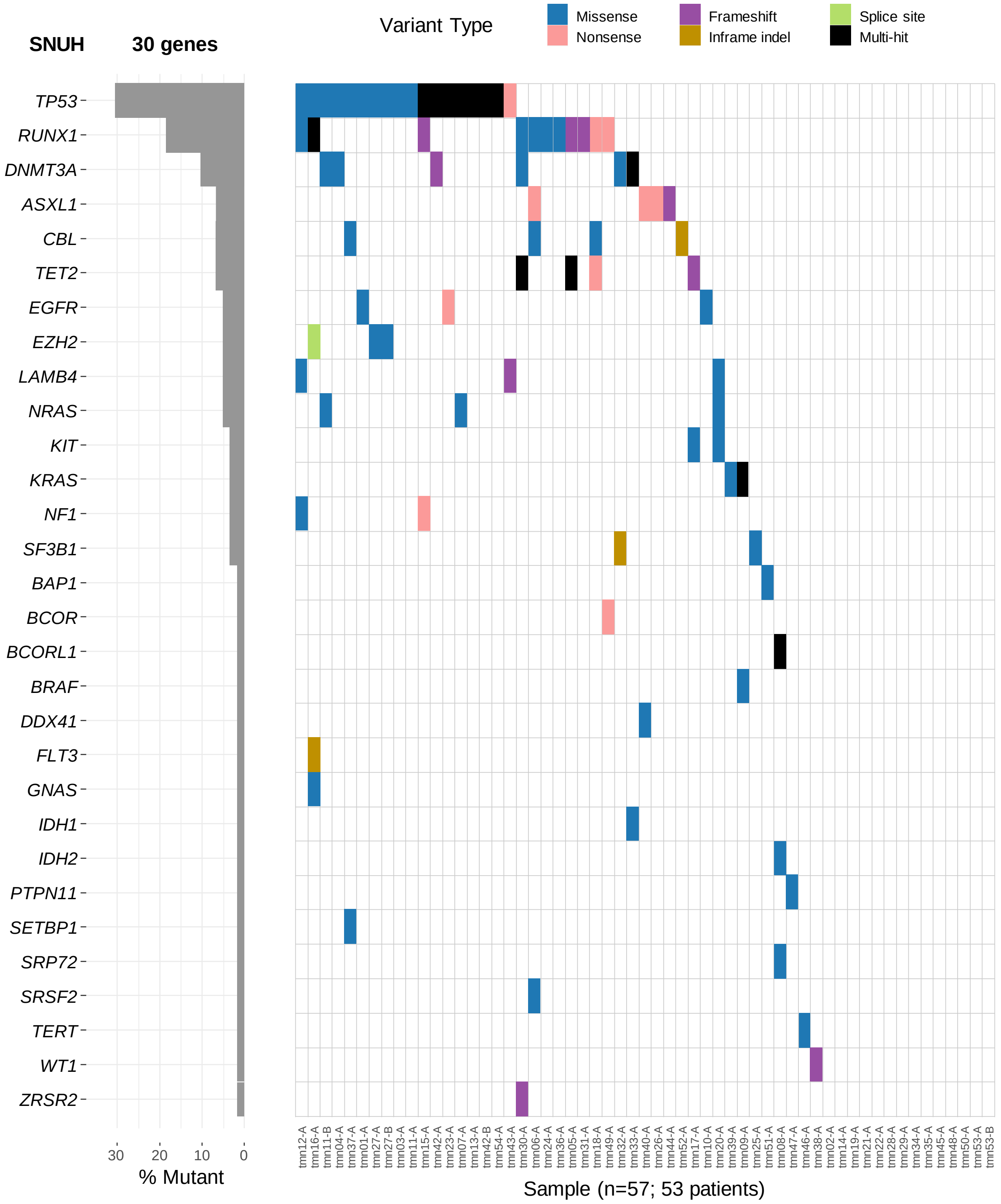

Supplementary Figure 3. Landscape of somatic variants observed in the (a) SNUH, (b) Singhal, and (c) cBioPortal study groups within the 43 genes.

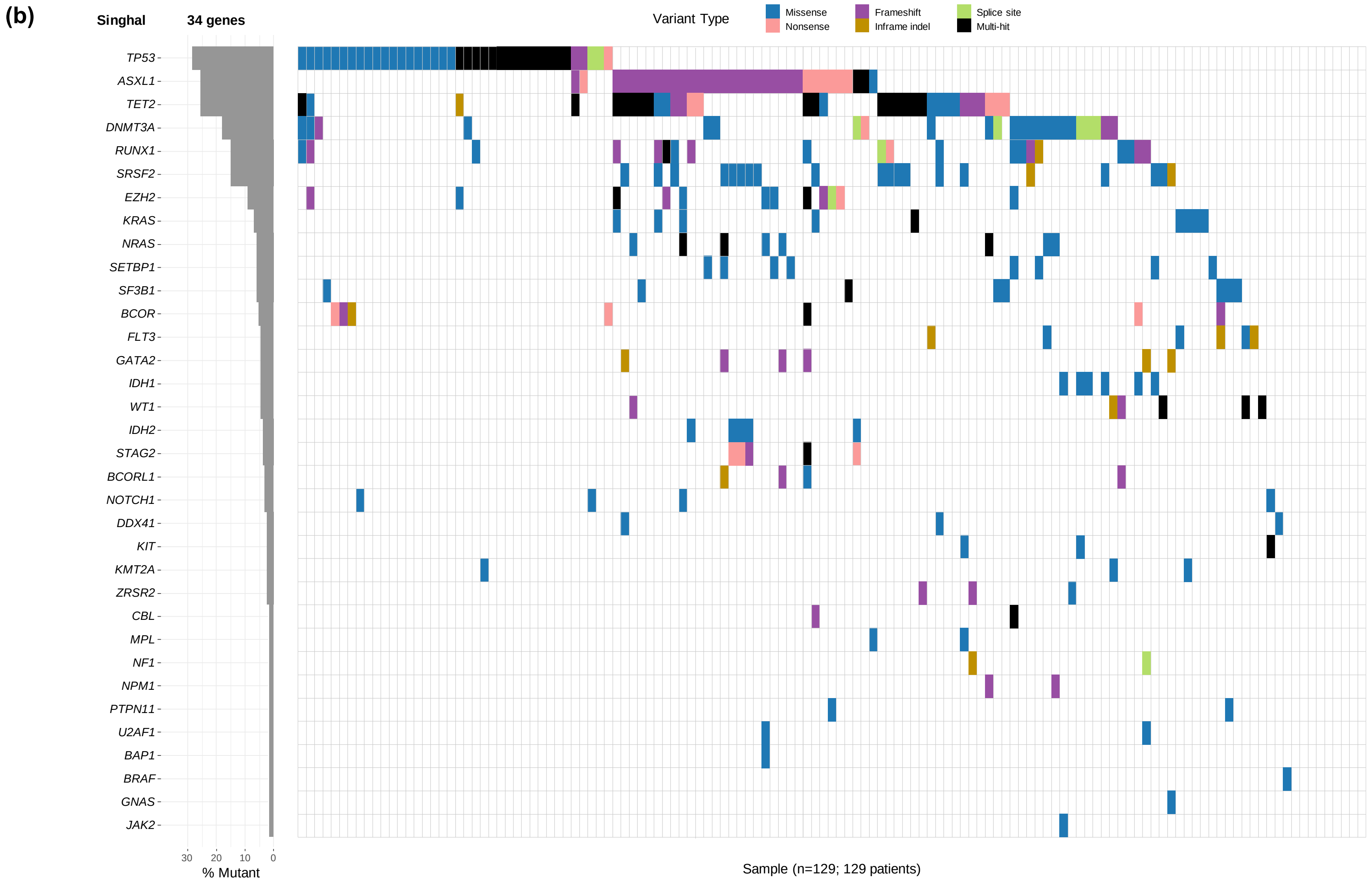

Supplementary Figure 3. Continued.

(c)

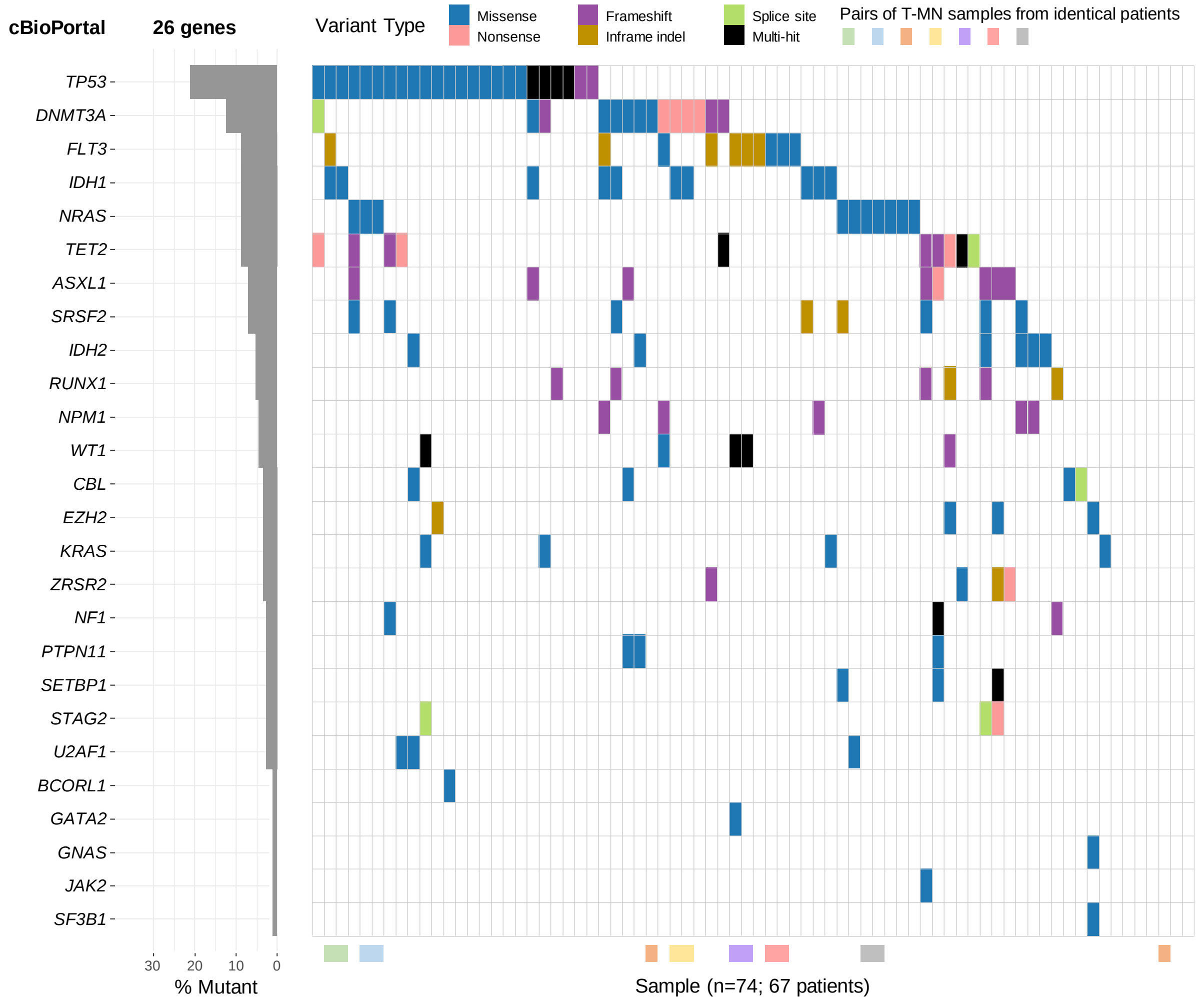

Supplementary Figure 3. Continued.
