# Supplementary material for: Analysis of clinical and genomic profiles of therapy-related myeloid neoplasm in Korea
Source: Hum Genomics. 2023 Feb 23;17:13. doi: 10.1186/s40246-023-00458-8 (PMC9948421; doi:10.1186/s40246-023-00458-8)
Supplement: Supplementary file 4 — Additional file 4. Distributions of somatic variants for the 37 genes observed in the SNUH, Singhal, and cBioPortal study groups within the 43 genes except for TP53. [file 40246_2023_458_MOESM4_ESM.pdf]

Distributions of somatic variants for the 37 genes observed in the SNUH, Singhal, and cBioPortal study groups within the 43 genes except for *TP53*.

ASXL1

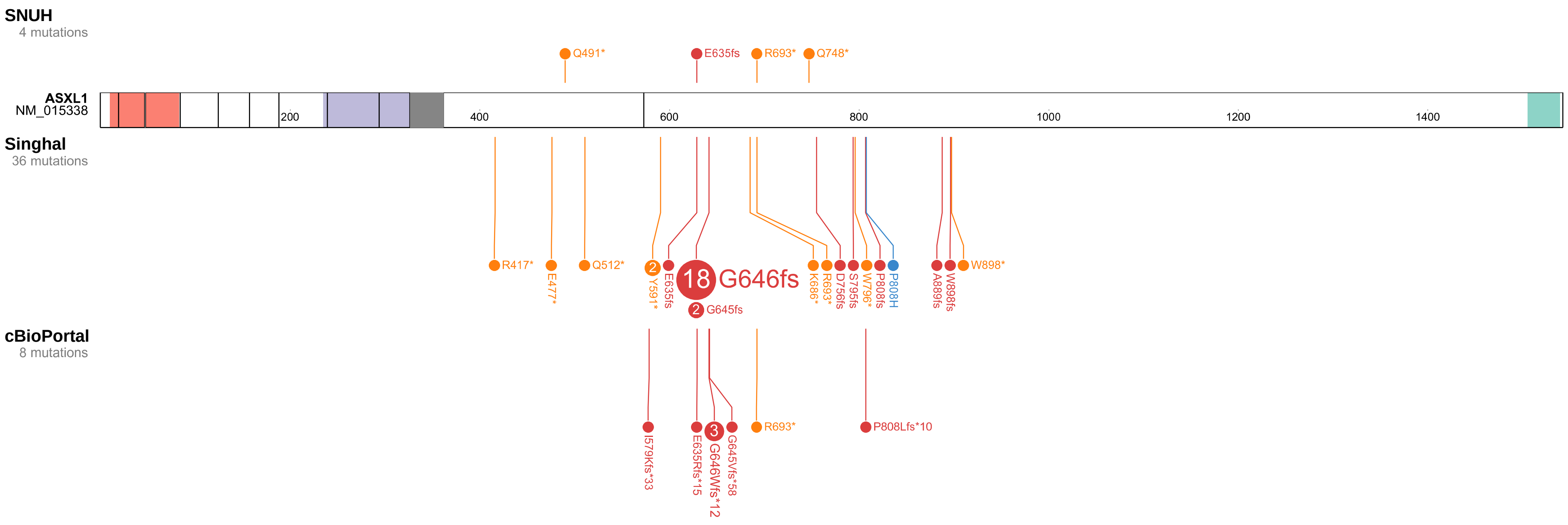
SNUH
4 mutations
Q491*
E635fs
R693*
Q748*
ASXL1
NM_015338
200
400
600
800
1000
1200
1400
Singhal
36 mutations
R417*
E477*
Q512*
2
Y591*
E635fs
18
G646fs
2
G645fs
K686*
R693*
D756fs
S795fs
W796*
P808fs
P808H
A889fs
W898fs
W898*
cBioPortal
8 mutations
I579Kfs*33
E635Rfs*15
3
G646Wfs*12
G645Vfs*58
R693*
P808Lfs*10
HARE-HTH HB1, ASXL, restriction endonuclease HTH domain
ASXH Asx homology domain
PHD_3 PHD domain of transcriptional enhancer, Asx
Nonsense, n=13
Frameshift, n=34
Missense, n=1

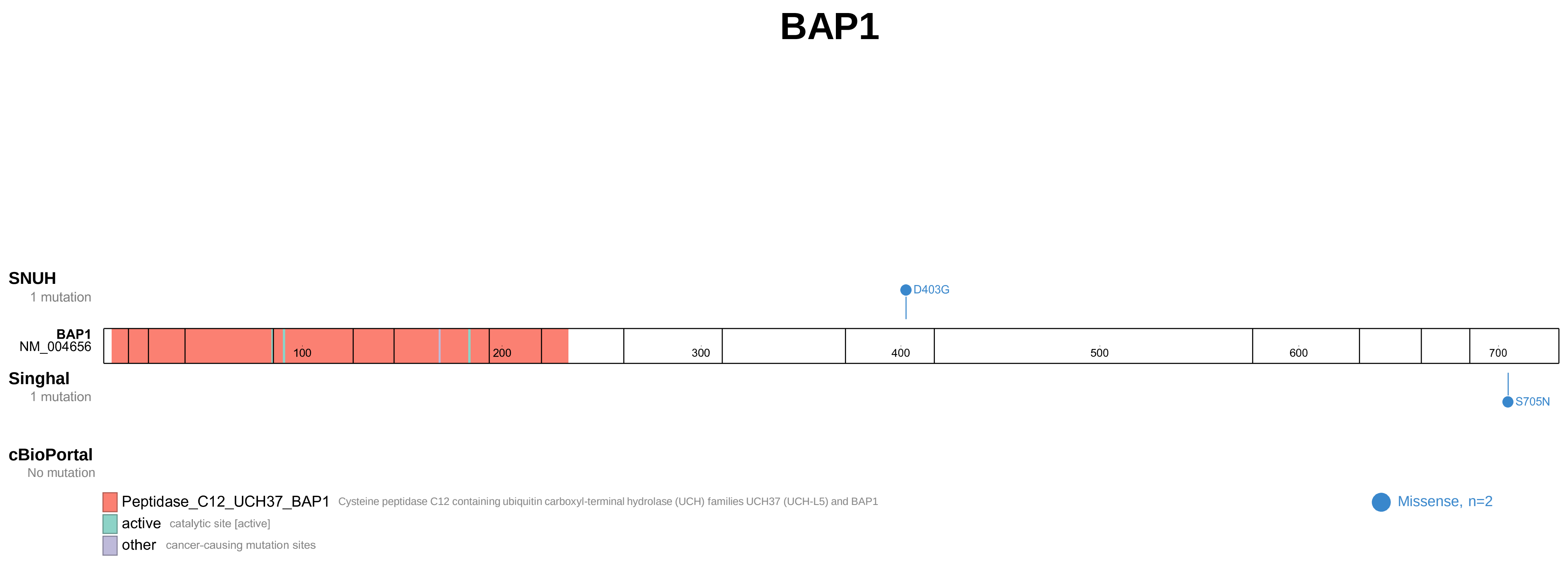
BAP1
SNUH
1 mutation
D403G
BAP1
NM_004656
100
200
300
400
500
600
700
Singhal
1 mutation
S705N
cBioPortal
No mutation
Peptidase_C12_UCH37_BAP1 Cysteine peptidase C12 containing ubiquitin carboxyl-terminal hydrolase (UCH) families UCH37 (UCH-L5) and BAP1
active catalytic site [active]
other cancer-causing mutation sites
Missense, n=2

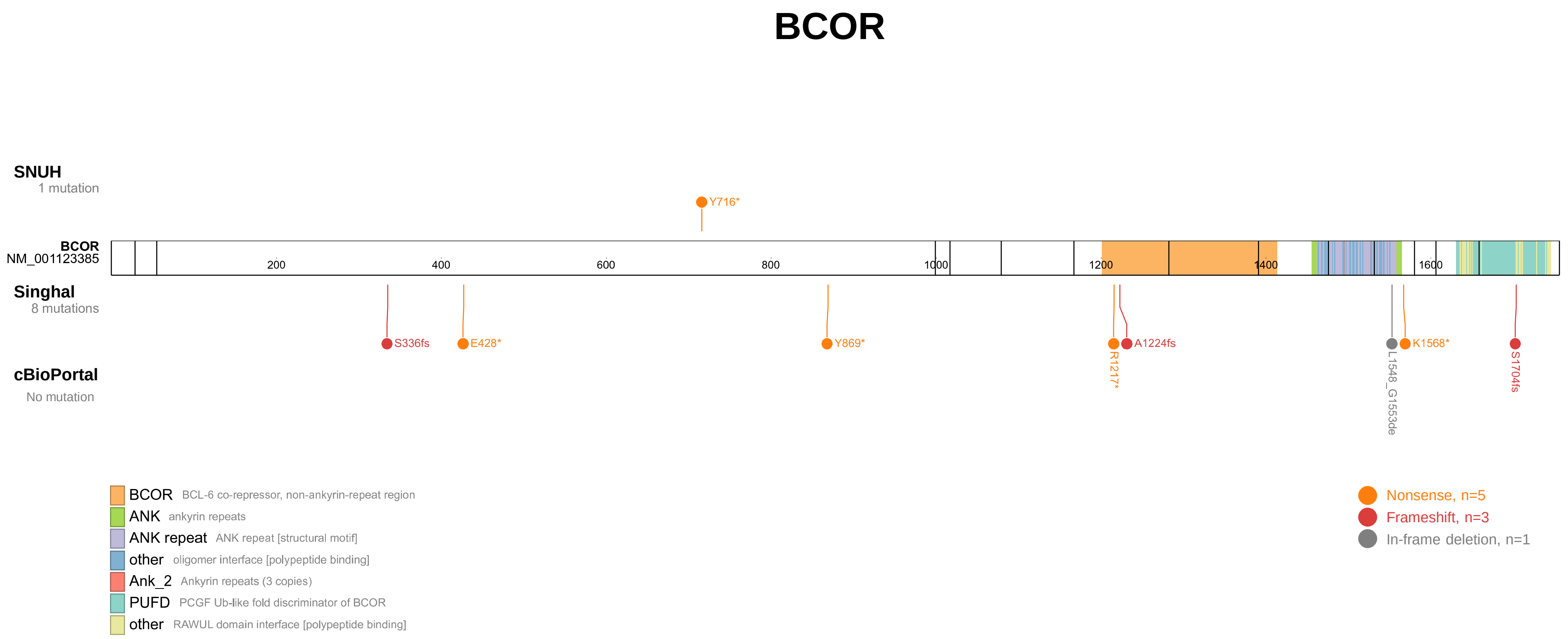
BCOR
SNUH
1 mutation
Y716*
BCOR
NM_001123385
200
400
600
800
1000
1200
1400
1600
Singhal
8 mutations
S336fs
E428*
Y869*
R1217*
A1224fs
L1548_G1553del
K1568*
S1704fs
cBioPortal
No mutation
BCOR BCL-6 co-repressor, non-ankyrin-repeat region
ANK ankyrin repeats
ANK repeat ANK repeat [structural motif]
other oligomer interface [polypeptide binding]
Ank_2 Ankyrin repeats (3 copies)
PUFD PCGF Ub-like fold discriminator of BCOR
other RAWUL domain interface [polypeptide binding]
Nonsense, n=5
Frameshift, n=3
In-frame deletion, n=1

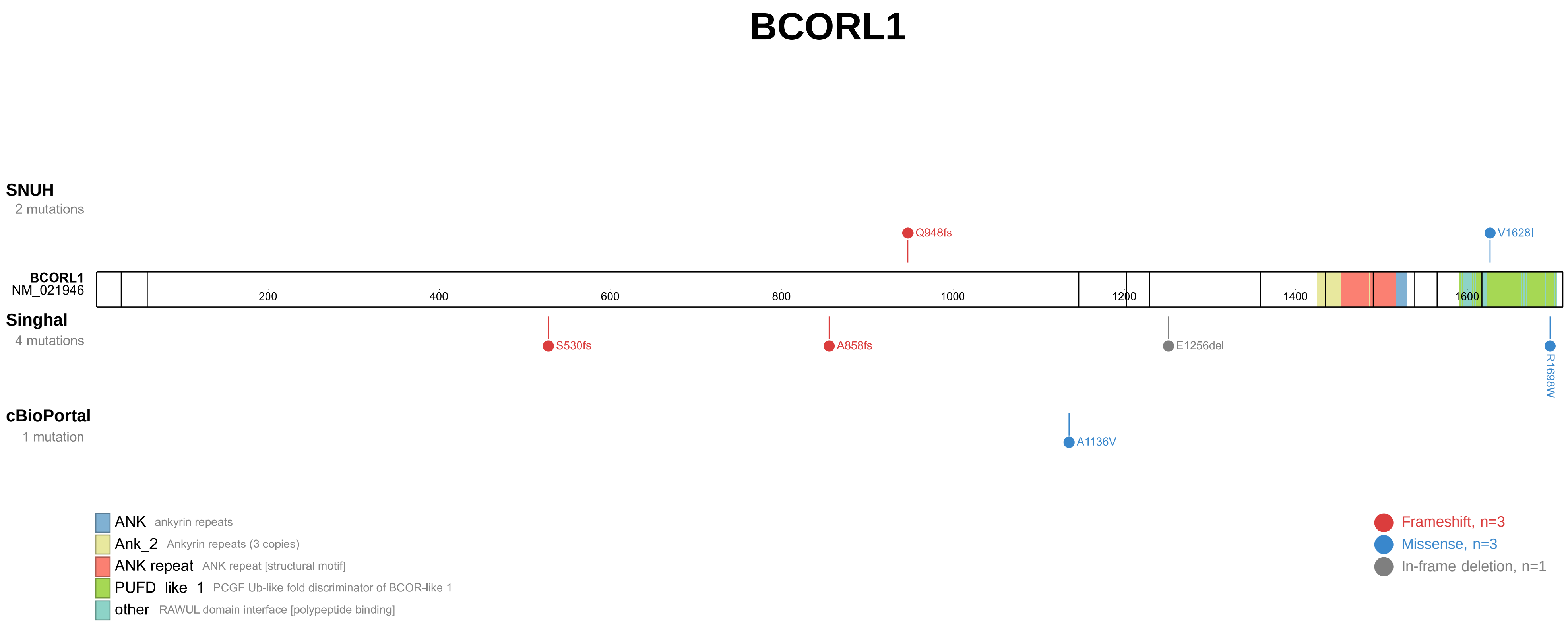
BCORL1
SNUH
2 mutations
Q948fs
V1628I
BCORL1
NM_021946
200
400
600
800
1000
1200
1400
1600
Singhal
4 mutations
S530fs
A858fs
E1256del
R1698W
cBioPortal
1 mutation
A1136V
ANK ankyrin repeats
Ank_2 Ankyrin repeats (3 copies)
ANK repeat ANK repeat [structural motif]
PUFD_like_1 PCGF Ub-like fold discriminator of BCOR-like 1
other RAWUL domain interface [polypeptide binding]
Frameshift, n=3
Missense, n=3
In-frame deletion, n=1

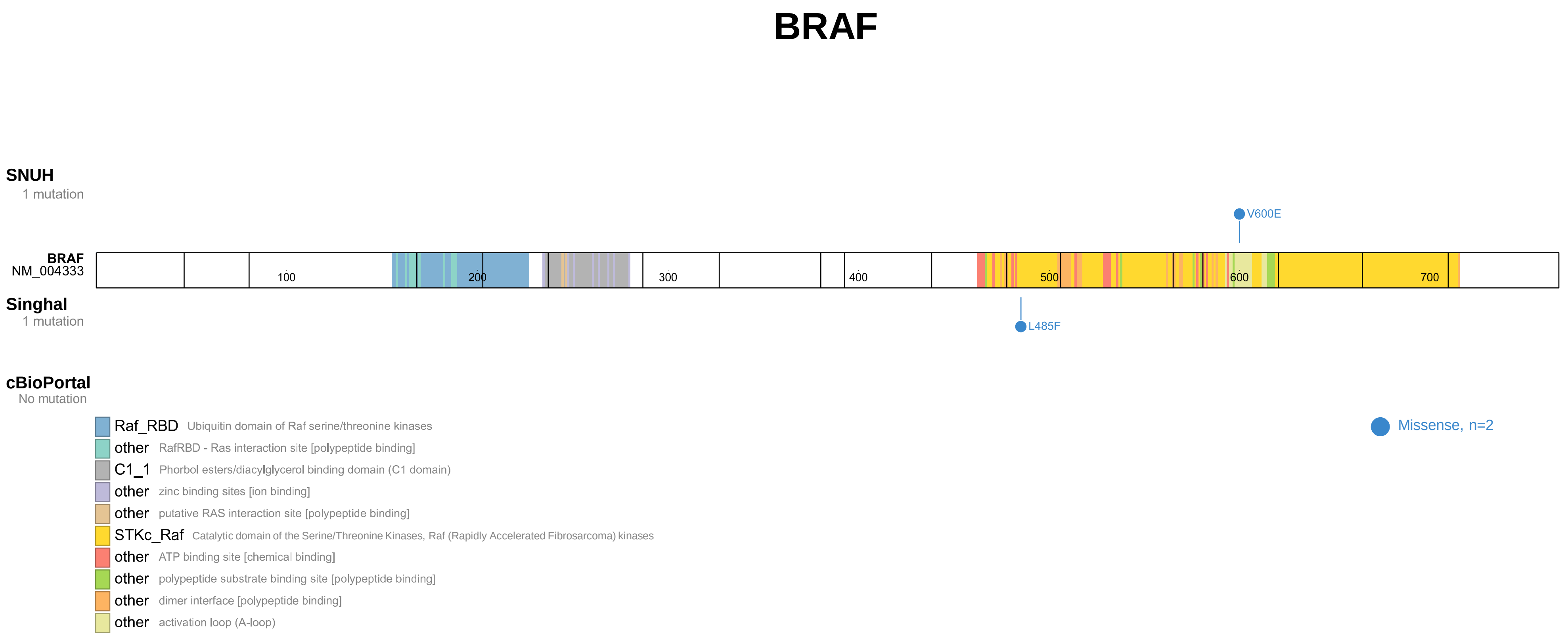

BRAF
SNUH
1 mutation
V600E
BRAF
NM_004333
100
200
300
400
500
600
700
Singhal
1 mutation
L485F
cBioPortal
No mutation
Raf_RBD Ubiquitin domain of Raf serine/threonine kinases
other RafRBD - Ras interaction site [polypeptide binding]
C1_1 Phorbol esters/diacylglycerol binding domain (C1 domain)
other zinc binding sites [ion binding]
other putative RAS interaction site [polypeptide binding]
STKc_Raf Catalytic domain of the Serine/Threonine Kinases, Raf (Rapidly Accelerated Fibrosarcoma) kinases
other ATP binding site [chemical binding]
other polypeptide substrate binding site [polypeptide binding]
other dimer interface [polypeptide binding]
other activation loop (A-loop)
Missense, n=2

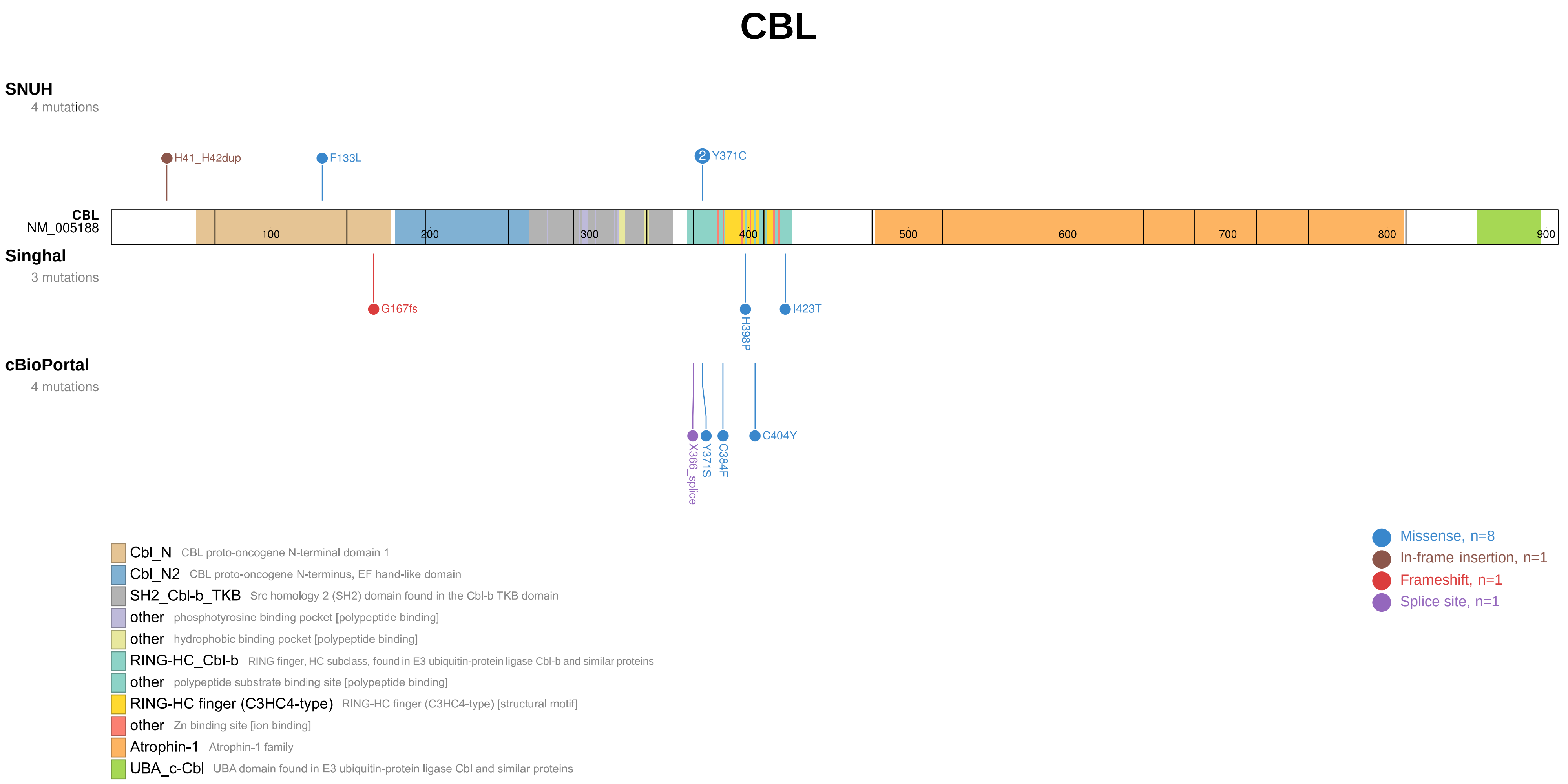

CBL
SNUH
4 mutations
H41_H42dup
F133L
2 Y371C
CBL
NM_005188
100
200
300
400
500
600
700
800
900
Singhal
3 mutations
G167fs
H398P
I423T
cBioPortal
4 mutations
X366_splice
Y371S
C384F
C404Y
Cbl_N CBL proto-oncogene N-terminal domain 1
Cbl_N2 CBL proto-oncogene N-terminus, EF hand-like domain
SH2_Cbl-b_TKB Src homology 2 (SH2) domain found in the Cbl-b TKB domain
other phosphotyrosine binding pocket [polypeptide binding]
other hydrophobic binding pocket [polypeptide binding]
RING-HC_Cbl-b RING finger, HC subclass, found in E3 ubiquitin-protein ligase Cbl-b and similar proteins
other polypeptide substrate binding site [polypeptide binding]
RING-HC finger (C3HC4-type) RING-HC finger (C3HC4-type) [structural motif]
other Zn binding site [ion binding]
Atrophin-1 Atrophin-1 family
UBA_c-Cbl UBA domain found in E3 ubiquitin-protein ligase Cbl and similar proteins
Missense, n=8
In-frame insertion, n=1
Frameshift, n=1
Splice site, n=1

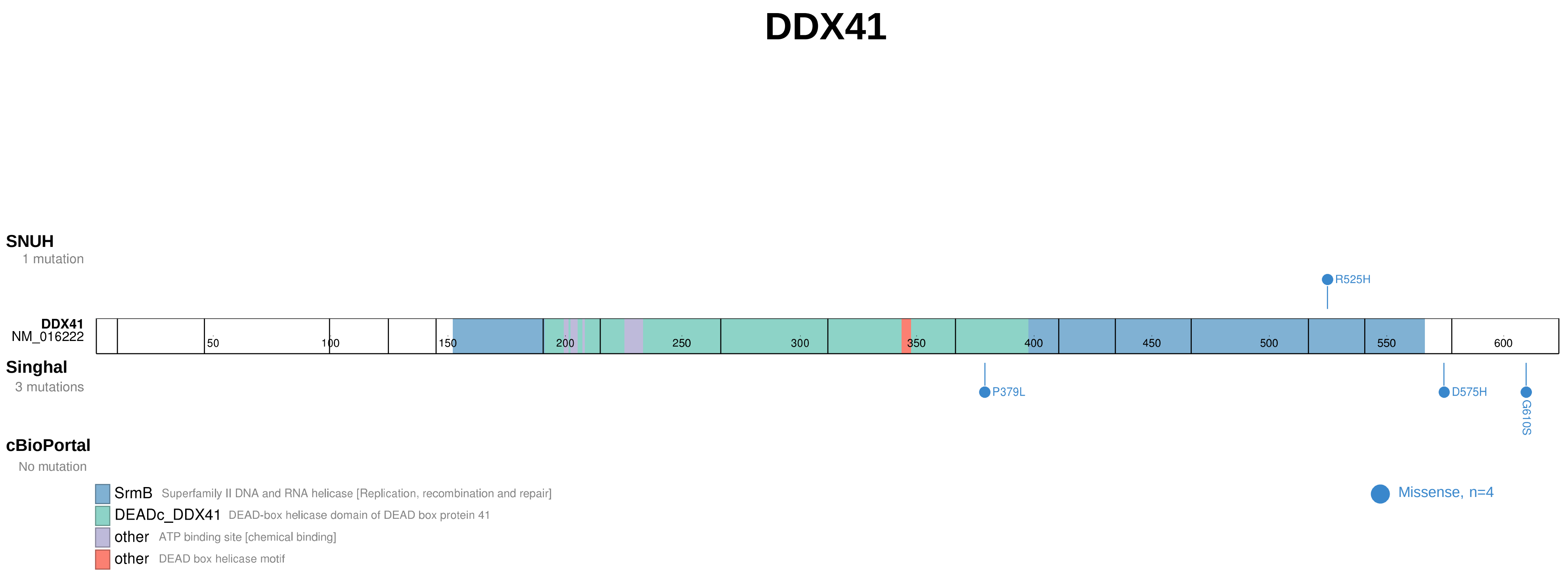
DDX41
SNUH
1 mutation
R525H
DDX41
NM_016222
50
100
150
200
250
300
350
400
450
500
550
600
Singhal
3 mutations
P379L
D575H
G610S
cBioPortal
No mutation
SrmB Superfamily II DNA and RNA helicase [Replication, recombination and repair]
DEADc_DDX41 DEAD-box helicase domain of DEAD box protein 41
other ATP binding site [chemical binding]
other DEAD box helicase motif
Missense, n=4

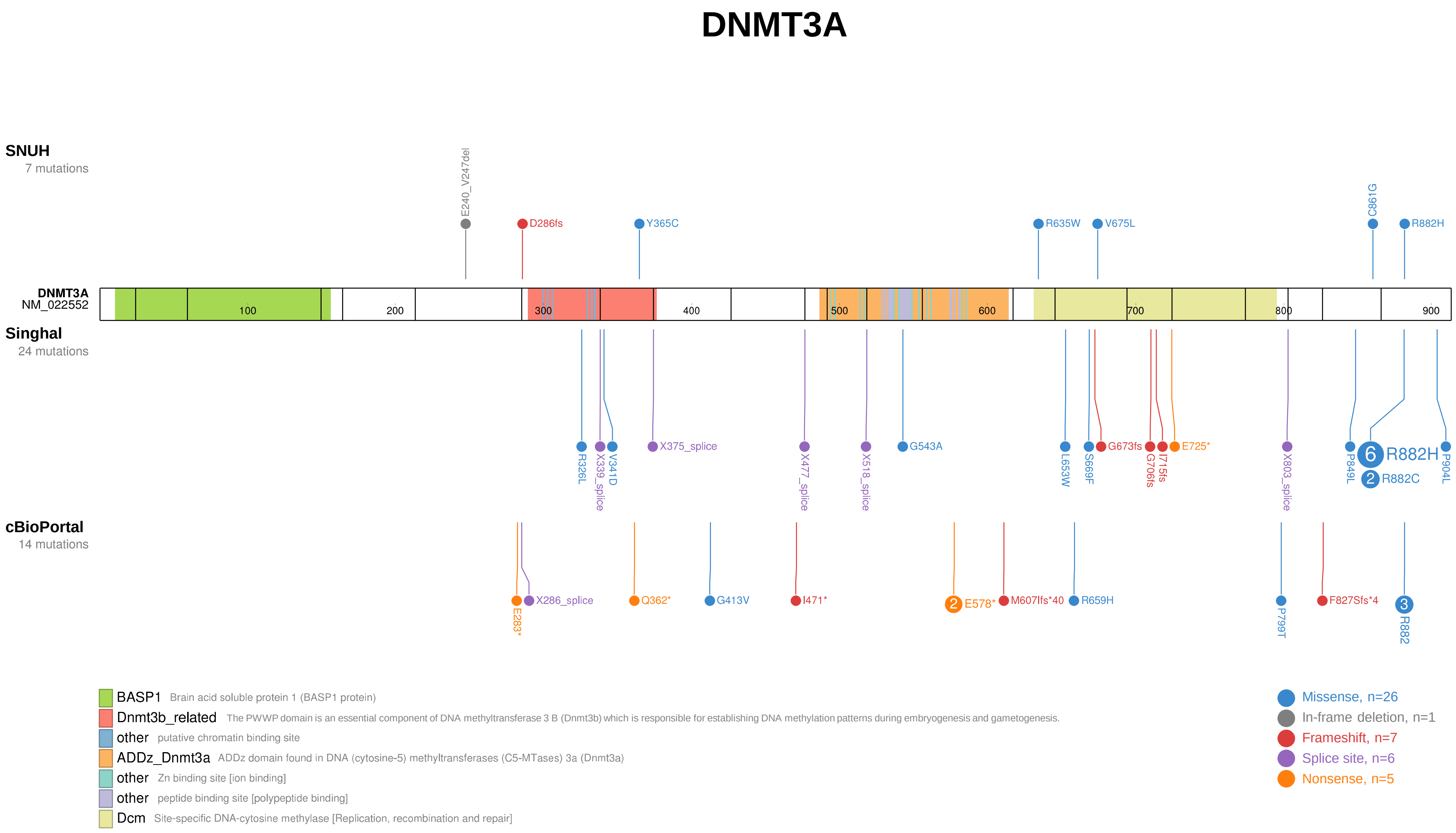
DNMT3A
SNUH
7 mutations
E240_V247del
D286fs
Y365C
R635W
V675L
C861G
R882H
DNMT3A
NM_022552
100
200
300
400
500
600
700
800
900
Singhal
24 mutations
R326L
X339_splice
V341D
X375_splice
X477_splice
X518_splice
G543A
L653W
S669F
G673fs
G706fs
I715fs
E725*
X803_splice
P849L
6 R882H
2 R882C
P904L
cBioPortal
14 mutations
E283*
X286_splice
Q362*
G413V
I471*
2 E578*
M607Ifs*40
R659H
P799T
F827Sfs*4
3 R882
BASP1 Brain acid soluble protein 1 (BASP1 protein)
Dnmt3b_related The PWWP domain is an essential component of DNA methyltransferase 3 B (Dnmt3b) which is responsible for establishing DNA methylation patterns during embryogenesis and gametogenesis.
other putative chromatin binding site
ADDz_Dnmt3a ADDz domain found in DNA (cytosine-5) methyltransferases (C5-MTases) 3a (Dnmt3a)
other Zn binding site [ion binding]
other peptide binding site [polypeptide binding]
Dcm Site-specific DNA-cytosine methylase [Replication, recombination and repair]
Missense, n=26
In-frame deletion, n=1
Frameshift, n=7
Splice site, n=6
Nonsense, n=5

EGFR

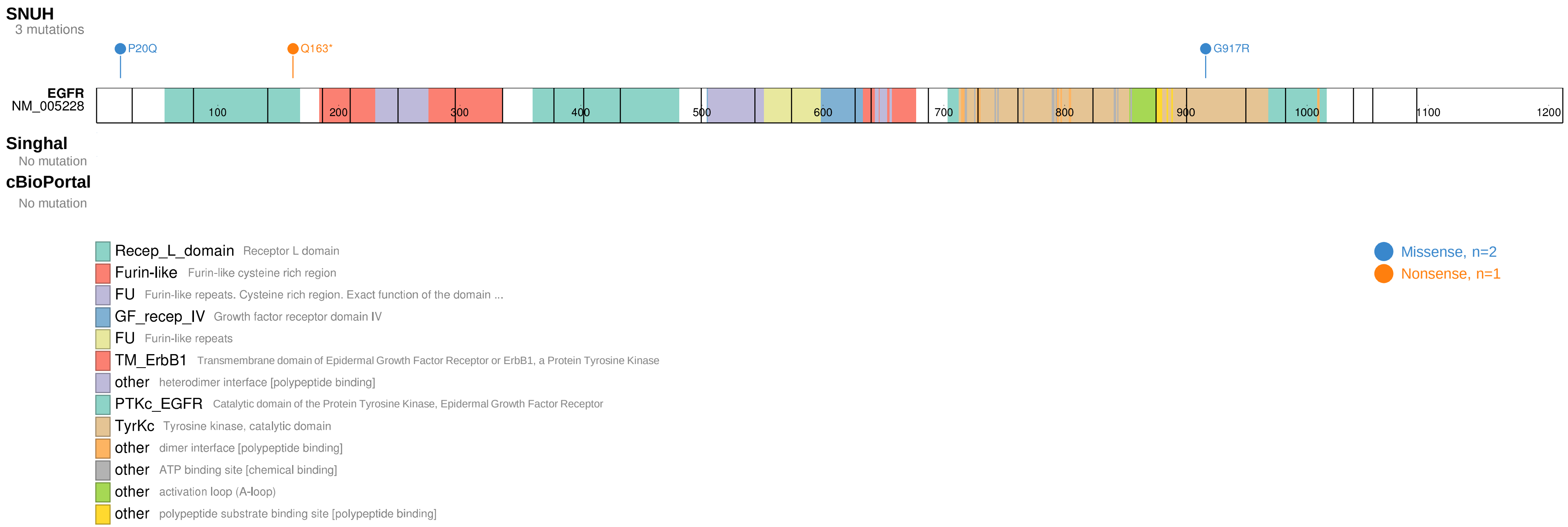
SNUH
3 mutations
P20Q
Q163*
G917R
EGFR
NM_005228
100
200
300
400
500
600
700
800
900
1000
1100
1200
Singhal
No mutation
cBioPortal
No mutation
Recep_L_domain Receptor L domain
Furin-like Furin-like cysteine rich region
FU Furin-like repeats. Cysteine rich region. Exact function of the domain ...
GF_recep_IV Growth factor receptor domain IV
FU Furin-like repeats
TM_ErbB1 Transmembrane domain of Epidermal Growth Factor Receptor or ErbB1, a Protein Tyrosine Kinase
other heterodimer interface [polypeptide binding]
PTKc_EGFR Catalytic domain of the Protein Tyrosine Kinase, Epidermal Growth Factor Receptor
TyrKc Tyrosine kinase, catalytic domain
other dimer interface [polypeptide binding]
other ATP binding site [chemical binding]
other activation loop (A-loop)
other polypeptide substrate binding site [polypeptide binding]
Missense, n=2
Nonsense, n=1

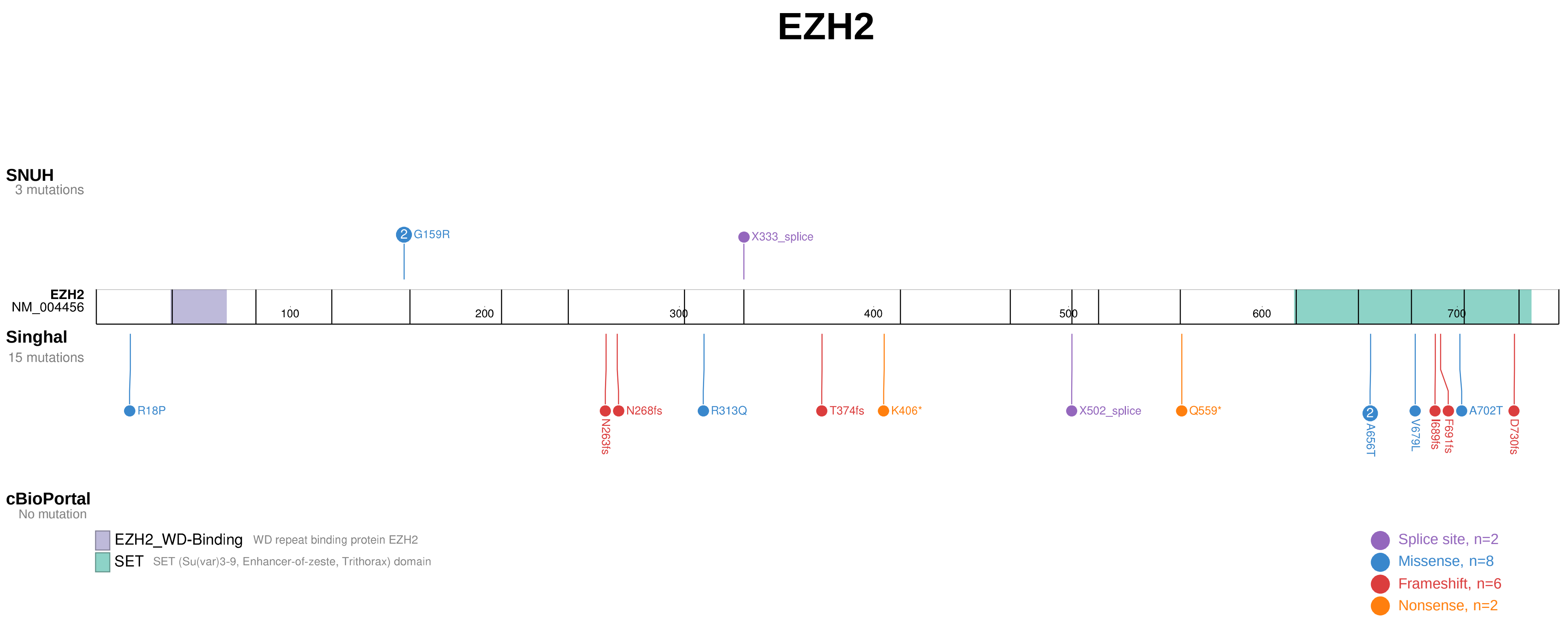
EZH2
SNUH
3 mutations
2 G159R
X333_splice
EZH2
NM_004456
100
200
300
400
500
600
700
Singhal
15 mutations
R18P
N263fs
N268fs
R313Q
T374fs
K406*
X502_splice
Q559*
2 A656T
V679L
I689fs
F691fs
A702T
D730fs
cBioPortal
No mutation
EZH2_WD-Binding WD repeat binding protein EZH2
SET SET (Su(var)3-9, Enhancer-of-zeste, Trithorax) domain
Splice site, n=2
Missense, n=8
Frameshift, n=6
Nonsense, n=2

FLT3

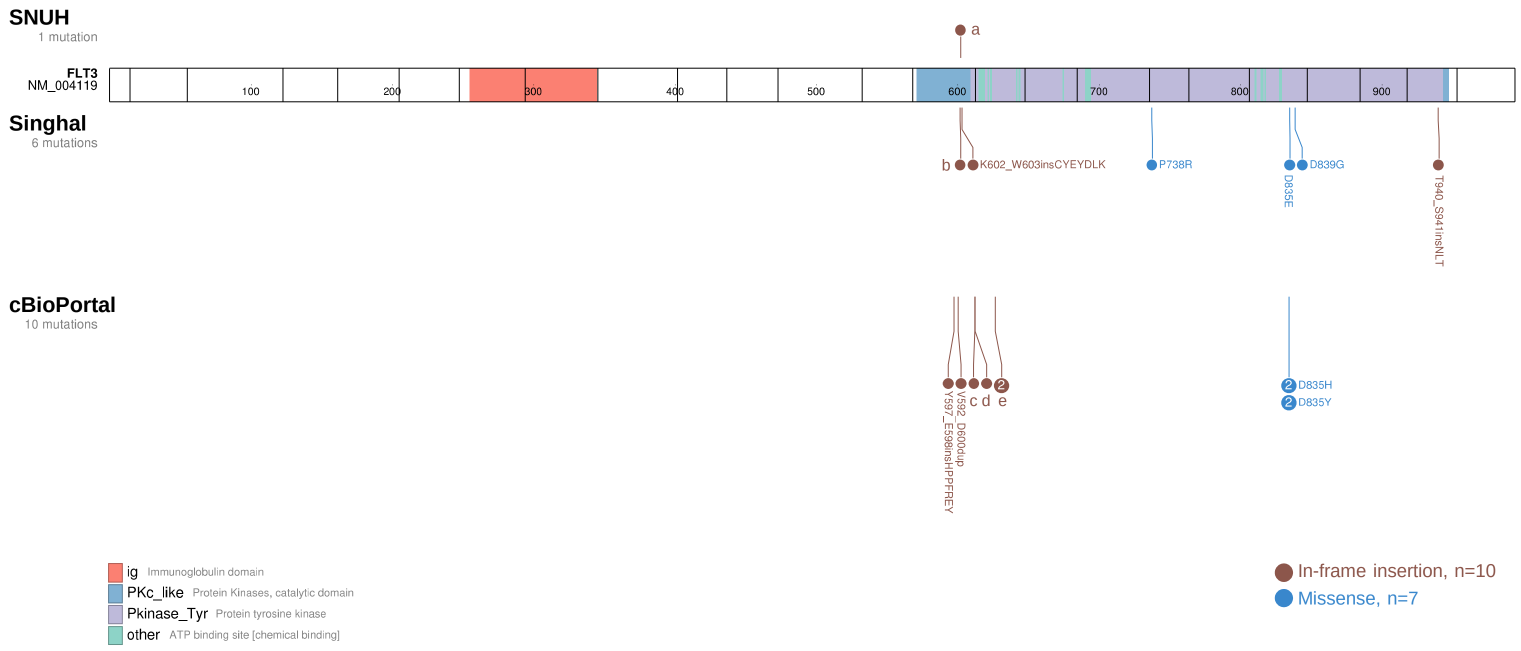

a K602_W603insVTGSSDNEYFYVDFREYEYDLK
b L601_K602insNVDFREYEYDL
c E611_F612insLTREYEYDLKWEFPRENLE
d F612_G613insADNEYFYVDFREYEYDLKWEFPRENLEF
e A627_T628insETGSSDNEYFYVDFREYEYDLKWEFPRENLEFGKNGMCQMFLQHFFSIGKSLKCTYSPFVFAGKVLGSGAFGKVMNA

GATA2

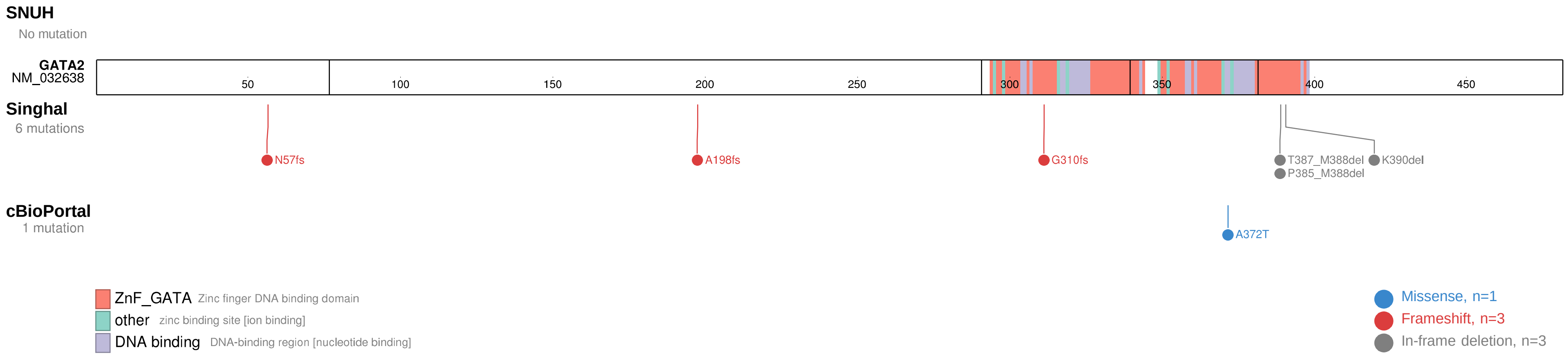

SNUH
No mutation
GATA2
NM_032638
50
100
150
200
250
300
350
400
450
Singhal
6 mutations
N57fs
A198fs
G310fs
T387_M388del
P385_M388del
K390del
cBioPortal
1 mutation
A372T
ZnF_GATA Zinc finger DNA binding domain
other zinc binding site [ion binding]
DNA binding DNA-binding region [nucleotide binding]
Missense, n=1
Frameshift, n=3
In-frame deletion, n=3

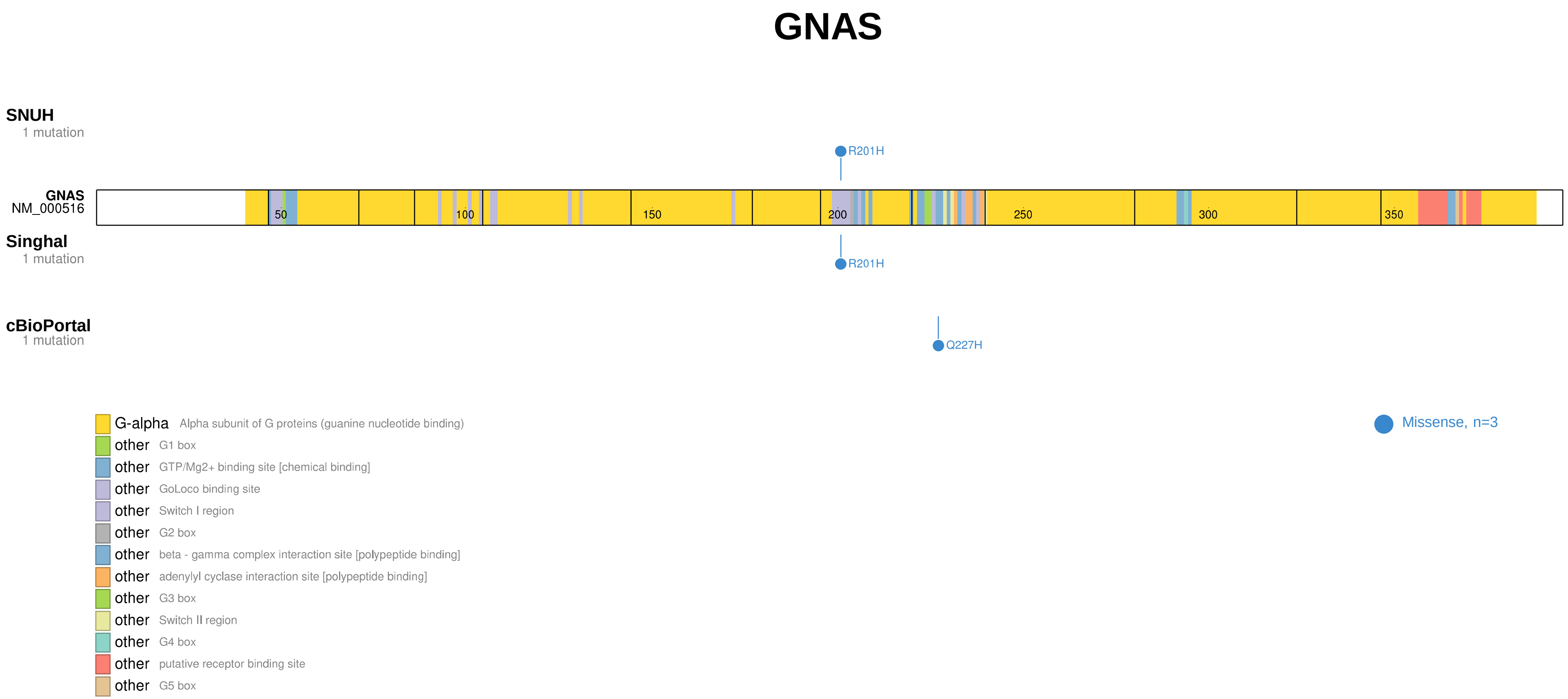
GNAS
SNUH
1 mutation
R201H
GNAS
NM_000516
50
100
150
200
250
300
350
Singhal
1 mutation
R201H
cBioPortal
1 mutation
Q227H
G-alpha Alpha subunit of G proteins (guanine nucleotide binding)
other G1 box
other GTP/Mg2+ binding site [chemical binding]
other GoLoco binding site
other Switch I region
other G2 box
other beta - gamma complex interaction site [polypeptide binding]
other adenylyl cyclase interaction site [polypeptide binding]
other G3 box
other Switch II region
other G4 box
other putative receptor binding site
other G5 box
Missense, n=3

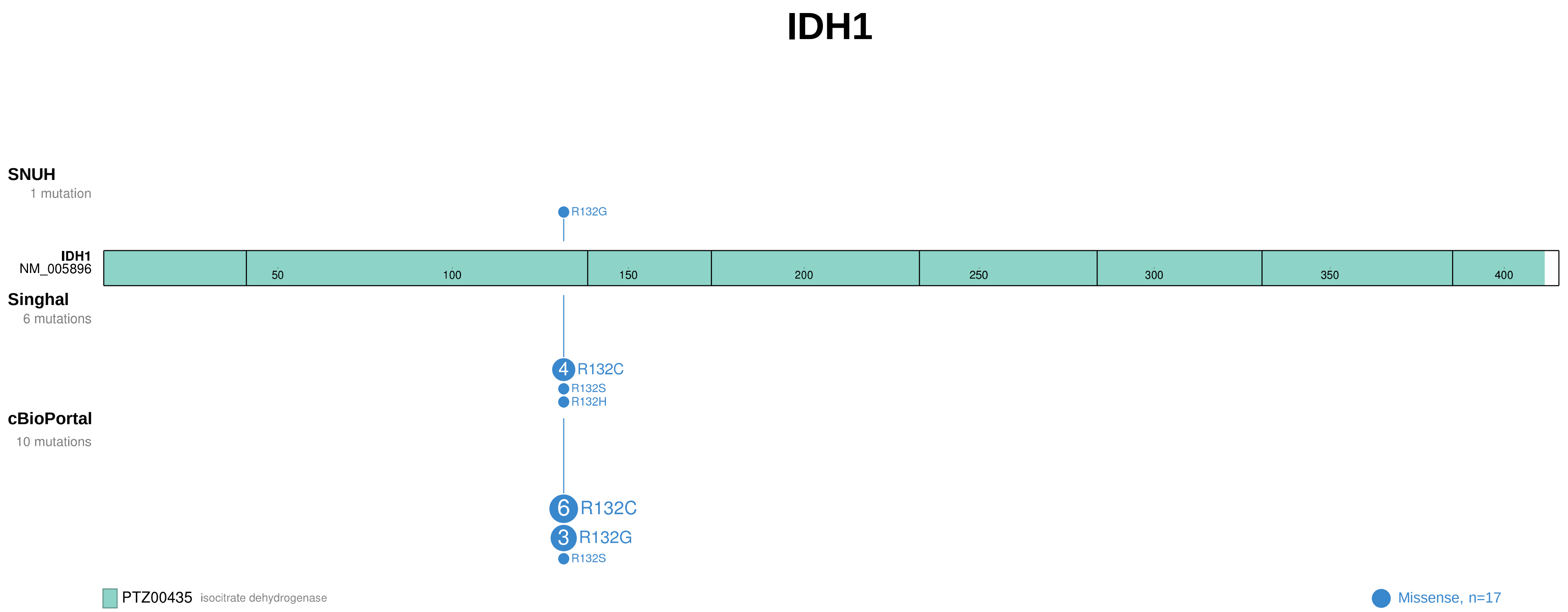

IDH1
SNUH
1 mutation
R132G
IDH1
NM_005896
50
100
150
200
250
300
350
400
Singhal
6 mutations
4 R132C
R132S
R132H
cBioPortal
10 mutations
6 R132C
3 R132G
R132S
PTZ00435 isocitrate dehydrogenase
Missense, n=17

IDH2

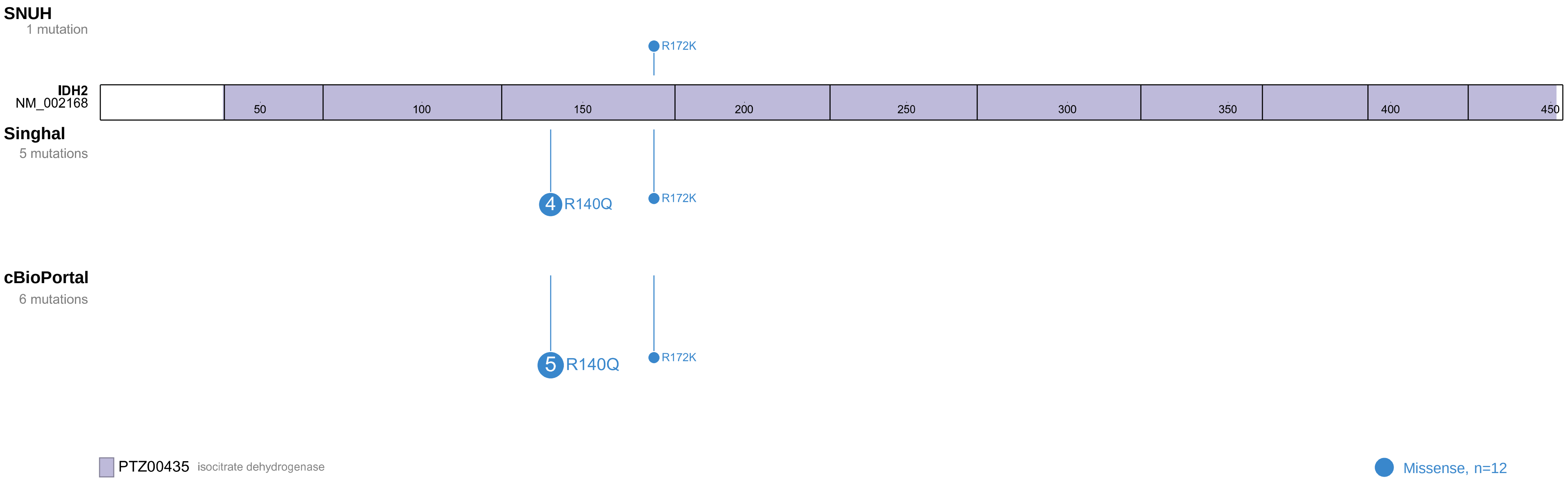
SNUH
1 mutation
R172K
IDH2
NM_002168
50
100
150
200
250
300
350
400
450
Singhal
5 mutations
4 R140Q
R172K
cBioPortal
6 mutations
5 R140Q
R172K
PTZ00435 isocitrate dehydrogenase
Missense, n=12

JAK2

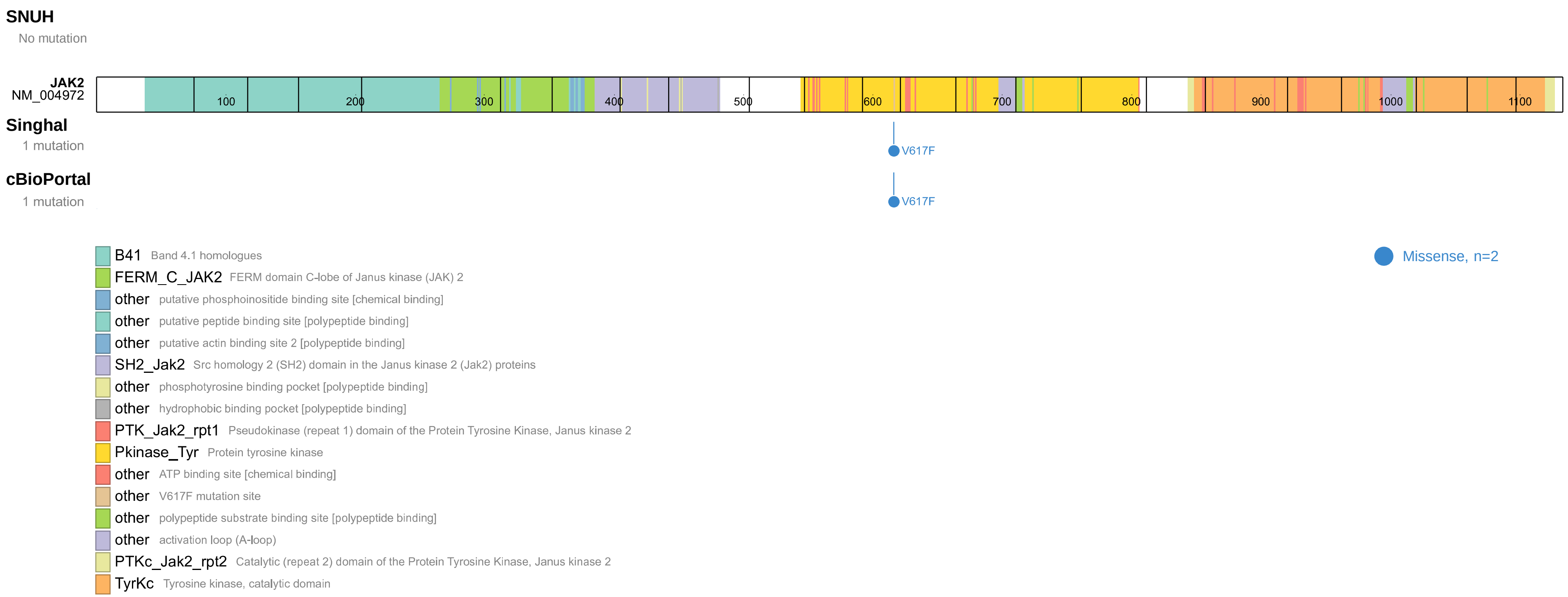

SNUH
No mutation
JAK2
NM_004972
100
200
300
400
500
600
700
800
900
1000
1100
Singhal
1 mutation
V617F
cBioPortal
1 mutation
V617F
B41 Band 4.1 homologues
FERM_C_JAK2 FERM domain C-lobe of Janus kinase (JAK) 2
other putative phosphoinositide binding site [chemical binding]
other putative peptide binding site [polypeptide binding]
other putative actin binding site 2 [polypeptide binding]
SH2_Jak2 Src homology 2 (SH2) domain in the Janus kinase 2 (Jak2) proteins
other phosphotyrosine binding pocket [polypeptide binding]
other hydrophobic binding pocket [polypeptide binding]
PTK_Jak2_rpt1 Pseudokinase (repeat 1) domain of the Protein Tyrosine Kinase, Janus kinase 2
Pkinase_Tyr Protein tyrosine kinase
other ATP binding site [chemical binding]
other V617F mutation site
other polypeptide substrate binding site [polypeptide binding]
other activation loop (A-loop)
PTKc_Jak2_rpt2 Catalytic (repeat 2) domain of the Protein Tyrosine Kinase, Janus kinase 2
TyrKc Tyrosine kinase, catalytic domain
Missense, n=2

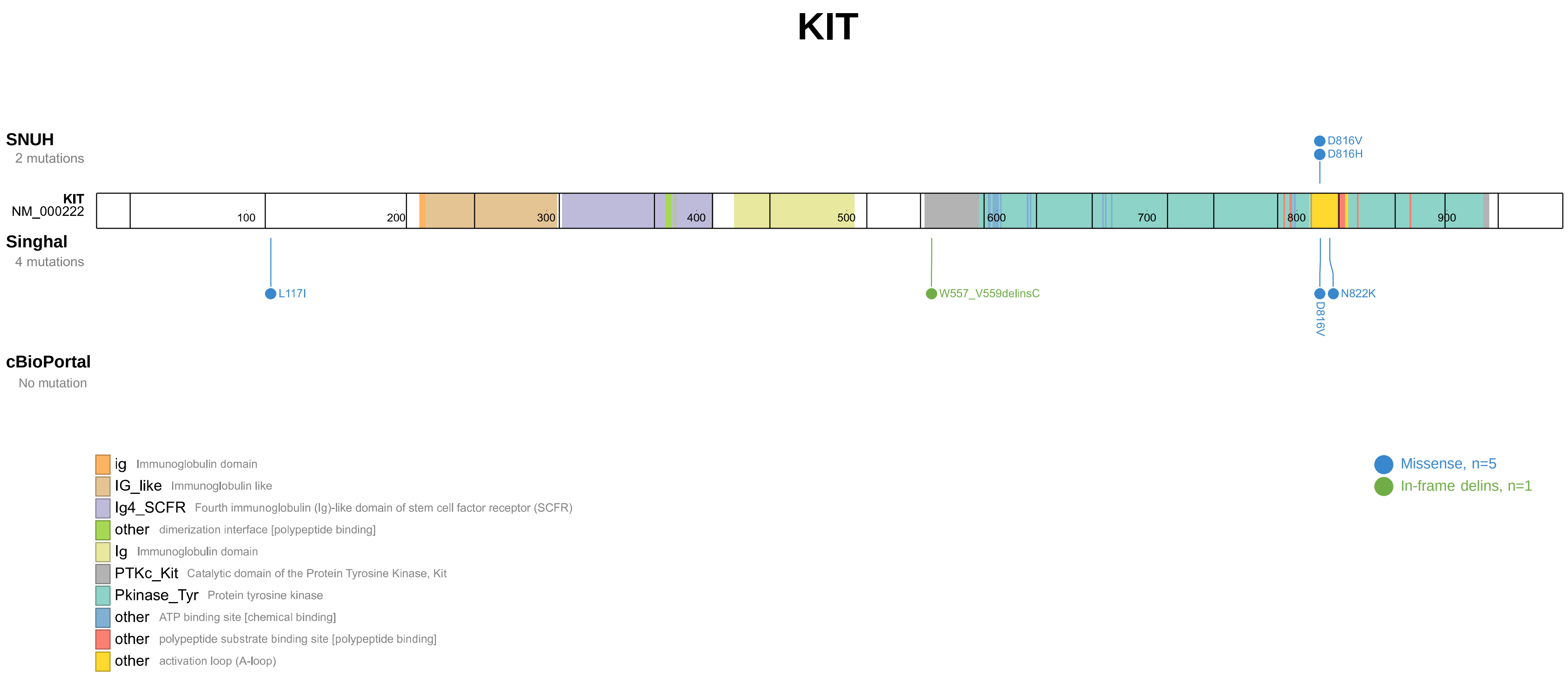
KIT
SNUH
2 mutations
D816V
D816H
KIT
NM_000222
100
200
300
400
500
600
700
800
900
Singhal
4 mutations
L117I
W557_V559delinsC
D816V
N822K
cBioPortal
No mutation
ig Immunoglobulin domain
IG_like Immunoglobulin like
Ig4_SCFR Fourth immunoglobulin (Ig)-like domain of stem cell factor receptor (SCFR)
other dimerization interface [polypeptide binding]
Ig Immunoglobulin domain
PTKc_Kit Catalytic domain of the Protein Tyrosine Kinase, Kit
Pkinase_Tyr Protein tyrosine kinase
other ATP binding site [chemical binding]
other polypeptide substrate binding site [polypeptide binding]
other activation loop (A-loop)
Missense, n=5
In-frame delins, n=1

KMT2A

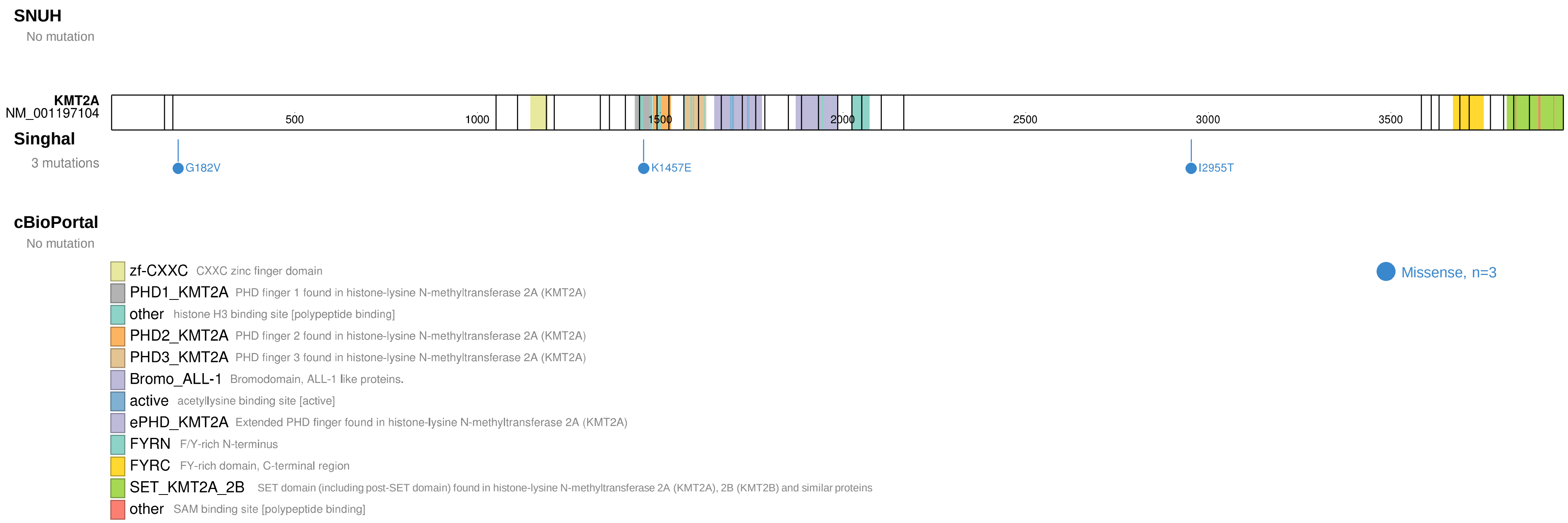
SNUH
No mutation
KMT2A
NM_001197104
500
1000
1500
2000
2500
3000
3500
Singhal
3 mutations
G182V
K1457E
I2955T
cBioPortal
No mutation
zf-CXXC CXXC zinc finger domain
PHD1_KMT2A PHD finger 1 found in histone-lysine N-methyltransferase 2A (KMT2A)
other histone H3 binding site [polypeptide binding]
PHD2_KMT2A PHD finger 2 found in histone-lysine N-methyltransferase 2A (KMT2A)
PHD3_KMT2A PHD finger 3 found in histone-lysine N-methyltransferase 2A (KMT2A)
Bromo_ALL-1 Bromodomain, ALL-1 like proteins.
active acetyllysine binding site [active]
ePHD_KMT2A Extended PHD finger found in histone-lysine N-methyltransferase 2A (KMT2A)
FYRN F/Y-rich N-terminus
FYRC FY-rich domain, C-terminal region
SET_KMT2A_2B SET domain (including post-SET domain) found in histone-lysine N-methyltransferase 2A (KMT2A), 2B (KMT2B) and similar proteins
other SAM binding site [polypeptide binding]
Missense, n=3

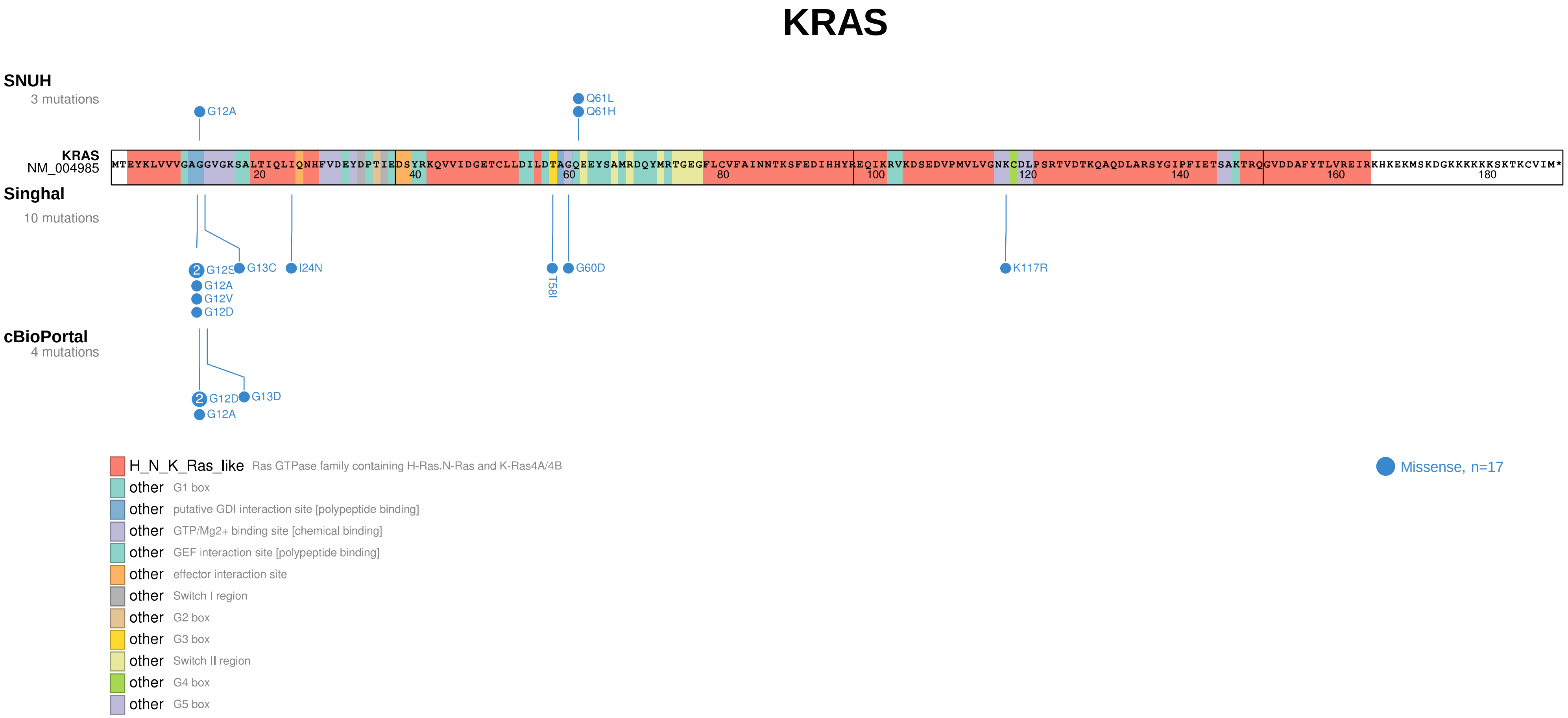
KRAS
SNUH
3 mutations
G12A
Q61L
Q61H
KRAS
NM_004985
Singhal
10 mutations
2 G12S
G13C
I24N
G12A
G12V
G12D
T58I
G60D
K117R
cBioPortal
4 mutations
2 G12D
G13D
G12A
H_N_K_Ras_like Ras GTPase family containing H-Ras,N-Ras and K-Ras4A/4B
other G1 box
other putative GDI interaction site [polypeptide binding]
other GTP/Mg2+ binding site [chemical binding]
other GEF interaction site [polypeptide binding]
other effector interaction site
other Switch I region
other G2 box
other G3 box
other Switch II region
other G4 box
other G5 box
Missense, n=17

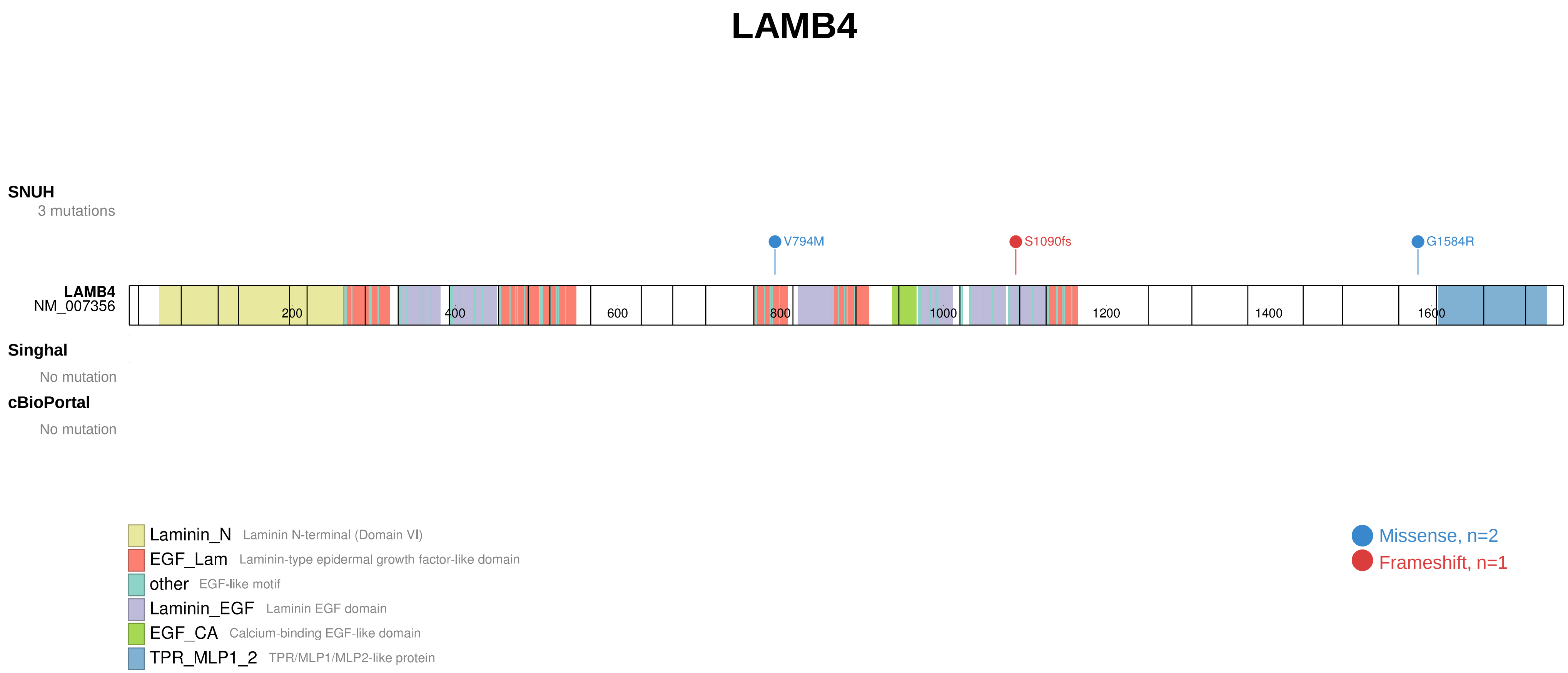
LAMB4
SNUH
3 mutations
V794M
S1090fs
G1584R
LAMB4
NM_007356
200
400
600
800
1000
1200
1400
1600
Singhal
No mutation
cBioPortal
No mutation
Laminin_N Laminin N-terminal (Domain VI)
EGF_Lam Laminin-type epidermal growth factor-like domain
other EGF-like motif
Laminin_EGF Laminin EGF domain
EGF_CA Calcium-binding EGF-like domain
TPR_MLP1_2 TPR/MLP1/MLP2-like protein
Missense, n=2
Frameshift, n=1

MPL

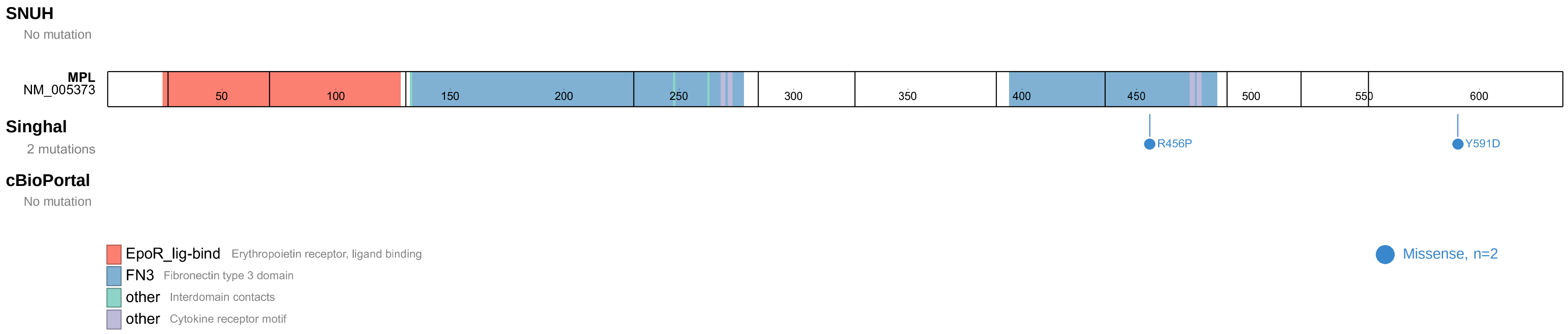
SNUH
No mutation
MPL
NM_005373
50
100
150
200
250
300
350
400
450
500
550
600
Singhal
2 mutations
R456P
Y591D
cBioPortal
No mutation
EpoR_lig-bind Erythropoietin receptor, ligand binding
FN3 Fibronectin type 3 domain
other Interdomain contacts
other Cytokine receptor motif
Missense, n=2

NF1

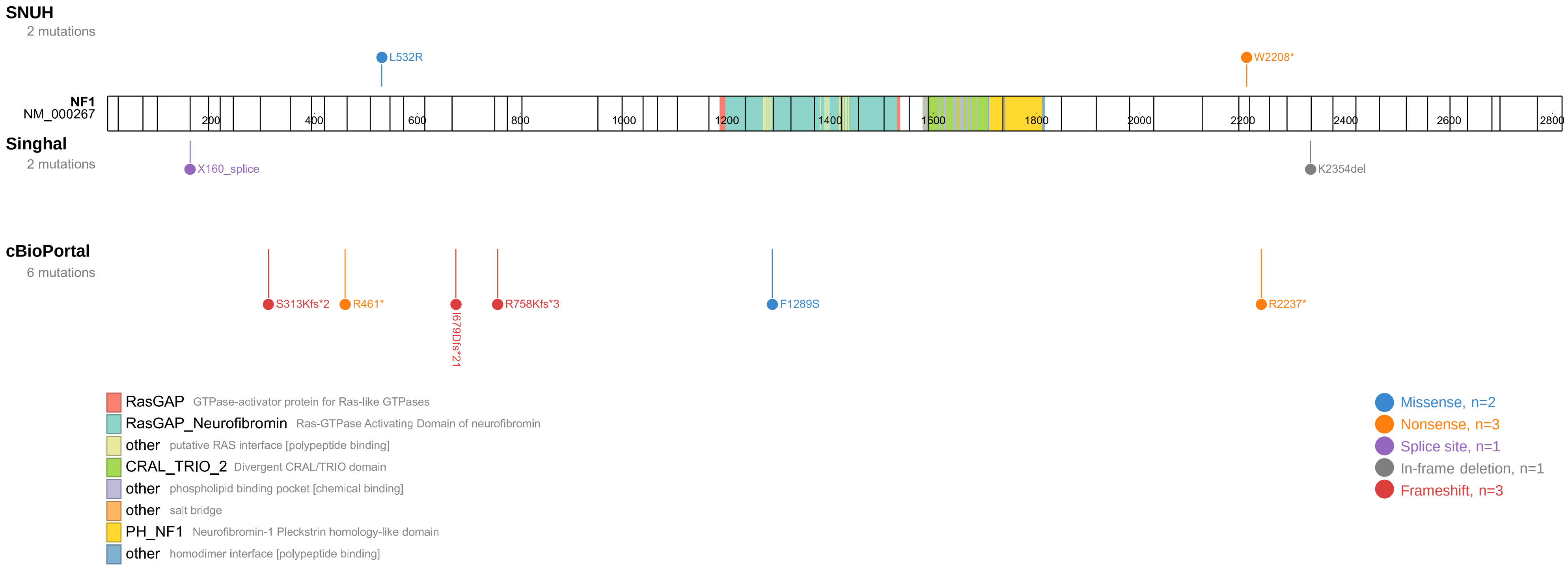
SNUH
2 mutations
L532R
W2208*
NF1
NM_000267
200
400
600
800
1000
1200
1400
1600
1800
2000
2200
2400
2600
2800
Singhal
2 mutations
X160_splice
K2354del
cBioPortal
6 mutations
S313Kfs*2
R461*
I679Dfs*21
R758Kfs*3
F1289S
R2237*
RasGAP GTPase-activator protein for Ras-like GTPases
RasGAP_Neurofibromin Ras-GTPase Activating Domain of neurofibromin
other putative RAS interface [polypeptide binding]
CRAL_TRIO_2 Divergent CRAL/TRIO domain
other phospholipid binding pocket [chemical binding]
other salt bridge
PH_NF1 Neurofibromin-1 Pleckstrin homology-like domain
other homodimer interface [polypeptide binding]
Missense, n=2
Nonsense, n=3
Splice site, n=1
In-frame deletion, n=1
Frameshift, n=3

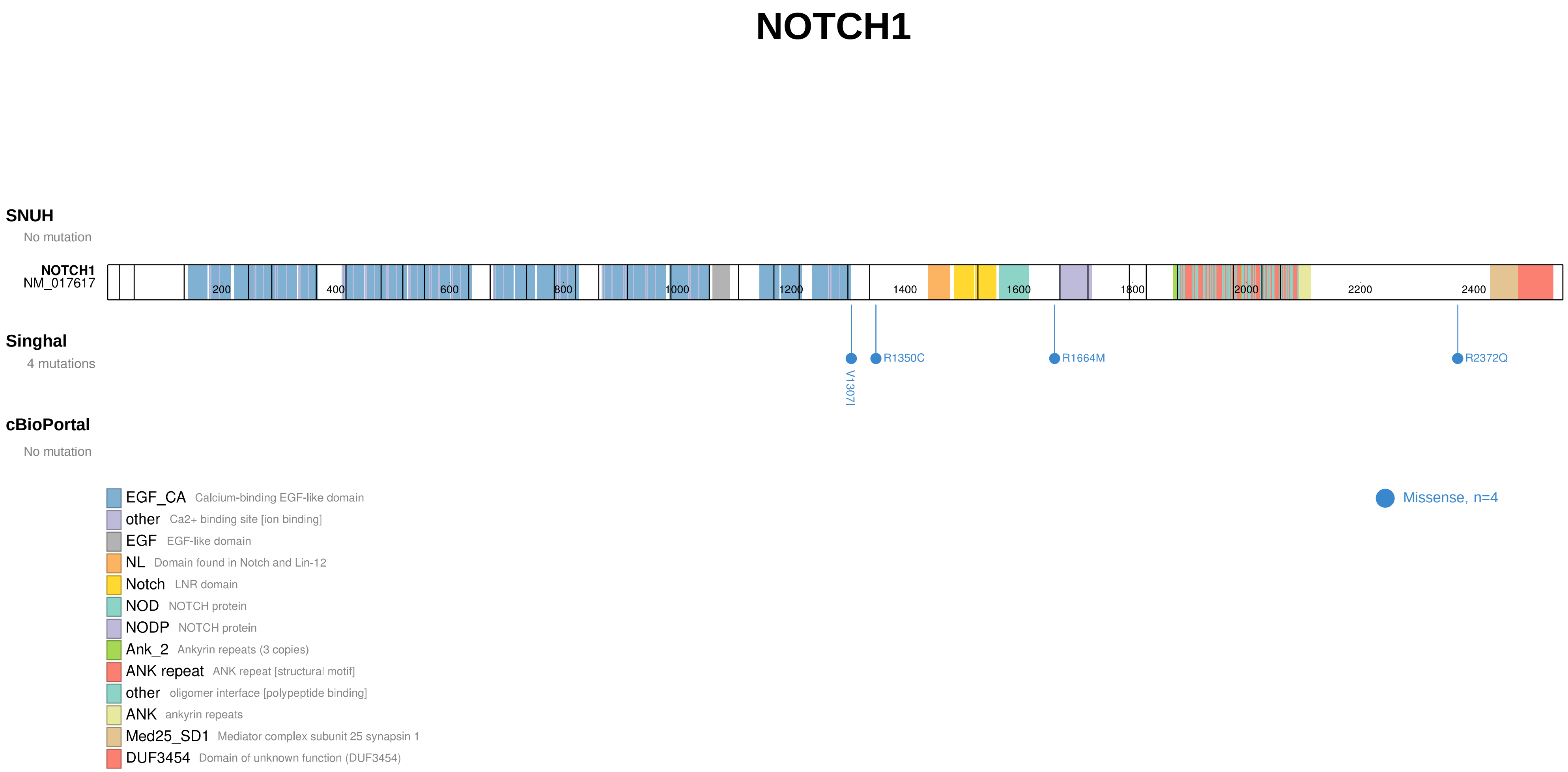
NOTCH1
SNUH
No mutation
NOTCH1
NM_017617
200
400
600
800
1000
1200
1400
1600
1800
2000
2200
2400
Singhal
4 mutations
V1307I
R1350C
R1664M
R2372Q
cBioPortal
No mutation
EGF_CA Calcium-binding EGF-like domain
other Ca2+ binding site [ion binding]
EGF EGF-like domain
NL Domain found in Notch and Lin-12
Notch LNR domain
NOD NOTCH protein
NODP NOTCH protein
Ank_2 Ankyrin repeats (3 copies)
ANK repeat ANK repeat [structural motif]
other oligomer interface [polypeptide binding]
ANK ankyrin repeats
Med25_SD1 Mediator complex subunit 25 synapsin 1
DUF3454 Domain of unknown function (DUF3454)
Missense, n=4

NPM1

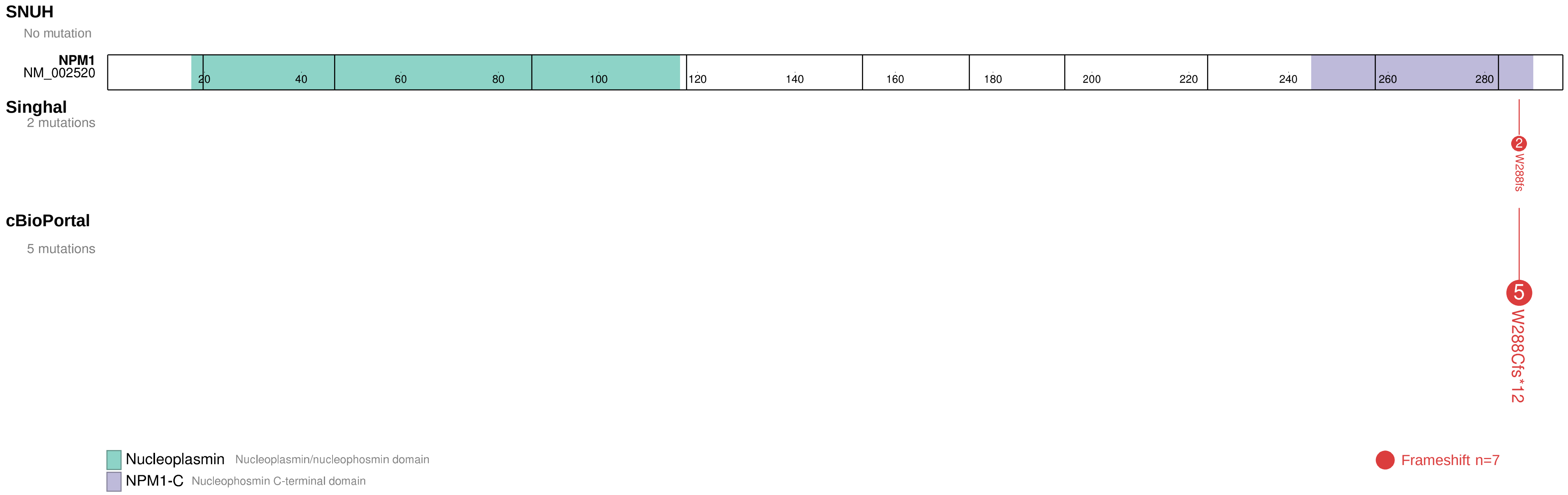

SNUH
No mutation
NPM1
NM_002520
20
40
60
80
100
120
140
160
180
200
220
240
260
280
Singhal
2 mutations
2
W288fs
cBioPortal
5 mutations
5
W288Cfs*12
Nucleoplasmin
Nucleoplasmin/nucleophosmin domain
NPM1-C
Nucleophosmin C-terminal domain
Frameshift n=7

NRAS

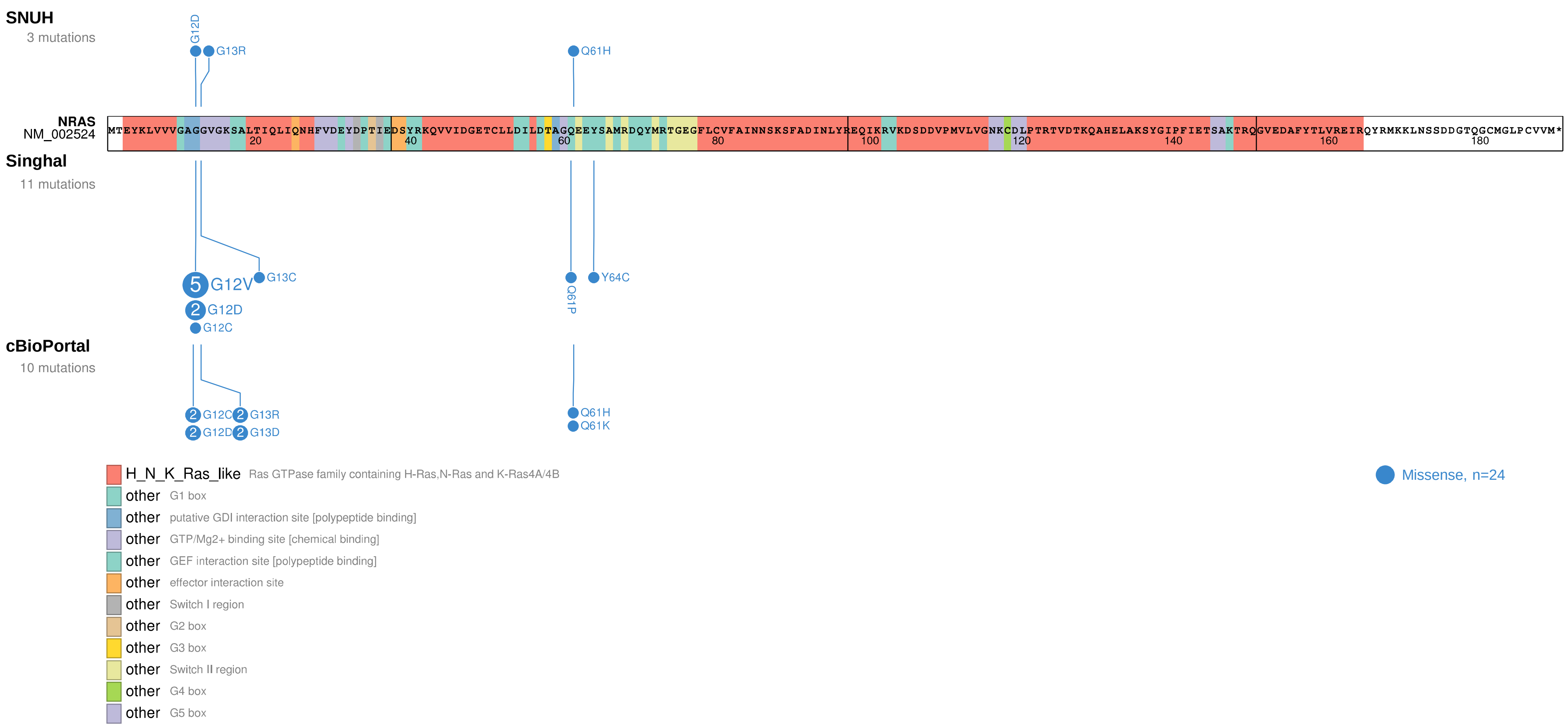
SNUH
3 mutations
G12D
G13R
Q61H
NRAS
NM_002524
MTEYKLVVVGAGGVGKSALTIQLIQNHFVDEYDPTIEDSYRKQVVIDGETCLLDILDTAGQEEYSAMRDQYMRTGEGFLCVFAINNSKSFADINLYREQIKRVKDSDDVPMVLVGNKCDLPTRTVDTKQAHELAKSYGIPFIETSAKTRQGVEDAFYTLVREIRQYRMKKLNSSDDGTQGCMGLPCVVM*
20
40
60
80
100
120
140
160
180
Singhal
11 mutations
G13C
5 G12V
2 G12D
G12C
Q61P
Y64C
cBioPortal
10 mutations
2 G12C
2 G13R
2 G12D
2 G13D
Q61H
Q61K
H_N_K_Ras_like Ras GTPase family containing H-Ras,N-Ras and K-Ras4A/4B
other G1 box
other putative GDI interaction site [polypeptide binding]
other GTP/Mg2+ binding site [chemical binding]
other GEF interaction site [polypeptide binding]
other effector interaction site
other Switch I region
other G2 box
other G3 box
other Switch II region
other G4 box
other G5 box
Missense, n=24

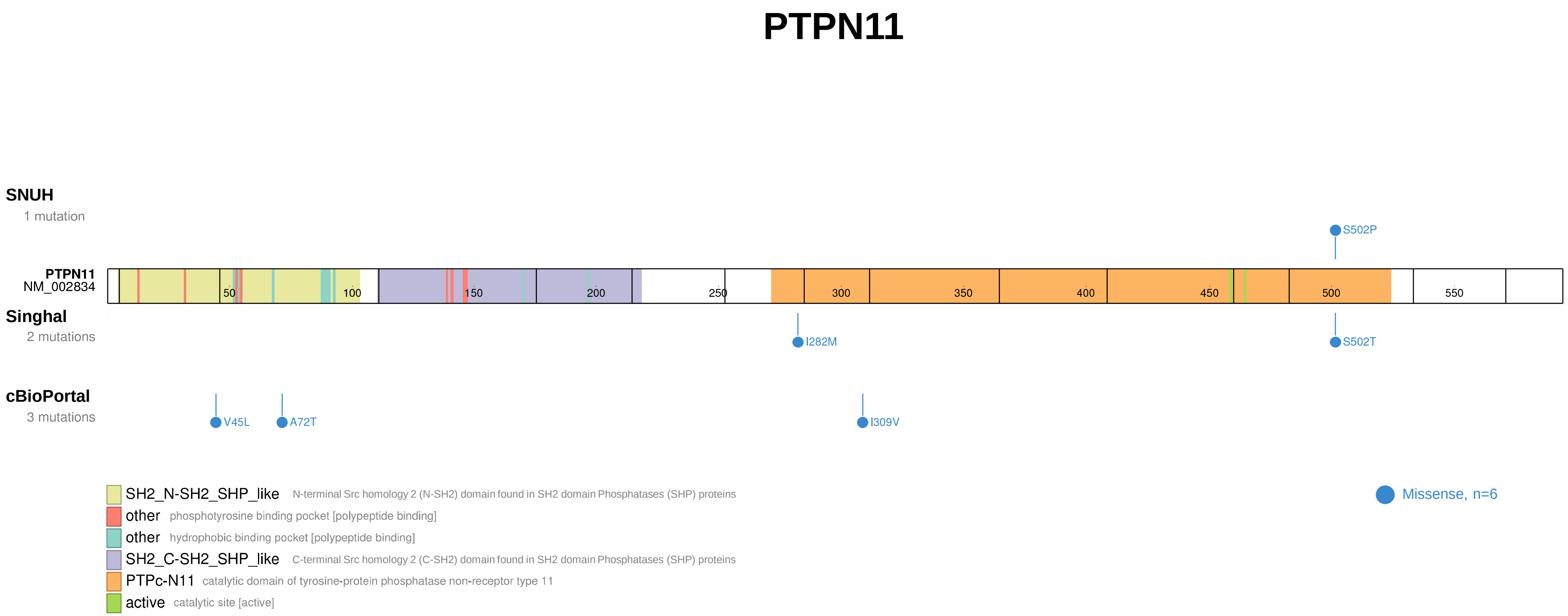
PTPN11
SNUH
1 mutation
S502P
PTPN11
NM_002834
50
100
150
200
250
300
350
400
450
500
550
Singhal
2 mutations
I282M
S502T
cBioPortal
3 mutations
V45L
A72T
I309V
SH2_N-SH2_SHP_like N-terminal Src homology 2 (N-SH2) domain found in SH2 domain Phosphatases (SHP) proteins
other phosphotyrosine binding pocket [polypeptide binding]
other hydrophobic binding pocket [polypeptide binding]
SH2_C-SH2_SHP_like C-terminal Src homology 2 (C-SH2) domain found in SH2 domain Phosphatases (SHP) proteins
PTPc-N11 catalytic domain of tyrosine-protein phosphatase non-receptor type 11
active catalytic site [active]
Missense, n=6

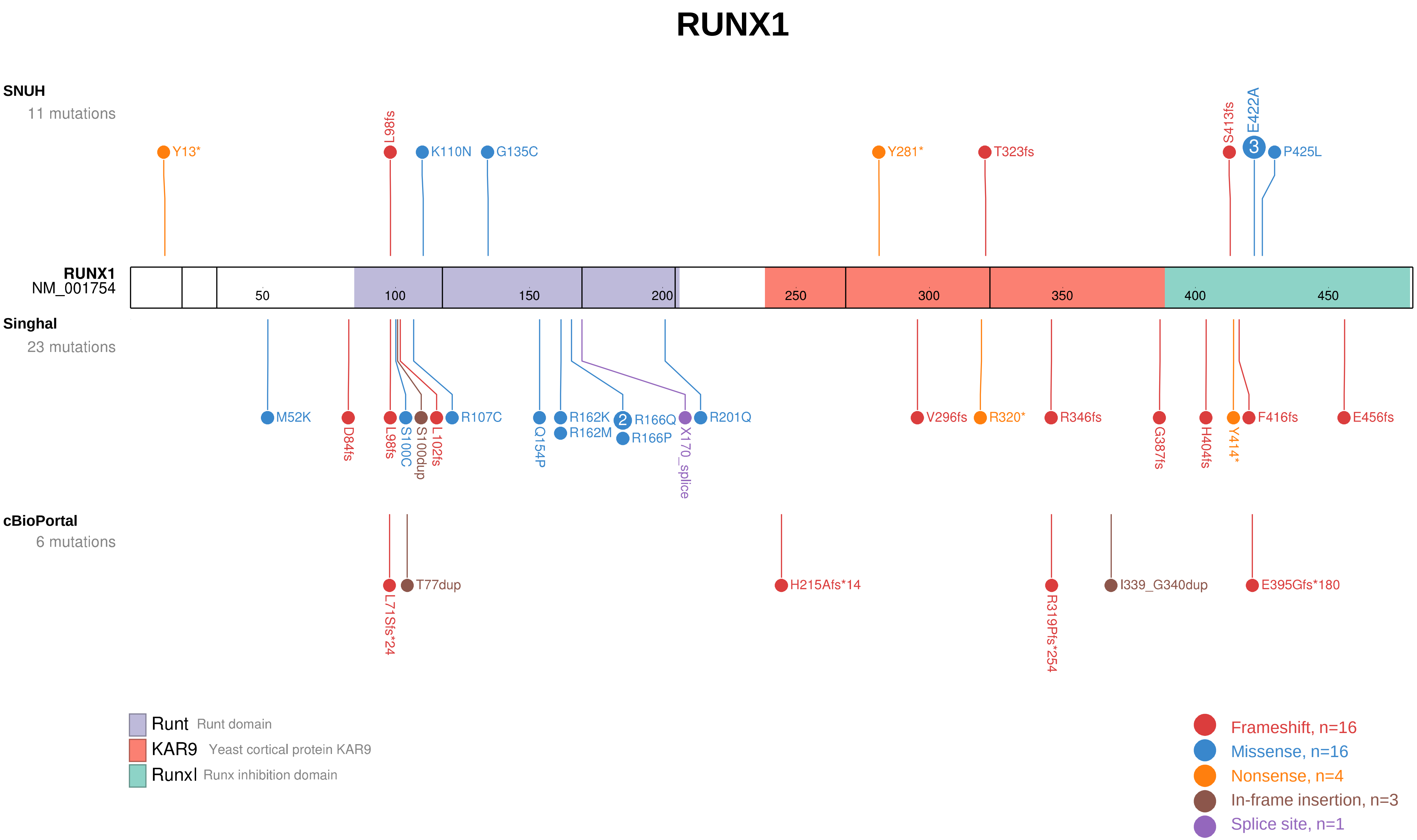

RUNX1
SNUH
11 mutations
Y13*
L98fs
K110N
G135C
Y281*
T323fs
S413fs
E422A
3
P425L
RUNX1
NM_001754
50
100
150
200
250
300
350
400
450
Singhal
23 mutations
M52K
D84fs
L98fs
S100C
S100dup
L102fs
R107C
Q154P
R162K
R162M
2
R166Q
R166P
X170_splice
R201Q
V296fs
R320*
R346fs
G387fs
H404fs
Y414*
F416fs
E456fs
cBioPortal
6 mutations
L71Sfs*24
T77dup
H215Afs*14
R319Pfs*254
I339_G340dup
E395Gfs*180
Runt Runt domain
KAR9 Yeast cortical protein KAR9
RunxI Runx inhibition domain
Frameshift, n=16
Missense, n=16
Nonsense, n=4
In-frame insertion, n=3
Splice site, n=1

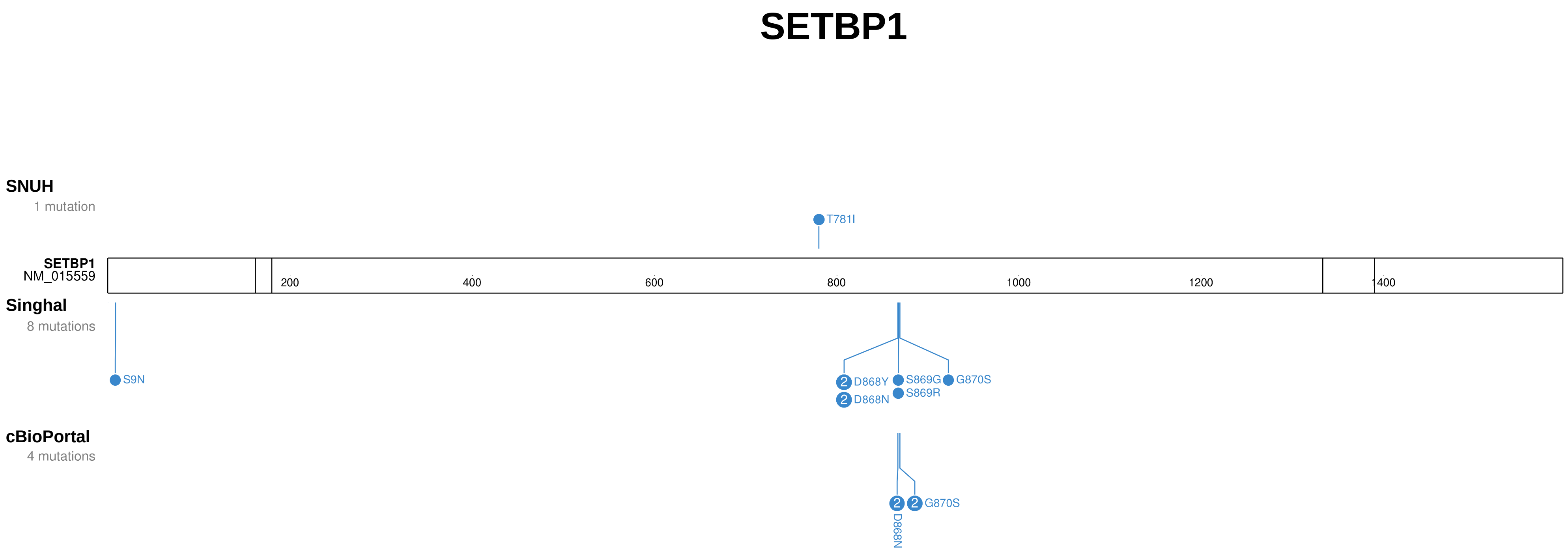
SETBP1
SNUH
1 mutation
T781I
SETBP1
NM_015559
200
400
600
800
1000
1200
1400
Singhal
8 mutations
S9N
2 D868Y
2 D868N
S869G
S869R
G870S
cBioPortal
4 mutations
2
2 G870S
D868N
Missense, n=13

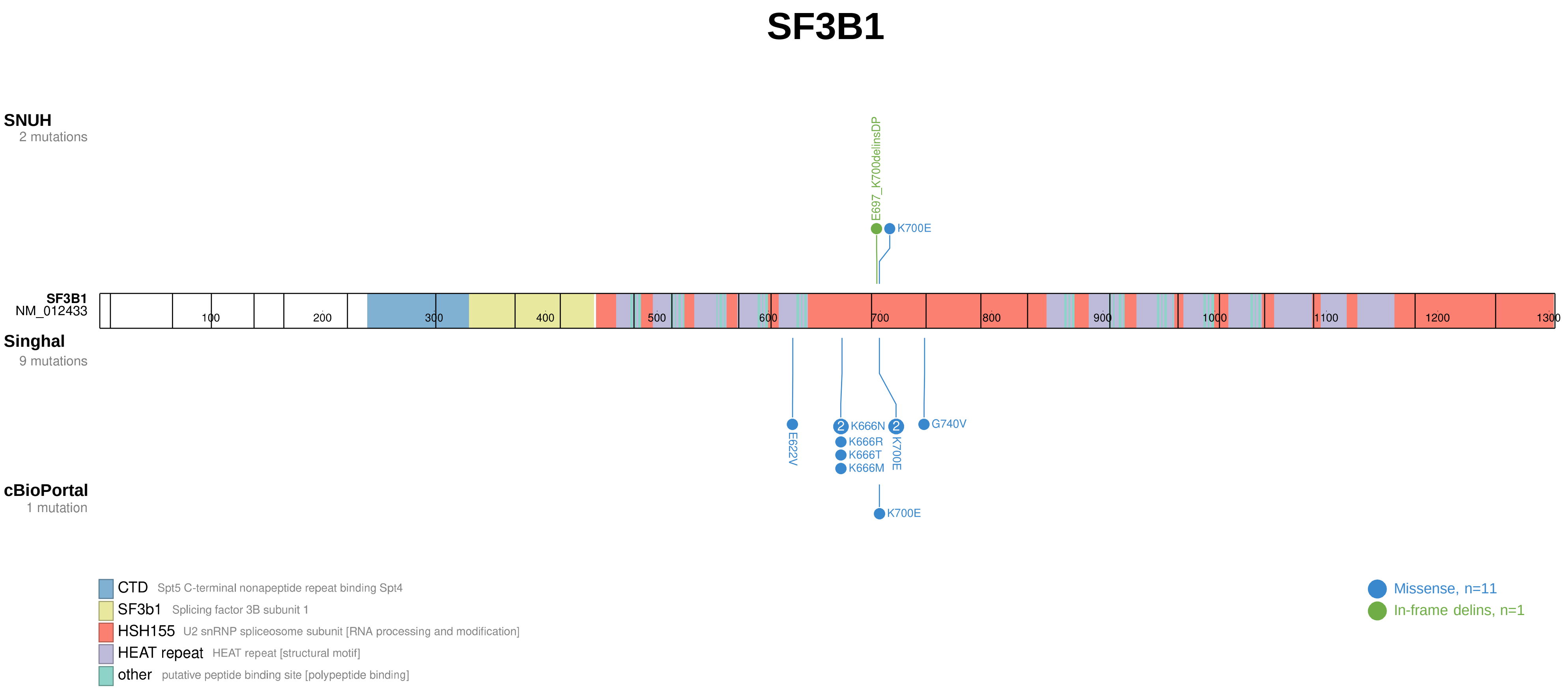
SF3B1
SNUH
2 mutations
E697_K700delinsDP
K700E
SF3B1
NM_012433
100
200
300
400
500
600
700
800
900
1000
1100
1200
1300
Singhal
9 mutations
E622V
2 K666N
K666R
K666T
K666M
2 K700E
G740V
cBioPortal
1 mutation
K700E
CTD Spt5 C-terminal nonapeptide repeat binding Spt4
SF3b1 Splicing factor 3B subunit 1
HSH155 U2 snRNP spliceosome subunit [RNA processing and modification]
HEAT repeat HEAT repeat [structural motif]
other putative peptide binding site [polypeptide binding]
Missense, n=11
In-frame delins, n=1

SRP72

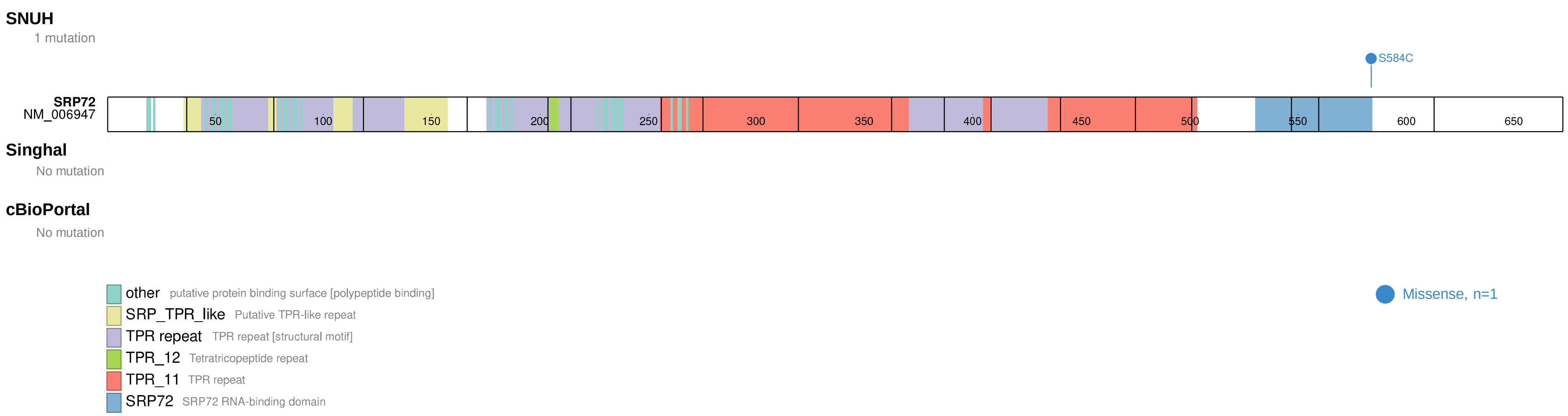
SNUH
1 mutation
S584C
SRP72
NM_006947
50
100
150
200
250
300
350
400
450
500
550
600
650
Singhal
No mutation
cBioPortal
No mutation
other putative protein binding surface [polypeptide binding]
SRP_TPR_like Putative TPR-like repeat
TPR repeat TPR repeat [structural motif]
TPR_12 Tetratricopeptide repeat
TPR_11 TPR repeat
SRP72 SRP72 RNA-binding domain
Missense, n=1

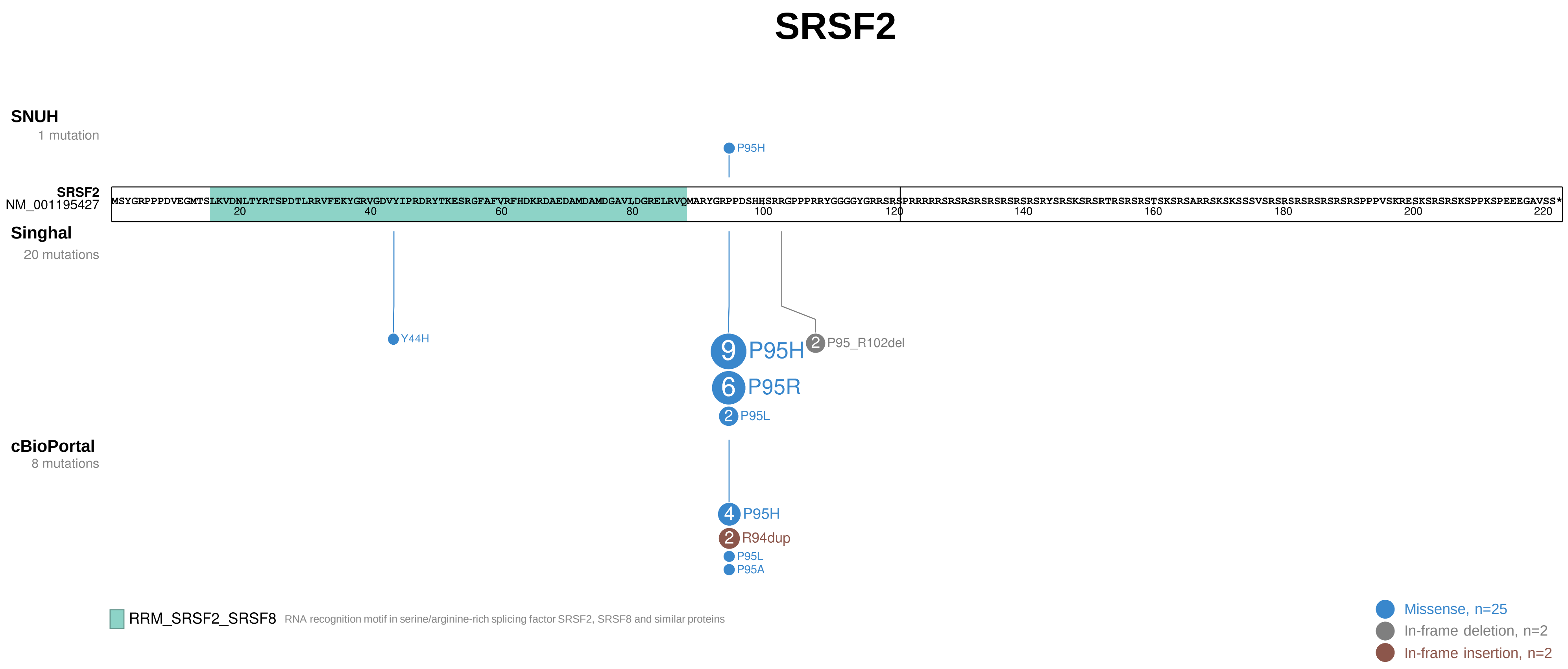
SRSF2
SNUH
1 mutation
P95H
SRSF2
NM_001195427
20
40
60
80
100
120
140
160
180
200
220
Singhal
20 mutations
Y44H
9 P95H
2 P95_R102del
6 P95R
2 P95L
cBioPortal
8 mutations
4 P95H
2 R94dup
P95L
P95A
RRM_SRSF2_SRSF8 RNA recognition motif in serine/arginine-rich splicing factor SRSF2, SRSF8 and similar proteins
Missense, n=25
In-frame deletion, n=2
In-frame insertion, n=2

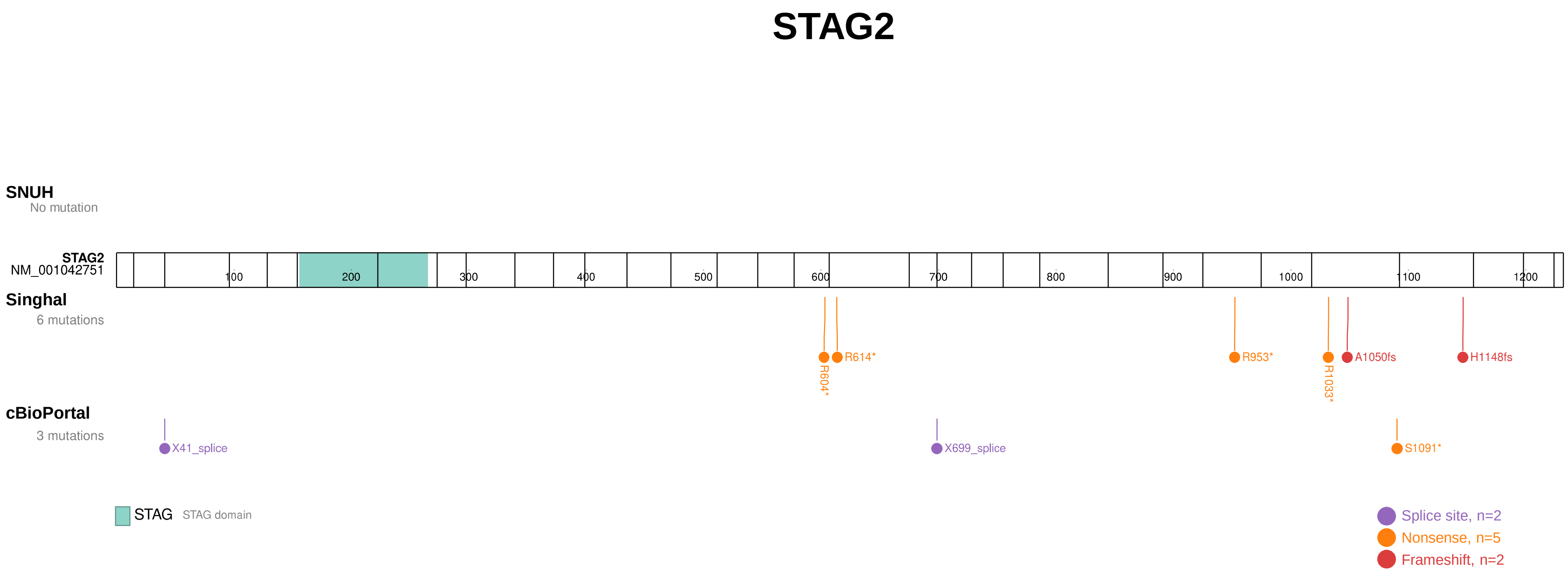

STAG2
SNUH
No mutation
STAG2
NM_001042751
100
200
300
400
500
600
700
800
900
1000
1100
1200
Singhal
6 mutations
R604*
R614*
R953*
R1033*
A1050fs
H1148fs
cBioPortal
3 mutations
X41_splice
X699_splice
S1091*
STAG
STAG domain
Splice site, n=2
Nonsense, n=5
Frameshift, n=2

TERT

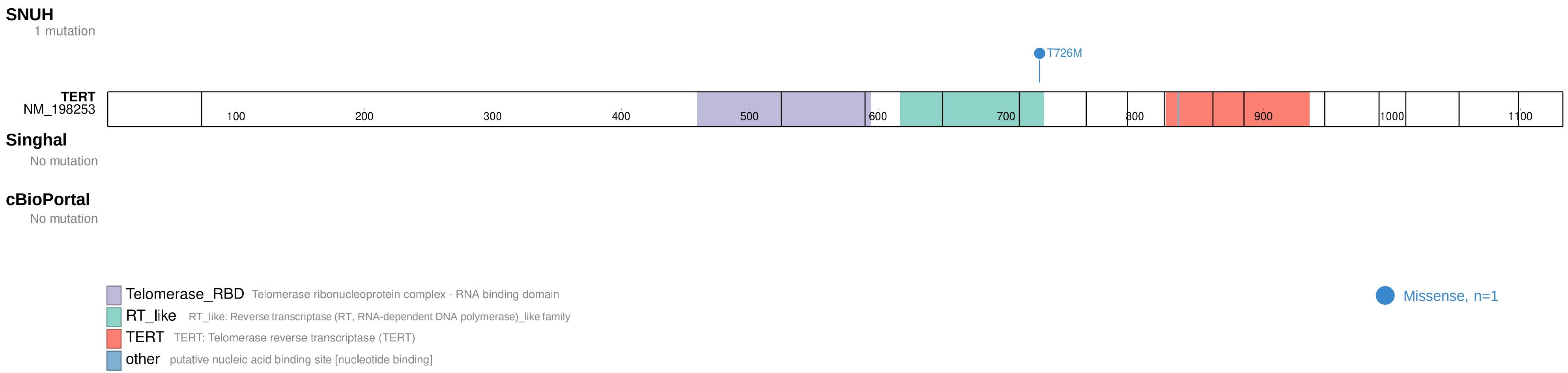
SNUH
1 mutation
T726M
TERT
NM_198253
100
200
300
400
500
600
700
800
900
1000
1100
Singhal
No mutation
cBioPortal
No mutation
Telomerase_RBD Telomerase ribonucleoprotein complex - RNA binding domain
RT_like RT_like: Reverse transcriptase (RT, RNA-dependent DNA polymerase)_like family
TERT TERT: Telomerase reverse transcriptase (TERT)
other putative nucleic acid binding site [nucleotide binding]
Missense, n=1

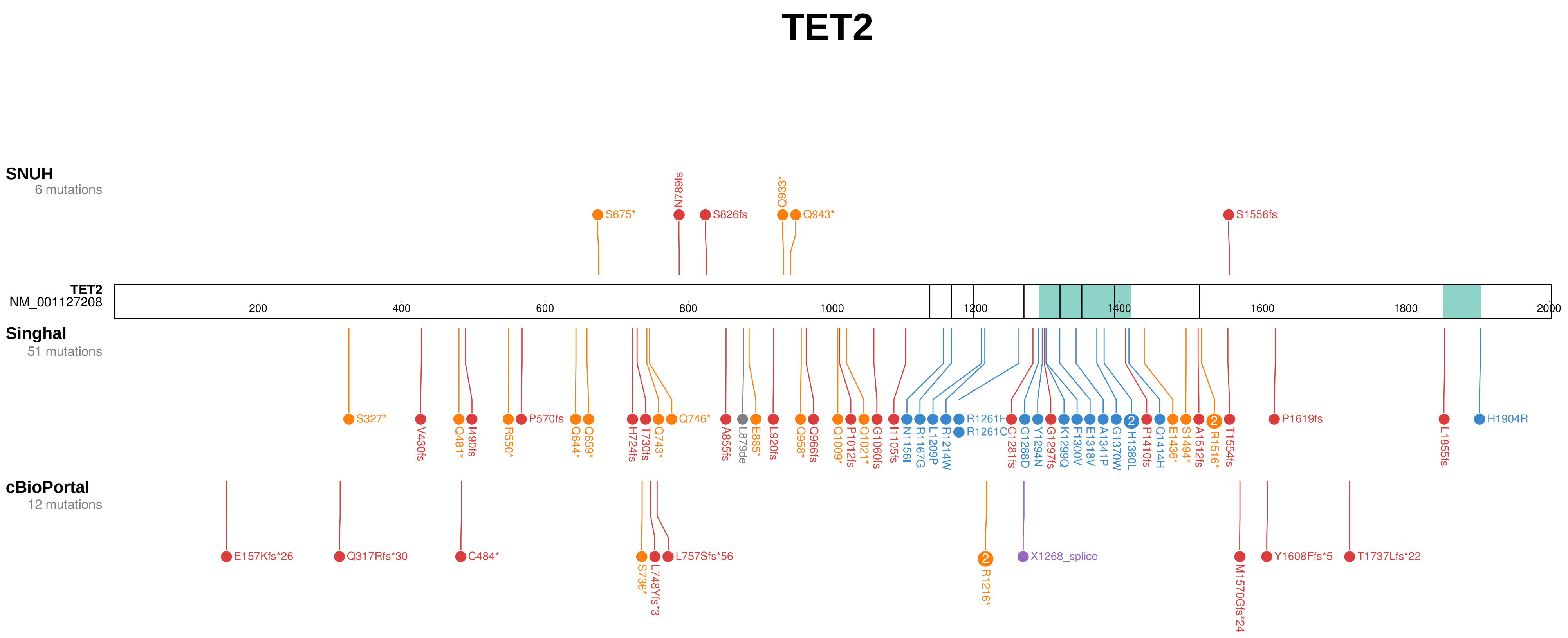
TET2
SNUH
6 mutations
S675*
N789fs
S826fs
Q933*
Q943*
S1556fs
TET2
NM_001127208
200
400
600
800
1000
1200
1400
1600
1800
2000
Singhal
51 mutations
S327*
V430fs
Q481*
I490fs
R550*
P570fs
Q644*
Q659*
H724fs
T730fs
Q743*
Q746*
A855fs
L879del
E885*
L920fs
Q958*
Q966fs
Q1009*
P1012fs
Q1021*
G1060fs
I1105fs
N1156I
R1167G
L1209P
R1214W
R1261H
R1261C
C1281fs
G1288D
Y1294N
G1297fs
K1299Q
F1300V
E1318V
A1341P
G1370W
H1380L
P1410fs
Q1414H
E1436*
S1494*
A1512fs
R1516*
T1554fs
P1619fs
L1855fs
H1904R
cBioPortal
12 mutations
E157Kfs*26
Q317Rfs*30
C484*
S736*
L748Yfs*3
L757Sfs*56
R1216*
X1268_splice
M1570Gfs*24
Y1608Ffs*5
T1737Lfs*22
Tet_JBP Oxygenase domain of the 2OGFeDO superfamily
Nonsense, n=21
Frameshift, n=29
Missense, n=17
In-frame deletion, n=1
Splice site, n=1

U2AF1

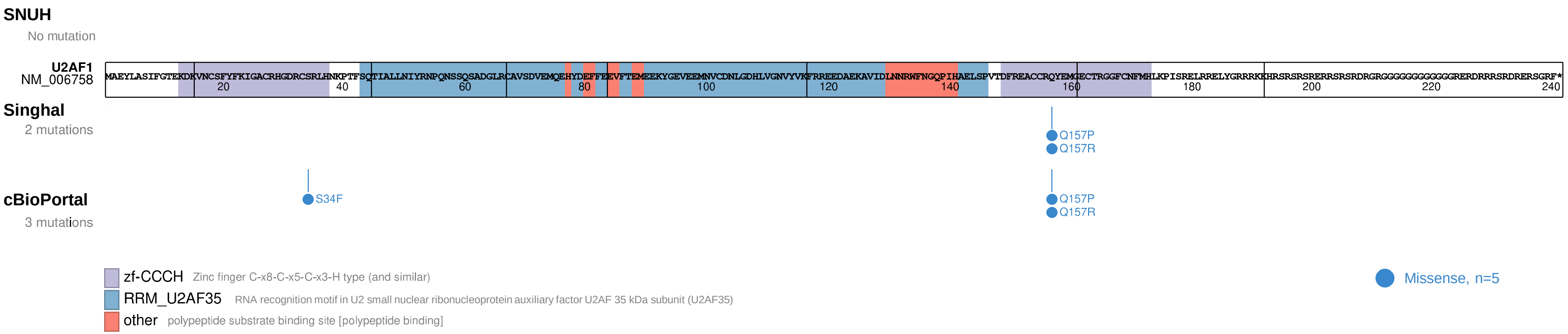

SNUH
No mutation
U2AF1
NM_006758
20
40
60
80
100
120
140
160
180
200
220
240
Singhal
2 mutations
Q157P
Q157R
cBioPortal
3 mutations
S34F
Q157P
Q157R
zf-CCCH Zinc finger C-x8-C-x5-C-x3-H type (and similar)
RRM_U2AF35 RNA recognition motif in U2 small nuclear ribonucleoprotein auxiliary factor U2AF 35 kDa subunit (U2AF35)
other polypeptide substrate binding site [polypeptide binding]
Missense, n=5

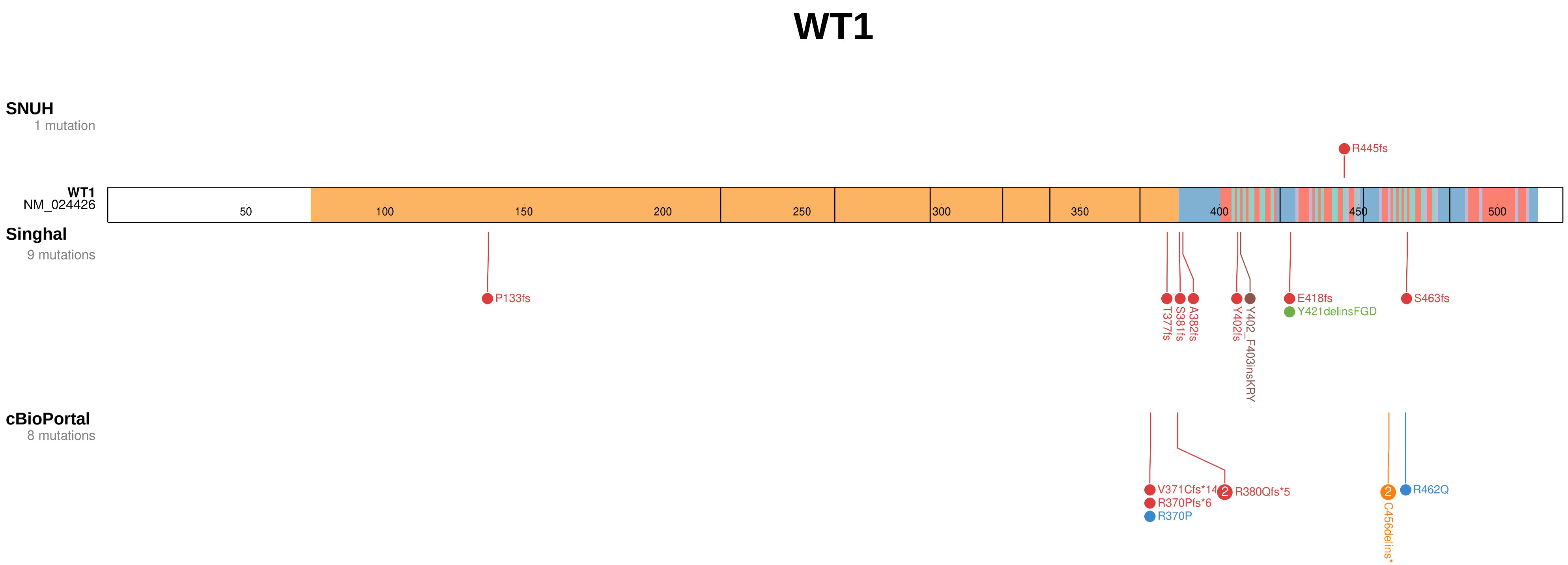
WT1
SNUH
1 mutation
R445fs
WT1
NM_024426
50
100
150
200
250
300
350
400
450
500
Singhal
9 mutations
P133fs
T377fs
S381fs
A382fs
Y402fs
Y402_F403insKRY
E418fs
Y421delinsFGD
S463fs
cBioPortal
8 mutations
V371Cfs*14
R370Pfs*6
R370P
2
R380Qfs*5
2
C456delins*
R462Q
WT1 Wilm's tumor protein
COG5048 FOG: Zn-finger [General function prediction only]
C2H2 Zn finger C2H2 Zn finger [structural motif]
other putative nucleic acid binding site [nucleotide binding]
other Zn binding site [ion binding]
Frameshift, n=12
Missense, n=2
Nonsense, n=2
In-frame insertion, n=1
In-frame delins, n=1

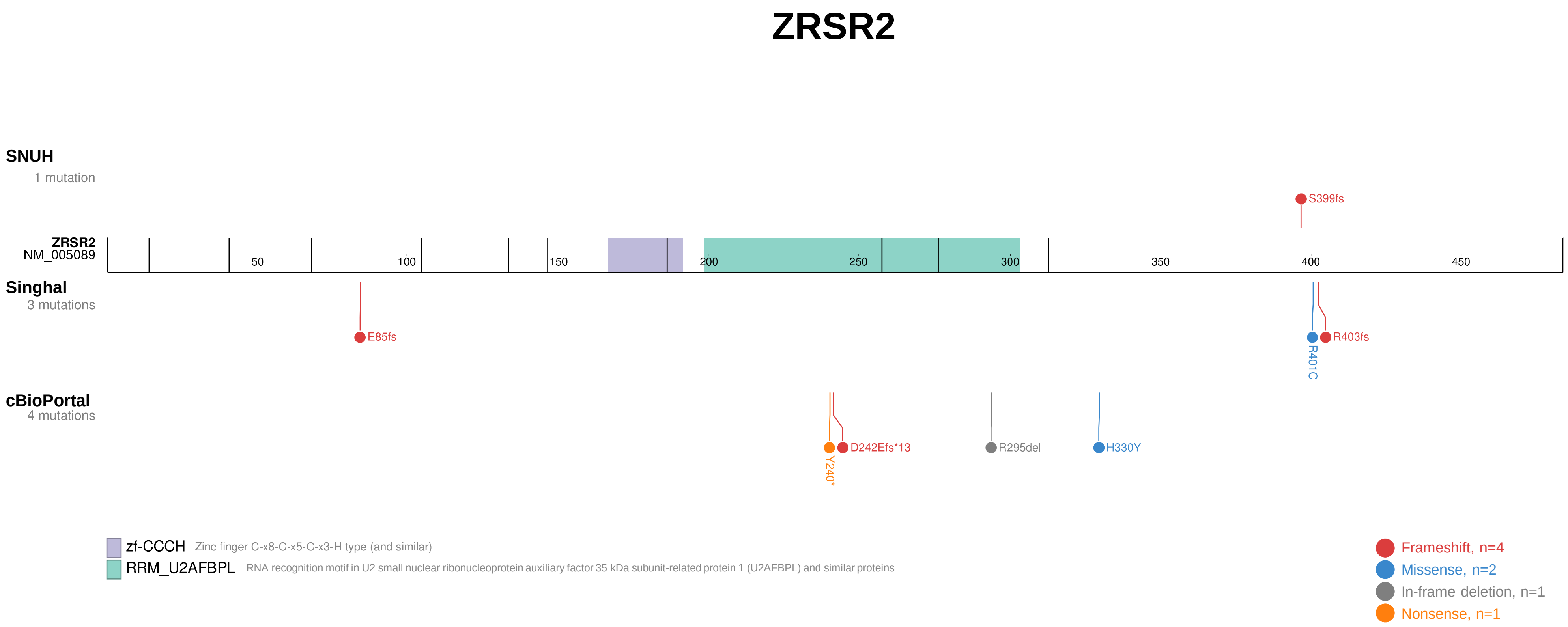
ZRSR2
SNUH
1 mutation
S399fs
ZRSR2
NM_005089
50
100
150
200
250
300
350
400
450
Singhal
3 mutations
E85fs
R401C
R403fs
cBioPortal
4 mutations
Y240*
D242Efs*13
R295del
H330Y
zf-CCCH Zinc finger C-x8-C-x5-C-x3-H type (and similar)
RRM_U2AFBPL RNA recognition motif in U2 small nuclear ribonucleoprotein auxiliary factor 35 kDa subunit-related protein 1 (U2AFBPL) and similar proteins
Frameshift, n=4
Missense, n=2
In-frame deletion, n=1
Nonsense, n=1
