# Supplementary material for: Analysis of clinical and genomic profiles of therapy-related myeloid neoplasm in Korea
Source: Hum Genomics. 2023 Feb 23;17:13. doi: 10.1186/s40246-023-00458-8 (PMC9948421; doi:10.1186/s40246-023-00458-8)
Supplement: Supplementary file 5 — Additional file 5: Fig. S4. Kaplan-Meier survival curves showing insignificant prognostic factors among 53 T-MN patients. Fig. S5. Kaplan-Meier survival curves of significant prognostic factors affecting overall survival in 46 adult T-MN patients. Fig. S6. Kaplan-Meier survival curves showing insignificant prognostic factors among 46 adult T-MN patients. Fig. S7. Forest plot by Multiple Cox Proportional Hazards Model of 46 adult T-MN patients. Fig. S8. Findings of pre-existing clonal hematopoiesis of indeterminate potential and constant or residual somatic variants in morphologic remission in T-MN. [file 40246_2023_458_MOESM5_ESM.pdf]

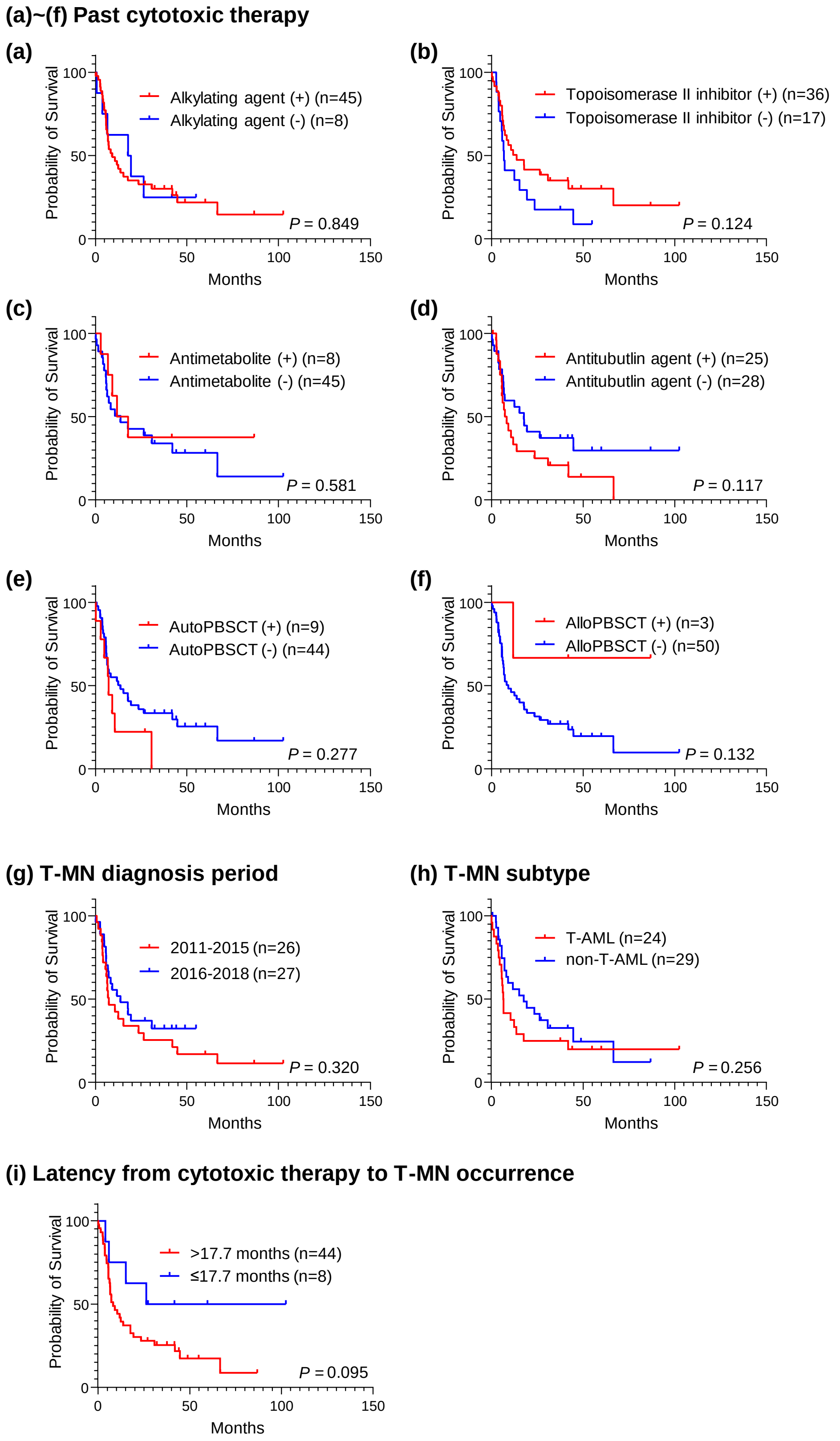

Supplementary Figure 4. Kaplan-Meier survival curves showing insignificant prognostic factors among 53 T-MN patients.

Abbreviations: PBSCT, peripheral blood stem cell transplantation; T-AML, therapy-related acute myeloid leukemia.

(j)~(n) Cytogenetic aberration

(j)

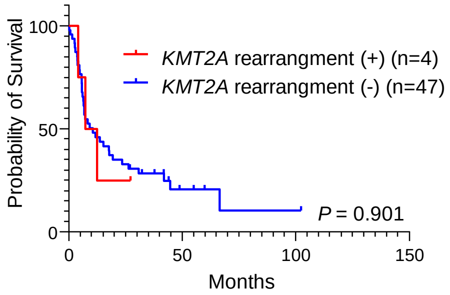

(k)

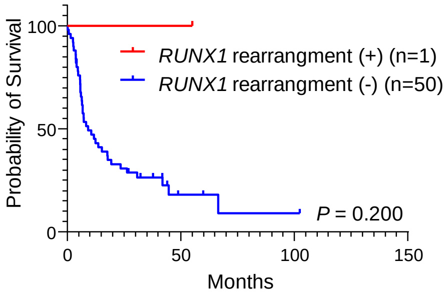

(l)

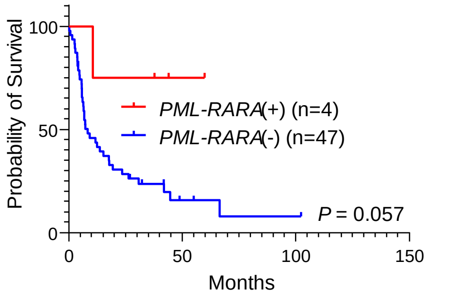

(m)

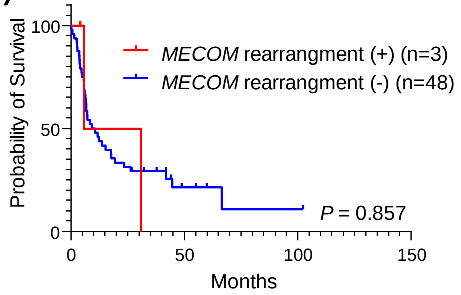

(n) Germline predisposition

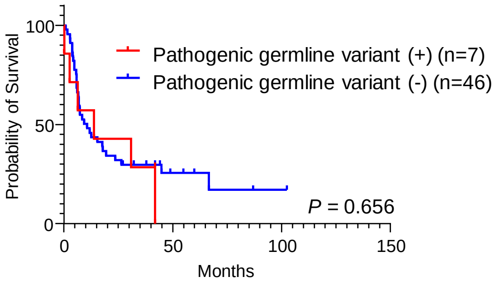

(o)~(r) Somatic mutation (single gene)

(o)

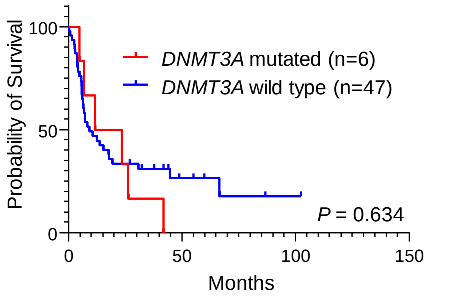

(p)

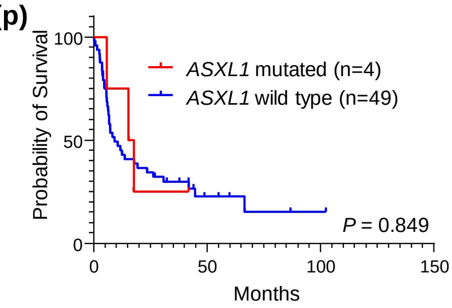

(q)

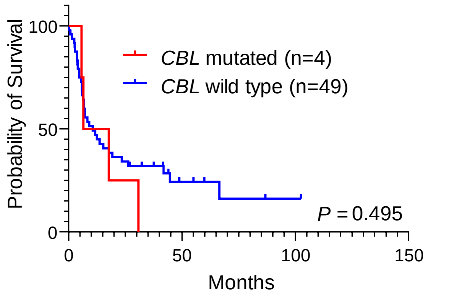

(r)

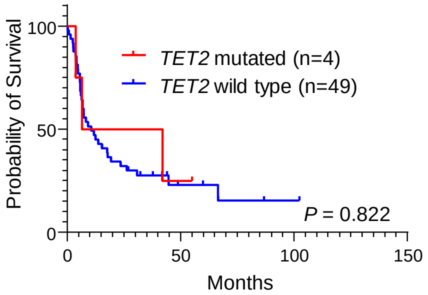

Supplementary Figure 4. Continued.

(s)~(x) Somatic mutation (gene category)

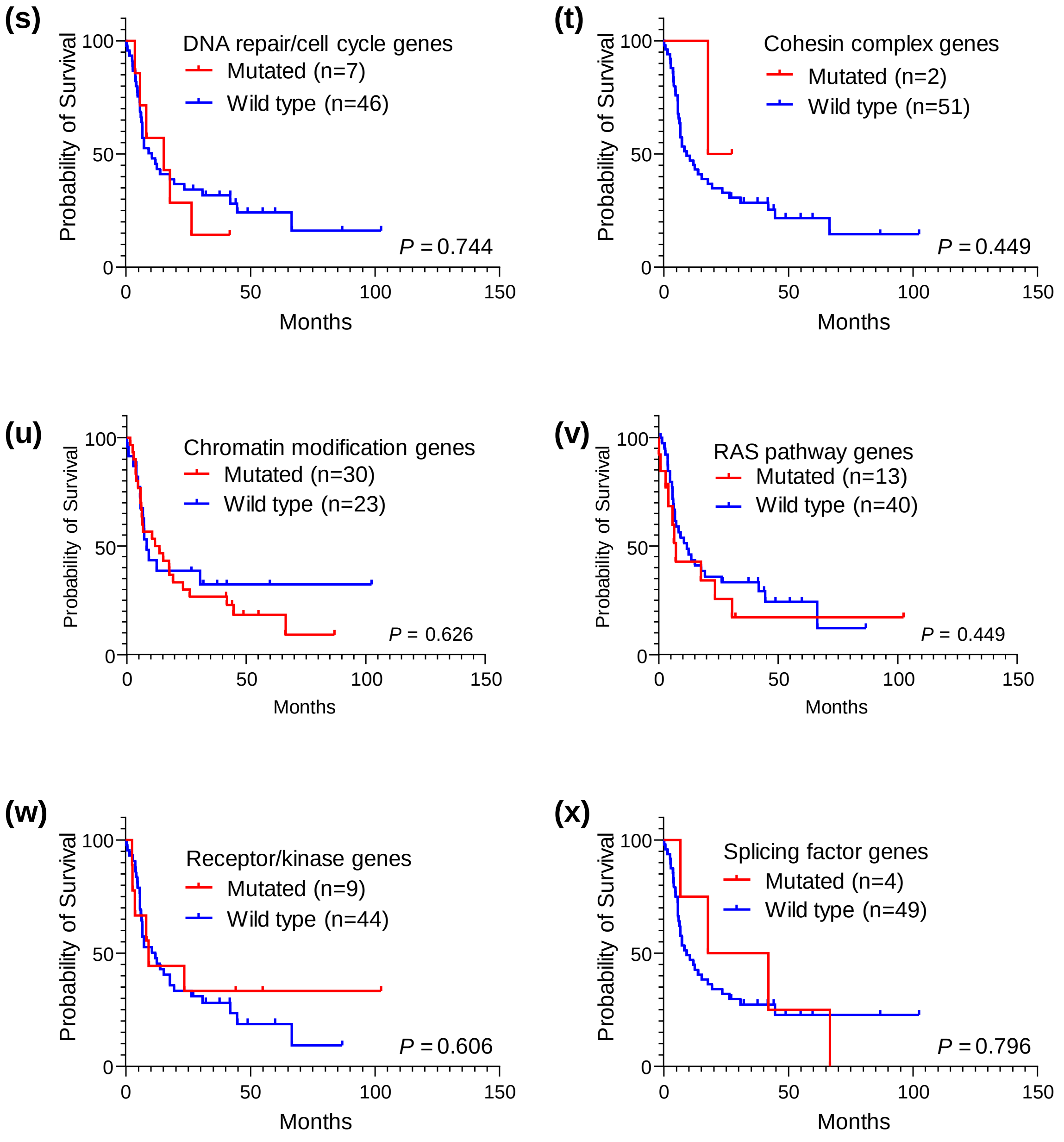

Supplementary Figure 4. Continued.

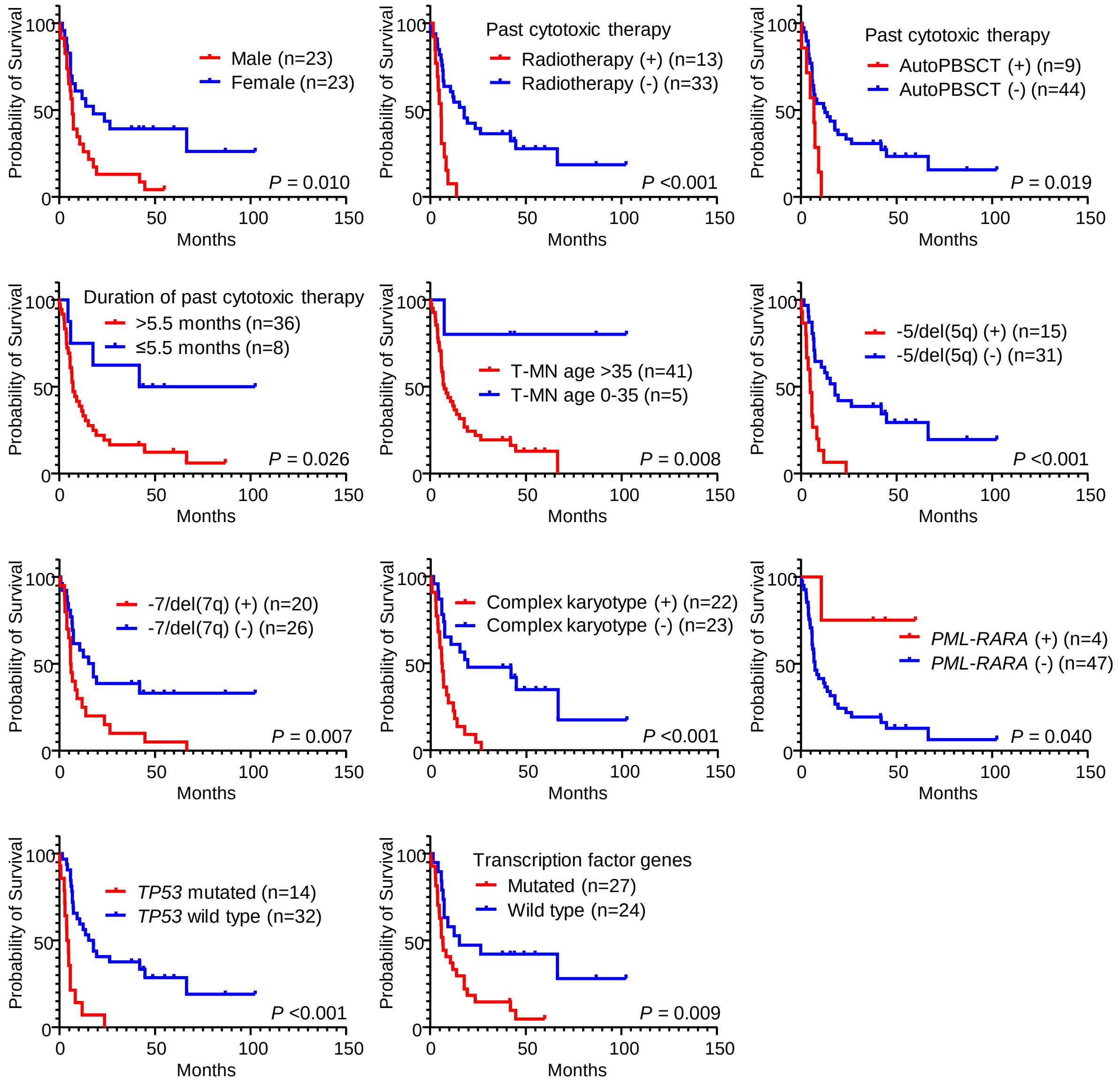

Supplementary Figure 5. Kaplan-Meier survival curves of significant prognostic factors affecting overall survival in 46 adult T-MN patients.

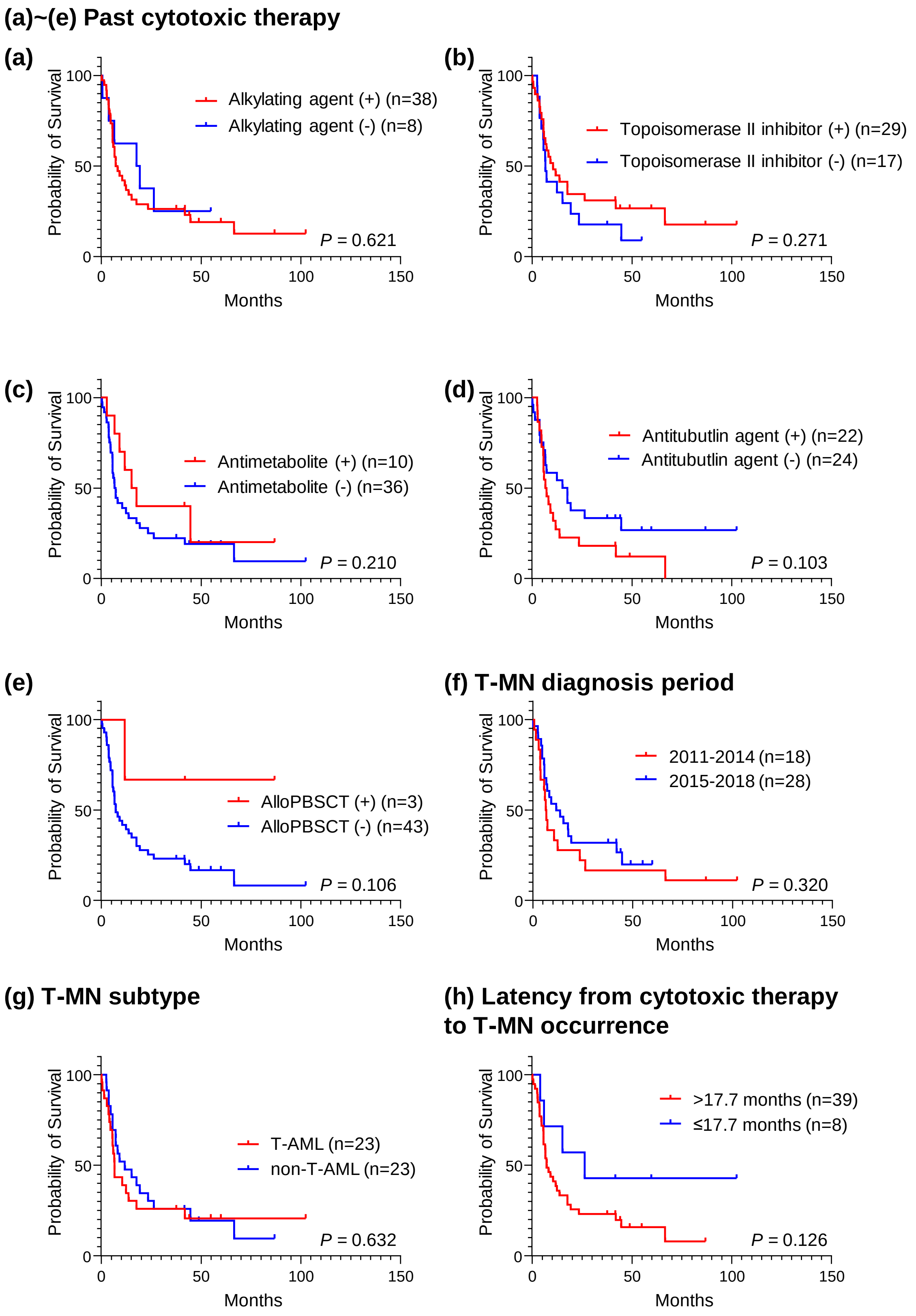

Supplementary Figure 6. Kaplan-Meier survival curves showing insignificant prognostic factors among 46 adult T-MN patients.

Abbreviations: PBSCT, peripheral blood stem cell transplantation; T-AML, therapy-related acute myeloid leukemia.

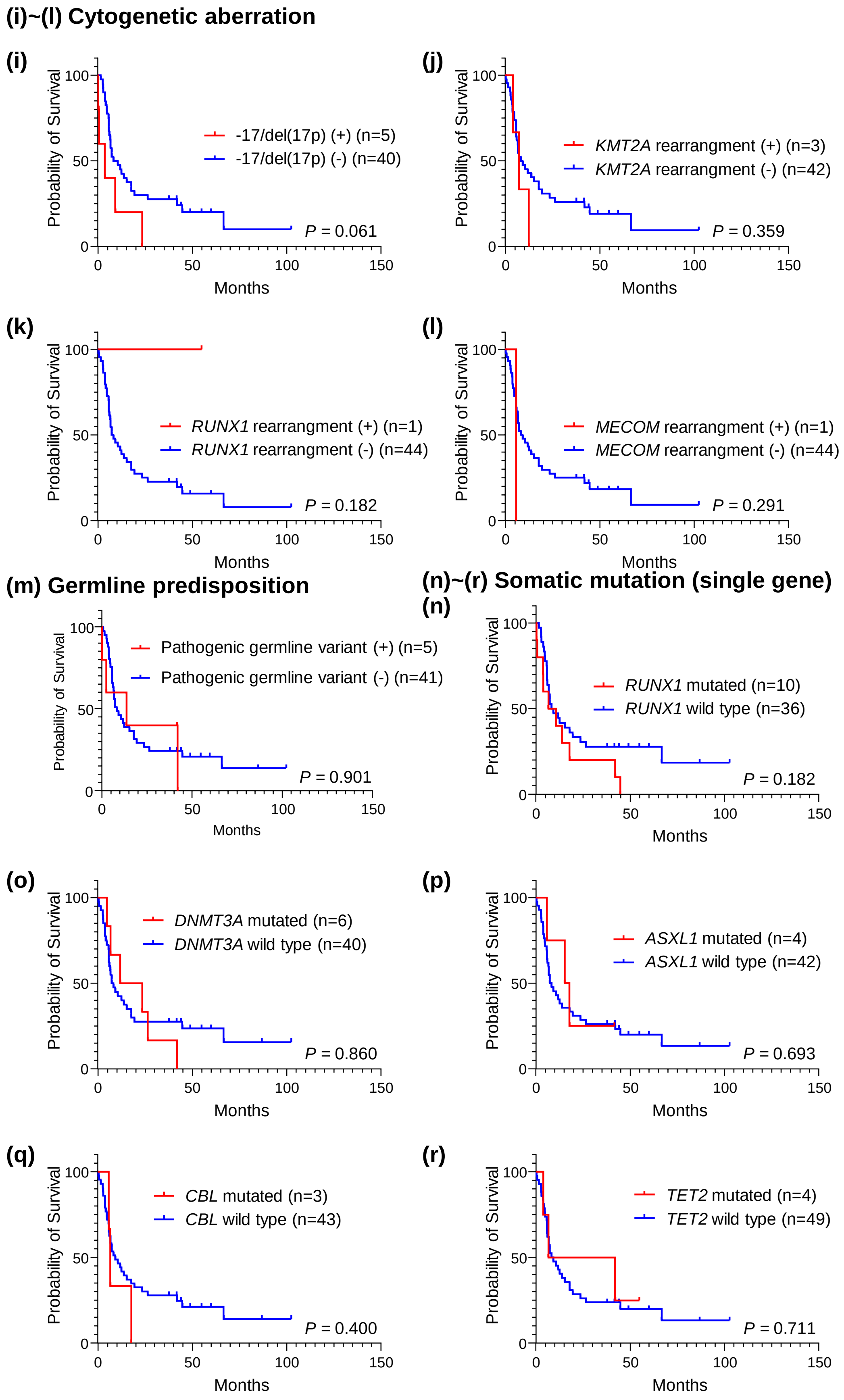

Supplementary Figure 6. Continued.

(s)~(x) Somatic mutation (gene category)

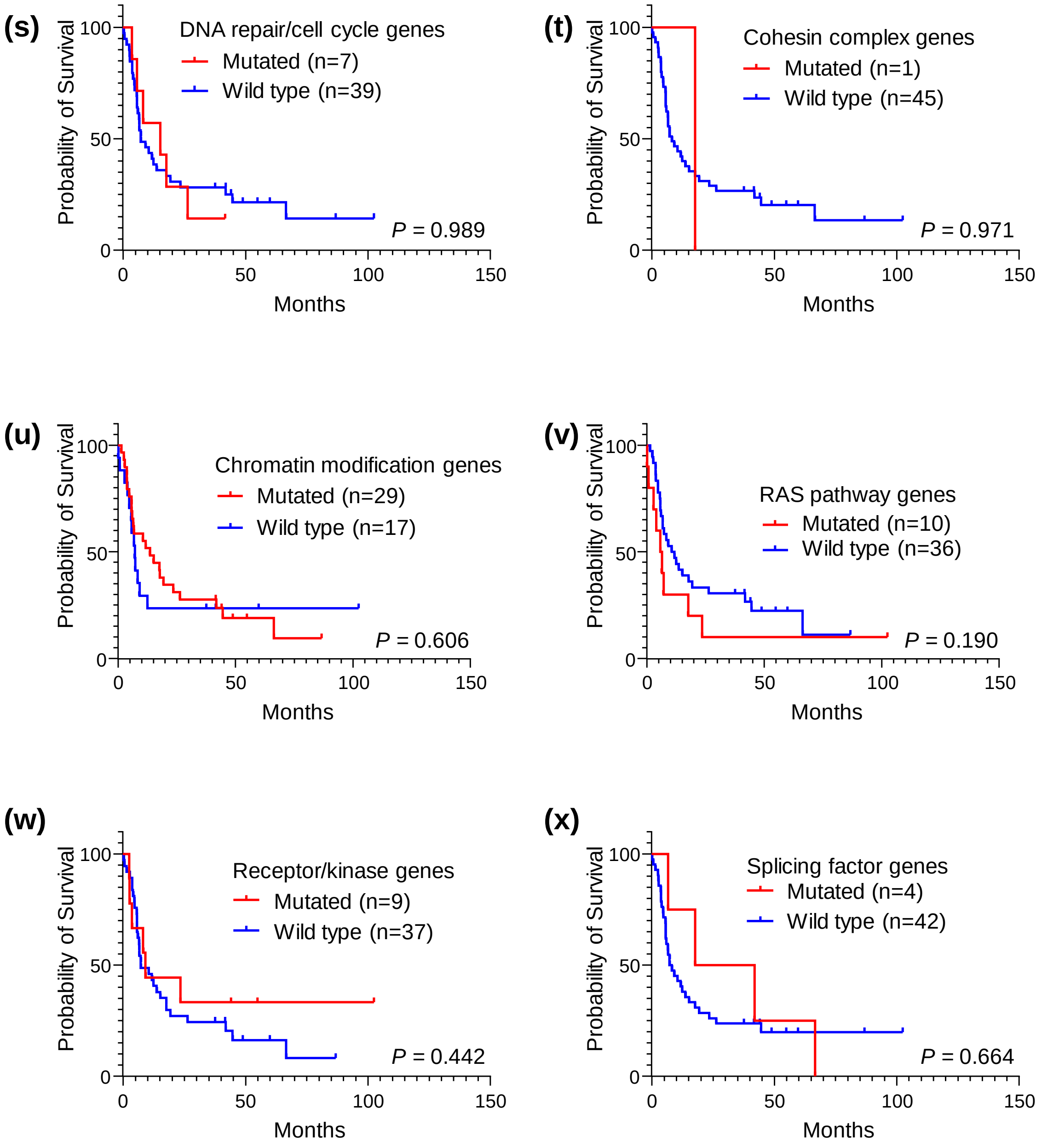

(y) Somatic variant count

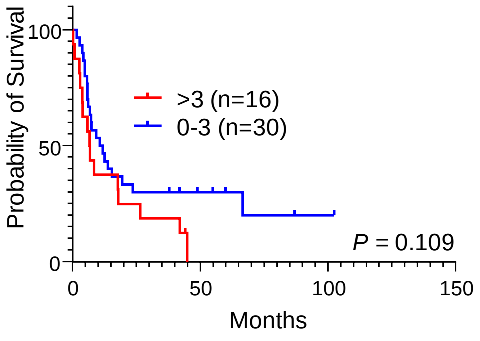

Supplementary Figure 6. Continued.

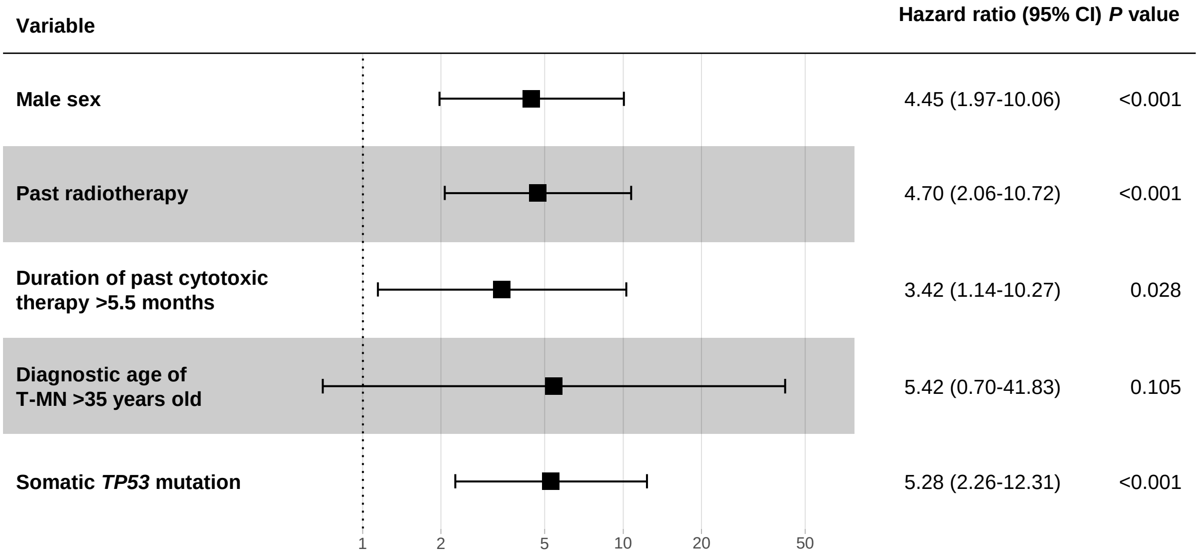

Supplementary Figure 7. Forest plot by Multiple Cox Proportional Hazards Model of 46 adult T-MN patients.

-5/del(5q), -7/del(7q), complex karyotype, and somatic variant in transcription factor genes were deleted from explanatory variables due to their multicollinearity with somatic *TP53* variant.

Abbreviations: PBSCT, peripheral blood stem cell transplantation; CI, confidence intervals.

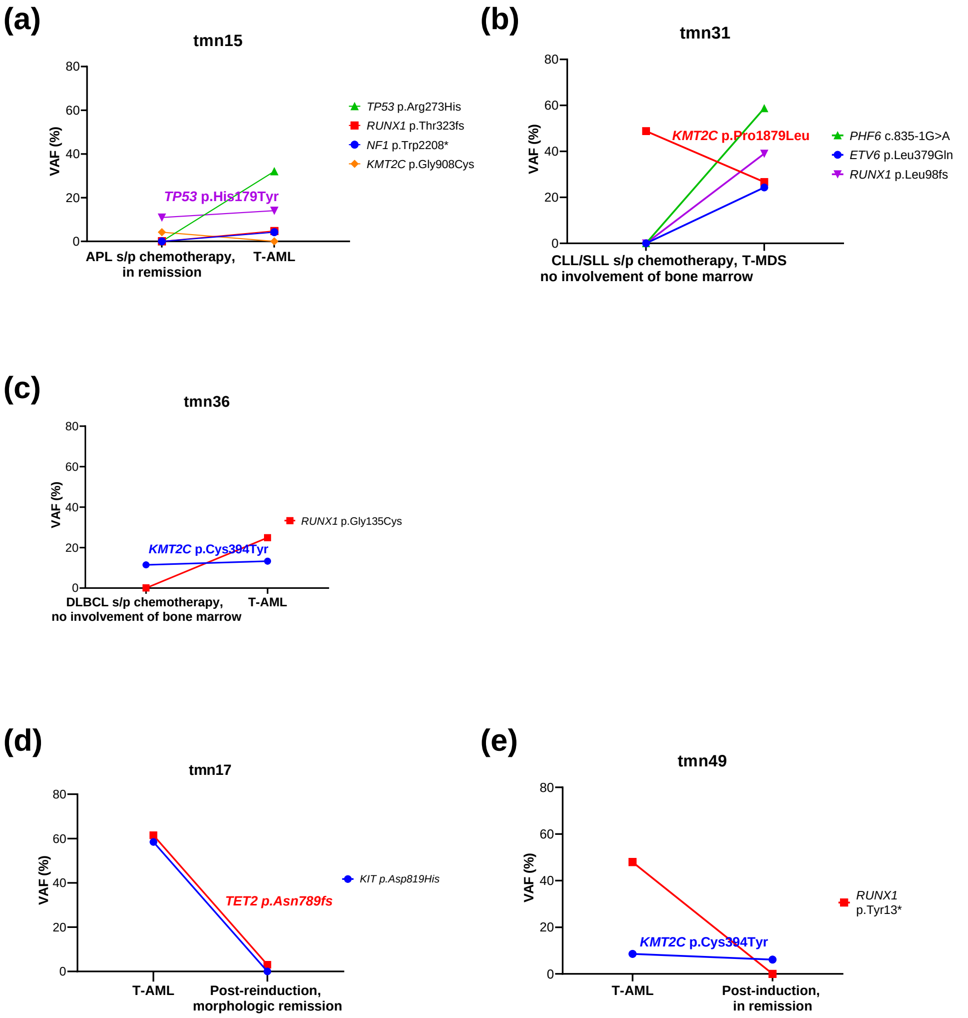

Supplementary Figure 8. Findings of pre-existing clonal hematopoiesis of indeterminate potential and constant or residual somatic variants in morphologic remission in T-MN.

(a) The tmn15 patient harbored CHIP (*TP53* H179Y, VAF 10.94%) after chemotherapy for acute promyelocytic leukemia (APL) as a primary disease. When the patient presented T-AML, the burden of the *TP53* variant did not change dramatically.

(b) The tmn31 patient harbored CHIP (*KMT2C* P1879L, VAF 48.80%) after chemotherapy for chronic lymphocytic leukemia/small lymphocytic lymphoma (CLL/SLL) as a primary disease. In the occurrence of T-MDS, the burden of the variant decreased.

(c) The tmn36 patient showed CHIP (*KMT2C* C394Y, VAF 11.49%) after chemotherapy for diffuse large B-cell lymphoma (DLBCL) as a primary disease. When the patient presented T-AML, the burden of the *KMT2C* variant burden did not change dramatically.

(d) The tmn17 patient harbored somatic variant as *TET2* N789fs and *KIT* D816H in T-AML. The patient did not achieve remission in induction therapy, and re-induction therapy was performed. After that, the BM exam showed morphologic remission but the *TET2* variant did not diminish with 3.02% VAF.

(e) The tmn49 patients showed a constant burden of *KMT2C* C394Y somatic variant between the T-AML and the morphologic remission status.

Abbreviation: s/p, status post.
